# The Lateral Preoptic Area and Its Projection to the VTA Regulate VTA Activity and Drive Complex Reward Behaviors

**DOI:** 10.3389/fnsys.2020.581830

**Published:** 2020-11-03

**Authors:** Adam Gordon-Fennell, Lydia Gordon-Fennell, Stève Desaivre, Michela Marinelli

**Affiliations:** ^1^Department of Neuroscience, College of Natural Sciences, University of Texas at Austin, Austin, TX, United States; ^2^Department of Neurology, Dell Medical School, University of Texas at Austin, Austin, TX, United States; ^3^Department of Psychiatry, Dell Medical School, University of Texas at Austin, Austin, TX, United States; ^4^Division of Pharmacology and Toxicology, College of Pharmacy, University of Texas at Austin, Austin, TX, United States

**Keywords:** dopamine, reinforcement, valence, hypothalamus, ventral tegmental area, lateral preoptic area

## Abstract

The ventral tegmental area (VTA) underlies motivation and reinforcement of natural rewards. The lateral preoptic area (LPO) is an anterior hypothalamic brain region that sends direct projections to the VTA and to other brain structures known to regulate VTA activity. Here, we investigated the functional connection between the LPO and subpopulations of VTA neurons and explored the reinforcing and valence qualities of the LPO in rats. We found that the LPO and the LPO→VTA pathway inhibit the activity of VTA GABA neurons and have mixed effects on VTA dopamine neurons. Furthermore, we found that the LPO supports operant responding but drives avoidance, and we explored the apparent discrepancy between these two results. Finally, using fiber photometry, we show that the LPO signals aversive events but not rewarding events. Together, our findings demonstrate that the LPO modulates the activity of the VTA and drives motivated behavior and represents an overlooked modulator of reinforcement.

## Introduction

The lateral preoptic area (LPO) is an understudied region of the hypothalamus that is deeply interconnected with the brain reward system. The LPO contains GABA and glutamate neurons that project to numerous brain regions known to be important regulators of reward, including the lateral habenula, rostromedial tegmental nucleus, and the ventral tegmental area (VTA; Phillipson, 1979; Kalló et al., 2015; Yetnikoff et al., 2015). The VTA contains dopamine, GABA, glutamate, and dual-expressing populations (Barker et al., 2016, 2017; Root et al., 2016), that project throughout the limbic forebrain (Matsuda et al., 2009; Russo and Nestler, 2013; Aransay et al., 2015; Barker et al., 2016). VTA_Dopamine_ neurons are important mediators of motivated behaviors (Ikemoto and Panksepp, 1999); thus it is possible that through direct and indirect connections with the VTA, the LPO may regulate these behaviors as well.

Previous work from our lab showed that stimulation of the LPO decreases VTA_GABA_ firing and increases VTA_Dopamine_ firing; it also produces reinstatement of reward-seeking (Gordon-Fennell et al., 2020), which supports the hypothesis that the LPO can drive behavior through disinhibition of dopamine neurons (Subramanian et al., 2018). From these data, it is unclear if the LPO modulates the VTA through direct or indirect projections to the VTA (Matsuda et al., 2009; Russo and Nestler, 2013; Aransay et al., 2015; Barker et al., 2016). It is also unknown if the LPO and its pathway to the VTA play a role in other reward-related behaviors. Furthermore, the literature contains limited evidence of the role of the LPO in motivated behaviors and affective valence. For example, early work found that electrical stimulation of the LPO is reinforcing (Fouriezos et al., 1987), suggesting the LPO or at least a fiber bundle passing through the LPO supports motivated behavior. However, a recent study showed that stimulating the LPO→VTA pathway with channelrhodopsin (ChR2) failed to produce reinforcement (Gigante et al., 2016). In terms of valence, a recent study showed that stimulating the LPO with bicuculline produces conditioned place-preference, suggesting the LPO may drive positive valence. Therefore, the capacity of the LPO and LPO→VTA pathway to regulate these behaviors remains unclear.

Studies examining the activity of the LPO in response to rewarding and aversive events are also inconclusive. Recording the LPO during Pavlovian conditioning revealed that about 25% of LPO neurons respond to rewarding events such as administration of glucose (Ono et al., 1986). However, this response is mixed, with about half of these neurons showing excitation and the other half showing inhibition. The same mixed-effects occur in response to aversive stimuli, such as electric foot-shock and tail pinch (Ono et al., 1986). The diverse responses of LPO neurons to rewarding and aversive events and the low sample sizes of previous experiments make it unclear how these events shape LPO activity at the population level.

Here, we present a battery of experiments to measure the functional connectivity between the LPO and subpopulations of neurons in the VTA, the behavioral effects of LPO stimulation, and how the LPO responds to rewarding and aversive events. We found that stimulation of the LPO and LPO→VTA pathway inhibits VTA_GABA_ neurons and drives mixed effects on VTA_Dopamine_ neurons. In our behavioral experiments, we found that stimulation of the LPO and LPO→VTA pathway produces reinforcement. Surprisingly, using the real-time place testing (RTPT) assay, we found that stimulation of the LPO and LPO→VTA pathway drives avoidance behavior despite being reinforcing within the same assay, which challenges the standard interpretation of the RTPT assay. Finally, using fiber photometry, we found that the LPO signals in response to aversive events but not rewarding events. The data presented in this manuscript indicate that the LPO can regulate the VTA, can drive reinforcement behavior, and may mediate behavioral responses to aversive events.

## Materials and Methods

A list of materials used is provided in table-form (Supplementary Table S1).

### Subjects

Male Sprague–Dawley rats were acquired from Envigo and housed 2–3 per cage on a reverse 12 h dark-light cycle with *ad libitum* access to water and laboratory chow (LabDiet, St. Louis, MO, USA). Rats weighed between 250 and 300 g upon arrival. All experiments were performed during the rat’s dark cycle. Procedures were done following The National Institutes of Health Guide for the Care and Use of Laboratory Animals and were approved by the Institutional Animal Care and Use Committee of The University of Texas at Austin.

### Drugs and Viral Vectors

Isoflurane, 0.9% saline, flunixin meglumine, and cefazolin were from Henry Schein (Dublin, OH, USA); phosphate-buffered saline (PBS), sucrose, paraformaldehyde (PFA), and fast green were from Sigma–Aldrich (St. Louis, MO, USA); betadine was from Purdue Products L.P. (Stamford, CT, USA); magnesium- and calcium-free PBS were from Thermo Fisher Scientific (Waltham, MA, USA).

The following adeno-associated viral vectors were obtained from UNC Viral Vector Core: AAV5/hSyn-ChR2(E123A)-eYFP (ChR2; titer: 3.7e12 or 5.3e12); rAAV5/EF1a-DIO-hChR2/(H134R)-eYFP (DIO-ChR2-eYFP; titer: 5e12); AAV5/hSyn-eNpHR3.0-eYFP (NpHR; titer: 5e12, diluted 1:4 in aCSF); rAAV5/hSyn-GCaMP6f (titer: 5.43e12); AAV5/hSyn-mCherry (mCherry, titer: 4.8e12 or 2.5e12); rAAV5/hSyn-eGFP (GFP, titer: 3.6e12). All vectors were aliquoted and stored at −80°C upon arrival. Prior to injections, aliquots were removed from the freezer, stored at 4°C, and used within 1 week of thawing.

From Institut de Génétique Moléculaire de Montpellier (Montpellier, France), we obtained CAV-2 Cre (promoter: CMV + SV40 polyA tail; titer: 1.25e13) and it was diluted 1:10 in magnesium- and calcium-free PBS (final titer: 1.25e12), aliquoted at 5 μl, and refrozen at −80°C. On the day of injection surgeries, aliquots were removed from the freezer and were used within 12 h of thawing.

### Surgical Procedures

#### General Surgical Procedures

For all electrophysiology recordings and surgical procedures, anesthesia was induced by placing rats in an induction chamber (E–Z Anesthesia, Palmer, PA, USA) filled with 5% isoflurane. Following induction, rats were transferred to a stereotaxic apparatus (David Kopf Instruments, Tujunga, CA, USA) and connected to a stereotaxic breather (E–Z Anesthesia) that delivered 2–2.5% isoflurane regulated by a vaporizer (E–Z Anesthesia). Throughout surgery, we monitored breathing rate and pinch reflex, and adjusted the level of isoflurane anesthesia when necessary. For electrophysiology experiments, body temperature was recorded with a rectal probe and maintained by a heating pad (Kent Scientific, Torrington, CT, USA).

During surgery and the day following surgery, rats received an injection of analgesic (flunixin meglumine, 5 mg/kg/ml, s.c.) and antibiotic (cefazolin, 100 mg/kg/ml, s.c.). In a small number of cases, on the day following surgery, no antibiotic was provided (16 out of 145 rats), and/or a lower dose of analgesic was provided (28 out of 145 rats; flunixin meglumine, 2.5 mg/kg/ml, s.c.).

#### Viral Injection

Rat heads were shaved with clippers (Andis Company, Sturtevant, WI, USA) and cleaned with 10% betadine (Purdue Products L.P.). We injected a local anesthetic 2% mepivacaine (TCI America, Portland, OR, USA) in the scalp, made an incision, and gently removed the tissue overlying the skull. The skull was leveled by adjusting the incisor bar. A small burr hole was made overlying the injection target and the dura at the site was removed. The stereotaxic arm was angled at 18° for targeting the LPO (final coordinate: AP: −0.12 mm, ML: −1.4 mm, DV: −8.6 mm, relative to Bregma) and 10° for targeting the VTA (final coordinate: AP: −5.4 mm, ML: −0.6 mm, DV: −8.3 mm, relative to Bregma). After viral injection surgeries, the incision was closed with surgical staples (Braintree Scientific Inc., Braintree, MA, USA) and covered in antibiotic ointment (Medique Products, Fort Myers, FL, USA).

For behavioral experiments, viral constructs were injected using a pulled glass pipette (~30 μm inner tip diameter) coupled to a Nanoject II (Drummond Scientific Company, Broomall, PA, USA) that was lowered into the target brain region and allowed to rest in place for a 1–5 min pre-injection wait period. We injected a total of 165.6–179.4 nl of viral construct throughout 5–10 min, which was then followed by a 5–10 min post-injection wait period to allow for diffusion before slowly retracting the injection pipette.

For experiments that virally isolate the LPO→VTA projection, we used the protocol above to inject the VTA with 500 nl of CAV-2 Cre over 10 min and the LPO with 303.6 nl of DIO-ChR2-eYFP over 11 min. Injections were performed serially over a single surgery session.

For experiments recording neuron activity during optogenetic stimulation of the LPO, we either injected using the protocol above or, in a small number of cases (16 out of 145 rats), we injected using a 30G injection cannula coupled to a 5 μl syringe (Hamilton, Reno, NV, USA) driven by a microinjection pump (Harvard Apparatus, Holliston, MA, USA). In these cases, there was no pre-infusion wait period, only a 300–500 nl injection over 4–6 min and a post-injection wait period of 5–7 min. In a small subset of experiments, we injected rats with a 1:1 cocktail of ChR2 and hM3Dq. The data from these recordings did not differ from data collected with ChR2 expression alone; therefore, the data were pooled. However, no results were included following hM3Dq activation *via* CNO. For experiments validating optogenetic stimulation of the LPO, rats received an intra-LPO injection with a vector cocktail of 3:5 ChR2 and hM3Dq. This cocktail of ChR2 and hM3Dq was used to validate hM3Dq-mediated excitation for another project (Gordon-Fennell et al., 2020); however, all illumination-driven responses reported in this article were recorded before local CNO administration, and all neurons were recorded >30 min after and >300 μm away from a local injection of 30–60 nl CNO.

#### Fiber Implantation

For rats undergoing optogenetic behavioral experiments, immediately following the viral injection, we implanted a ~8 mm 200 μm 0.39 NA fiber (Thorlabs, Newton, NJ, USA) attached to a 1.25 mm stainless steel ferrule (Thorlabs) 0.3–0.6 mm above the viral injection site. For rats undergoing calcium recording, we implanted an 8 mm 400 μm 0.48 NA fiber attached to a 2.5 mm stainless steel ferrule (Doric Lenses Inc., Quebec, QC, Canada) 0.3 mm above the viral injection site. Fibers were fixed to skull screws with a C&B Metabond epoxy layer (Parkell Inc., Edgewood, NY, USA) covered with a dental cement epoxy layer (Coltène/Whaledent Inc., Cuyahoga Falls, OH, USA). Fibers were covered with custom-made ferrule covers composed of melted pipette tips or 3D-printed caps fixed to ceramic ferrule sleeves. Transmission rates were recorded for all fibers before implantation and values were used to accurately set light power for each rat to achieve desired power at the fiber tip.

### Optogenetic Stimulation and Inhibition

For behavioral experiments, rats were attached to a metal-sheathed 200 μm patch cord *via* a stainless steel 1.25 mm ferrule (Thorlabs) and 1.25 mm zirconia sleeve (Senko Advanced Components, Malborough, MA, USA) that was coupled to a fiber optic rotary joint (Doric Lenses Inc.). For experiments involving ChR2 stimulation, the rotary joint was coupled to a 450 nm laser diode (Doric Lenses Inc.) that was under TTL control; light pulses were driven by Doric Neuroscience Studio (Doric Lenses Inc.) and were delivered at 15–20 mW (measured at the fiber tip), 5 ms duration, and at 20–40 Hz, with variable train durations. For experiments involving NpHR inhibition, the rotary joint was coupled to a 520 nm laser diode (Doric Lenses Inc.) that was under TTL control; continuous illumination, 10–12 mW (measured at the fiber tip) was driven by Doric Neuroscience Studio (Doric Lenses Inc.).

For electrophysiology experiments, optrodes composed of a recording pipette (details below) and 200 μm fiber (Thorlabs) were coupled to either a 450 nm or 520 nm laser diode (details above) or a 473 nm diode-pumped solid-state laser (DPSS; Laser Glow, Toronto, ON, Canada). For laser diodes and DPSS, we used Doric Neuroscience Studio and a pulse train generator (Prizmatix, Givat-Shmuel, Israel), respectively, to control pulse parameters and timing.

### Behavior Procedures

#### Intracranial Self-stimulation (ICSS)

To determine the reinforcement properties of stimulation of the LPO and LPO→VTA pathway, rats were tested with ICSS procedures. Rats were placed in an operant chamber (41 × 24 × 21 cm, Med Associates, Fairfax, VT, USA) outfitted with two nose-holes and three infrared beam detectors to measure locomotion. Nose pokes into the “active hole” triggered delivery of 15 mW, 40 Hz, 5 ms pulses into the LPO or VTA, along with a simultaneous light cue inside the active hole. The duration of the stimulation/cue differed depending on the experiment. Responses during stimulation periods were tracked but did not count towards earning an additional stimulation. Nose pokes into the “inactive hole” had no consequences and served as a measure of non-goal-directed behavior. Throughout the session, the number and timing of active hole, inactive hole, and locomotion beam break events were recorded *via* MED-PC IV (Med Associates). Before ICSS, rats were acclimated to optic fiber coupling for at least 2 days.

To determine if LPO stimulation or inhibition is reinforcing, rats were tested for ICSS in 60 min daily sessions, on a fixed-ratio 1 schedule (1 reward/1 response) with 1 s illumination per reward. The number of sessions differed based on the experiment. To determine if rats are highly motivated to obtain LPO stimulation, a subset of rats moved from a fixed-ratio schedule to a within-session progressive-ratio procedure, where the cost for each reward increased in a semilogarithmic fashion (Figure 3G). Progressive-ratio sessions lasted for 6 h or until rats did not earn a stimulation for over 1 h. To determine if there is a duration at which LPO stimulation is no longer reinforcing, the duration of the stimulation was progressively increased from 1 s to 300 s every other day (i.e., day 1: 1 s, day 2: 1 s, day 3: 3 s, et cetera). During progressive-ratio sessions, we tracked the “breakpoint” (last ratio a rat completed to obtain a reward), in addition to the variables listed above.

**Figure 1:**
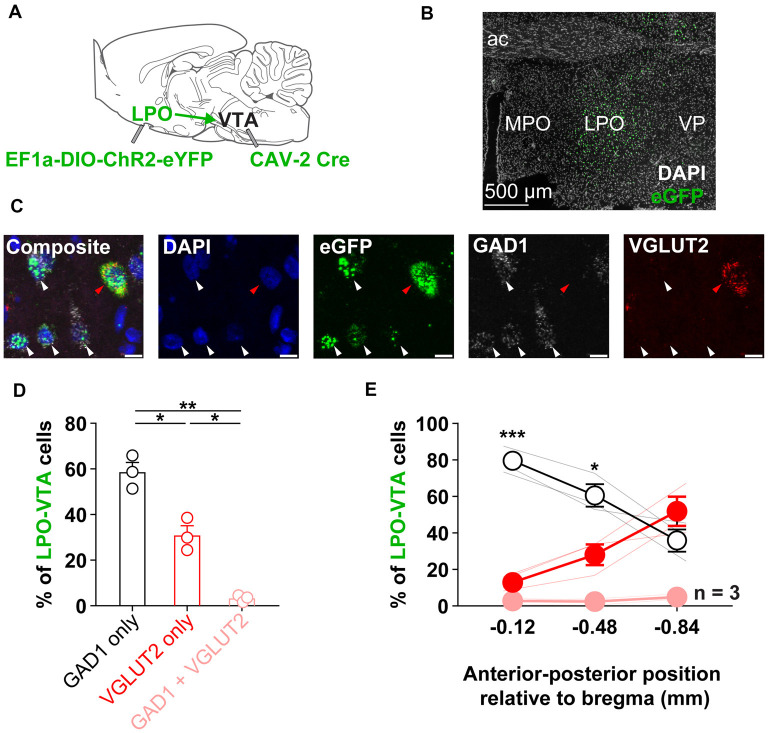
The LPO sends GABA and glutamate projections to the VTA. **(A)** Diagram of the approach to determine the relative proportions of GABA to glutamate neurons in the LPO projection to the VTA: to express a fluorescent marker selectively in LPO→VTA neurons, we injected EF1a-DIO-ChR2-eYFP in the LPO and CAV-2 Cre in the VTA. **(B)** Representative fluorescent image of eGFP expression in the LPO. **(C)** The neurotransmitter identity of LPO→VTA neurons was determined using *in situ* fluorescent hybridization for eGFP (eYFP), GAD1 (GABA), and VGLUT2 (glutamate). From left to right: composite image of all markers, DAPI only, eGFP only, GAD1 only, and VGLUT2 only; white arrows indicate eGFP + GAD1 only neurons, the red arrow indicates eGFP + VGLUT2 only neuron. **(D)** Overall, the LPO→VTA pathway contains a greater proportion of GAD1 expressing cells compared with VGLUT2 expressing cells and only contains a small population of dual GAD1 + VGLUT2 expressing cells (HSD, **P < 0.01; *P < 0.05). **(E)** The relative proportion of GAD1 and VGLUT2 within the LPO→VTA pathway varies across the anterior to the posterior extent of the LPO. The percent of LPO→VTA neurons that expressed GAD1 was greater than the percent that expressed VGLUT2 in more anterior portions of the LPO (GAD1 only vs. VGLUT2 only, HSD, ***P < 0.001; *P < 0.05). Abbreviations for (a–b): LPO, lateral preoptic area; VTA, ventral tegmental area; ac, anterior commissure; MPO, medial preoptic area; VP, ventral pallidum). In **(D)**, points depict percentages of individual rats; bars and error bars depict group mean and SEM, respectively. In **(E)**, faded lines depict values of individual rats; points and error bars depict mean and SEM, respectively. In **(C)** scale bar is equal to 10 μm.

**Figure 2:**
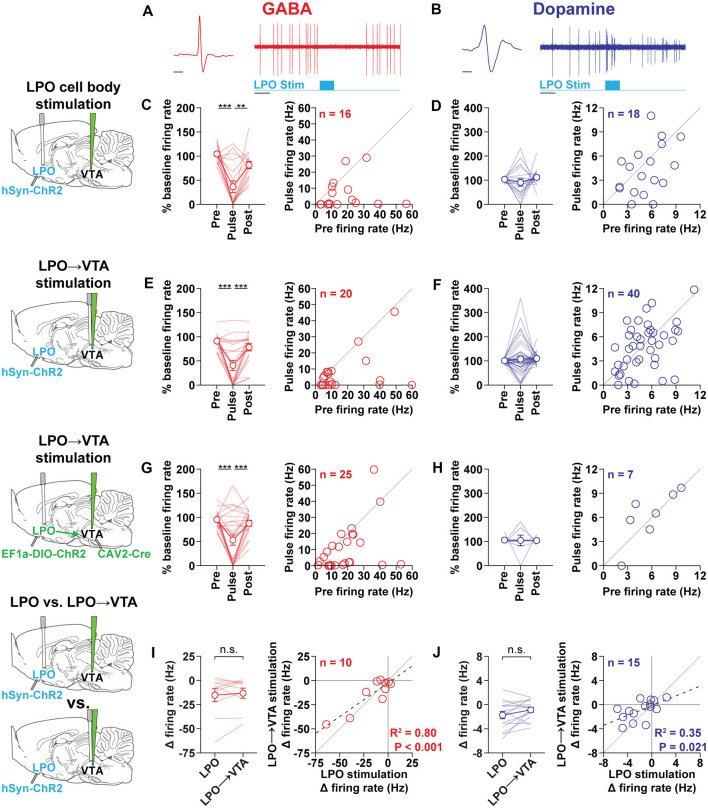
The LPO and LPO→VTA pathway modulates VTA subpopulations. **(A)** Representative VTA_GABA_ neuron (red) during stimulation of the LPO showing the extracellular waveform (left) and inhibitory response to laser stimulation of the LPO (1 s, 40 Hz, 5 ms pulses, 20 mW; right). **(B)** Representative VTA_Dopamine_ neuron (blue) during stimulation of the LPO showing the extracellular waveform (left) and stimulatory response to laser stimulation of the LPO (1 s, 40 Hz, 5 ms pulses, 20 mW; right). General format for **(C–H)**: Left plot: binned firing rate expressed as a percent of baseline firing (10 s before the first train) across peri-stimulation time bins (Pre: 2 s bin before stimulation, Pulse: 1 s bin during stimulation, Post: 2 s bin following stimulation offset; HSD, ***P < 0.001; **P < 0.01); Right plot: scatter plot of Pre vs. Pulse bin firing rate (Hz); the diagonal gray line is the identity line (i.e., slope = 1) and represents no change during stimulation. **(C,D)** In rats previously injected with hSyn-chR2 into the LPO, stimulating LPO cell bodies inhibited VTA_GABA_ neurons **(C)** and had mixed effects on VTA_Dopamine_ neurons **(D)**. **(E,F)** In rats previously injected with hSyn-chR2 into the LPO, stimulating the LPO→VTA pathway inhibited VTA_GABA_ neurons **(E)** and had mixed effects on VTA_Dopamine_ neurons **(F)**. **(G,H)** In rats previously injected with CAV-2 Cre into the VTA and EF1a-DIO-ChR2 into the LPO, stimulating cell bodies of the LPO→VTA pathway inhibited VTA_GABA_ neurons **(G)** and had mixed effects on VTA_Dopamine_ neurons **(H)**. General format for **(I,J)**: Left plot: change in firing rate (Pulse − Baseline) produced by stimulation of the LPO and LPO→VTA pathway (HSD, n.s.: *P* > 0.05). Right plot: scatter plot of change in firing rate produced by stimulation of LPO neuron bodies vs. change in firing rate produced by stimulation of the LPO→VTA pathway; the diagonal gray line is the identity line and represents no difference in change in firing produced by the two stimulation configurations. Data present in **(I,J)** is a subset of data depicted in **(C–F)**. **(I)** The stimulation of LPO and LPO→VTA pathway had similar effects on VTA_GABA_ neurons. **(J)** Stimulation of the LPO and LPO→VTA pathway had similar effects on VTA_Dopamine_ neurons. In line plots, faded lines depict values of individual rats; points and error bars depict mean and SEM, respectively. In correlation plots, the dashed line depicts the regression line and the solid line depicts a slope of 1. Abbreviations for brain diagrams: LPO: lateral preoptic area; VTA: ventral tegmental area.

**Figure 3:**
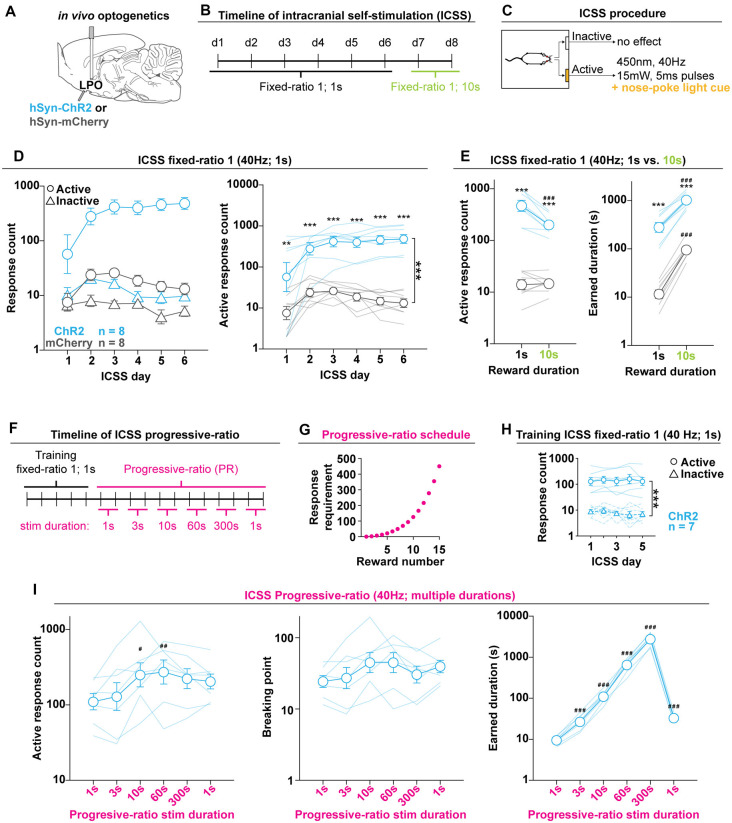
The LPO supports Intracranial self-stimulation (ICSS). **(A)** *In vivo* optogenetics setup: we injected either hSyn-ChR2 (ChR2) or hSyn-mCherry (mCherry) in the LPO and implanted an optic fiber overlaying the injection site. **(B)** Timeline of ICSS testing. **(C)** Illustration of the ICSS procedure. **(D)** Self-administration behavior during ICSS fixed-ratio 1, for stimulation duration of 1 s. Left: ChR2 (blue) and mCherry (gray) rats showed differential discrimination between the active hole (Active, circles) and inactive hole (Inactive, triangles); right: the ChR2 group made more active hole responses then the mCherry group throughout ICSS (***group effect: *F*_(1,14)_ = 68.74, *P* < 0.001). **(E)** Active hole responses over the last 2 days of responding for 1 s stimulation and 2 days of responding for 10 s stimulation. The ChR2 group decreased active hole responding, while mCherry did not (left). Both groups increased the total earned stimulation duration, however, rats in the mCherry group increased to a greater degree (right). **(F)** Timeline of progressive-ratio testing. **(G)** Progressive-ratio schedule: the cost for each subsequent reward was increased in a semilogarithmic fashion. **(H)** Training in ICSS fixed-ratio 1 for 1 s stimulation before progressive-ratio. Rats discriminated between active and inactive holes (***hole effect: *F*_(1, 6)_ = 164.07, *P* < 0.001). **(I)** Self-administration behavior during progressive-ratio indicated that increasing the duration of the stimulation led to an increase in active hole responding (left), breakpoint (mid), and stimulation duration (right). Throughout the figure, active hole responses, inactive hole responses, breaking point, and earned stimulation duration are shown on a log scale; (HSD ChR2 vs. mCherry, **P < 0.01, ***P < 0.001; HSD vs. 1 s, ^#^*P* < 0.05, ^##^*P* < 0.01, ^###^*P* < 0.001). In **(D,E,H,I)**, faded lines depict values of individual rats; points and error bars depict mean and SEM, respectively.

#### Real-Time Place Testing (RTPT)

To determine the valence of stimulating or inhibiting the LPO and LPO→VTA pathway, we tested rats in an RTPT task where rats could freely control the inter-stimulation-interval (ISI) and the stimulation-interval (SI). Before RTPT, rats were acclimated to optic fiber coupling for at least 2 days. RTPT procedures were conducted in a custom-made apparatus made of opaque Plexiglas (dimensions: 61 × 30.5 × 30.5 cm), that was virtually divided into two sides. The rat’s position in the apparatus was tracked online using Ethovision XT (Noldus Information Technology, Wageningen, Netherlands), enabling closed-loop stimulation based on the rat’s position in the apparatus. On day 1 [habituation (Hab)], rats were placed in the apparatus with identical textured floors on each side for 10 min. This session served as a habituation day to acclimate the rats to the apparatus, handling and connecting them to the patch chord. From day 2 onward, sessions lasted 20 min, and rats were placed into the same apparatus but with identical, non-textured floors. Day 2 served as a preference test (PT) to measure baseline preference bias for each side of the apparatus. In the next 3–4 days [initial pairing (Int)], one side of the apparatus was assigned to be the *initially paired side* and the other the* initially unpaired side*. Assignments were made for each rat individually to minimize the baseline bias of each group towards the initially paired side. During the initial pairing, any time a rat’s center point was detected in the initially paired side the stimulation pattern advanced and ceased the moment the center point was detected in the initially unpaired side. On the last 3–4 days [inverted pairing (Inv)], laser pairing was inverted from the initial pairing, so that the initially unpaired side now triggered stimulation and the initially paired side no longer did. This allowed us to observe a change inside preference with the change in laser-pairing contingency. To quantify the amount of preference or aversion associated with stimulation, we calculated an RTPT score by subtracting the mean time spent in the initially paired side during all days of inverted pairing from the mean time spent in the initially paired side during all days of initial pairing, then dividing by the session duration {RTPT score = [mean (initially paired side Int) – mean (initially paired side Inv)]/session duration}. This produces a score between −1 and 1, where −1 is maximal aversion, 1 is maximal preference, and 0 is no valence. Stimulation parameters varied across experiments, see main text for details.

#### Real-Time Place Preference With Progressive Adversity (RTPT Electricity)

To determine the reinforcing properties of stimulating the LPO and LPO→VTA pathway within the RTPT procedure, we tested rats in a modified RTPT procedure in which the optically paired side was also paired with adversity (electricity). Rats were tested as outlined above (habituation, preference test, and 4 days of initial laser pairing, 20 min/session) but in an operant chamber (Med Associates) where one side of the apparatus had a floor consisting of metal bars and the other had a floor consisting of Plexiglas. In this experiment, the initial pairing was assigned to the side of the chamber with metal bars for all rats regardless of baseline preference. The laser illumination pattern was 40 Hz, 5 ms pulses, 15 mW, 3 s trains, and 3 s inter-train interval (ITI). After the initial pairing, rats were tested for 13 additional days where we applied electricity to the side of the chamber with metal bars, which continued to be paired with optical stimulation. The electric foot-shock amplitude was progressively increased across the first 3 days (0.05, 0.10, and 0.15 mA), maintained at 0.15 mA for the next 8 days, and then stepped back down for the last 3 days (0.13, 0.10, and 0.08 mA; Figure 7B). To quantify the reinforcing properties of laser stimulation, we measured the amount of time spent on both sides of the chamber and the number of crossings into the paired side that resulted in a visit of greater than 3 s. We quantified only visits that were a minimum of 3 s because this ensures that rats received illumination within the illumination pattern (3 s trains, 3 s ITI).

**Figure 4:**
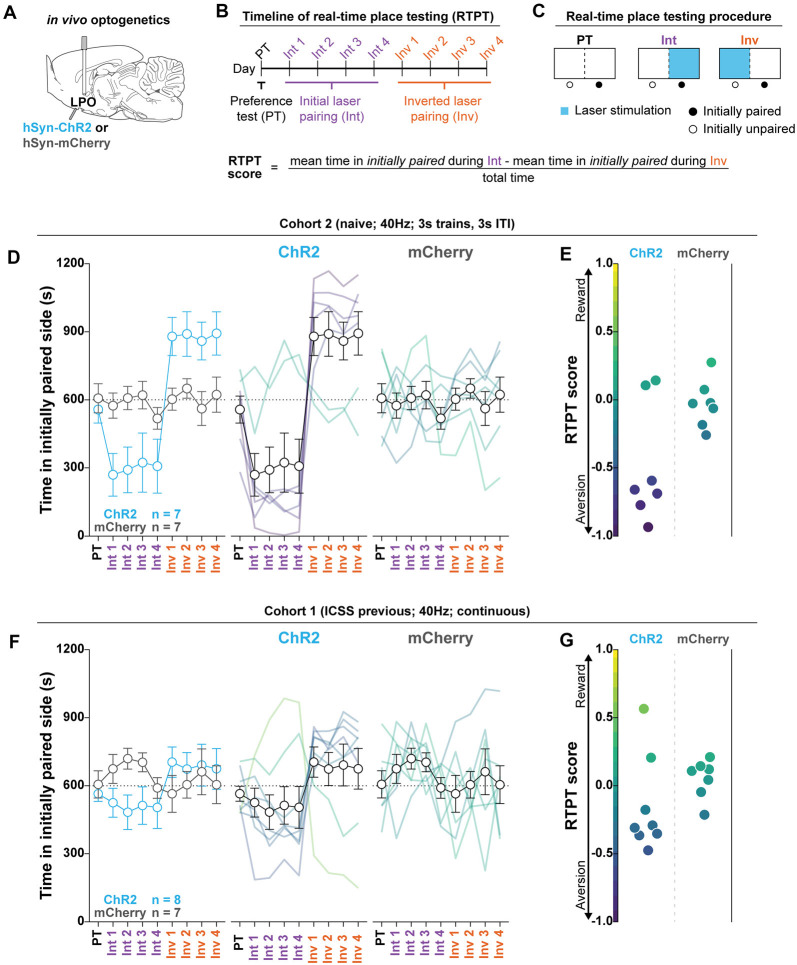
The LPO promotes real-time place aversion in the majority of rats. **(A)** *In vivo* optogenetics setup: we injected either hSyn-ChR2 (ChR2) or hSyn-mCherry (mCherry) in the LPO and implanted an optic fiber overlaying the injection site. **(B)** Timeline for real-time place testing (RTPT). **(C)** RTPT procedure and equation for RTPT score. **(D)** The mean time in the initially paired side over days of RTPT in ChR2 (blue) and mCherry (gray) groups; single rats are color-coded based on their RTPT score. The ChR2 and the mCherry groups showed differential effects across days of RTPT (group × day interaction: *F*_(8,96)_ = 5.56, *P* < 0.001), where the majority of rats in the ChR2 group show aversion, indicated by a low amount of time in the initially paired side during initial pairing and a high amount of time during inverted pairing. **(E)** RTPT scores for rats in the ChR2 and the mCherry group. **(F,G)** same as **(D,E)** for Cohort 1, which was tested for ICSS before RTPT. **(F)** On average, the ChR2 and the mCherry groups did now show differences in time spent in the initially paired side across days of RTPT (group × day interaction: *F*_(8,104)_ = 1.59, *P* = 0.14). **(G)** However, RTPT scores indicate bidirectional effects on RTPT in ChR2 rats. In **(D)** and **(F)**, faded lines depict value from individual rats; points and error bars depict mean and SEM, respectively.

**Figure 5:**
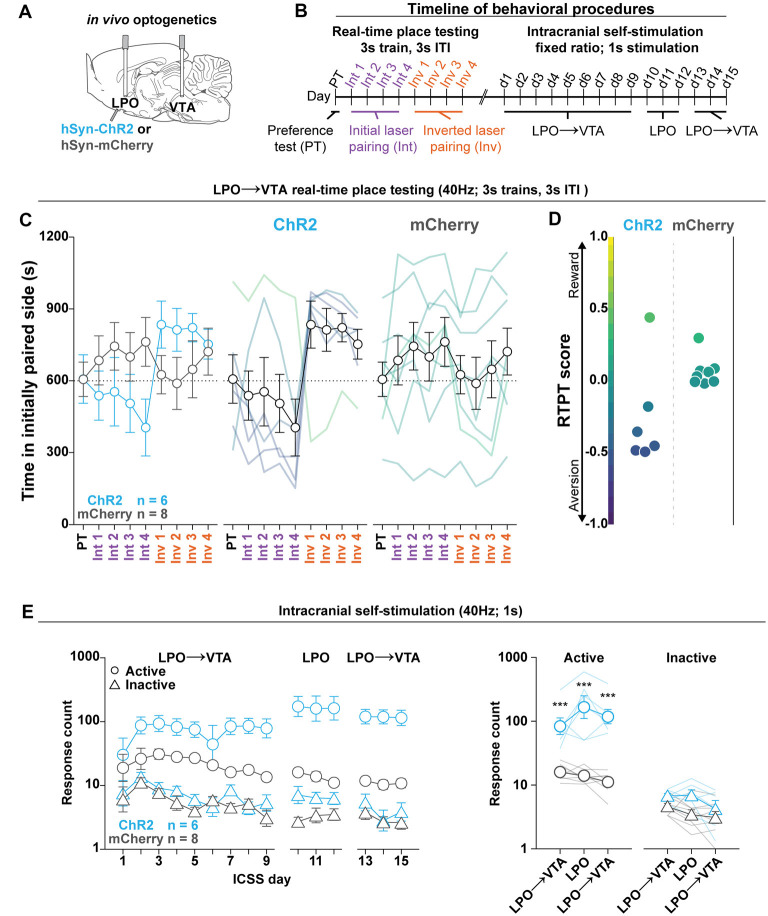
The LPO→VTA pathway supports ICSS and promotes real-time place aversion in the majority of rats. **(A)** *In vivo* optogenetics setup: we injected either hSyn-ChR2 (ChR2) or hSyn-mCherry (mCherry) in the LPO and implanted optic fibers overlying the injection site and VTA. **(B)** Timeline for RTPT and ICSS (procedure details can be found in legends for Figures 3, 4). **(C)** The mean time in the initially paired side across days of RTPT in the ChR2 (blue) and mCherry (gray) groups; single rats are color-coded based on their RTPT score. The ChR2 and the mCherry groups showed differential time spent in the initially paired side across days of RTPT (group × day interaction: *F*_(8,96)_ = 4.34, *P* < 0.001), where the ChR2 group showed aversion indicated by a low amount of time spent in the initially paired side during initial pairing and a high amount of time during inverted pairing. **(D)** RTPT scores for rats in ChR2 and the mCherry groups. **(E)** Self-administration behavior during ICSS at a fixed-ratio 1, for 1 s 40 Hz illumination in the LPO→VTA pathway and LPO. Left: the ChR2 and the mCherry groups showed differential discrimination between the active hole (Active, circles) and inactive hole (Inactive, triangles; group × hole interaction: *F*_(1,12)_ = 15.21, *P* = 0.0021); right: three-day mean responding on the active hole and the inactive hole. Relative to the mCherry group, the ChR2 group showed higher responding in the active hole for stimulation of the LPO→VTA pathway and LPO but did not show any difference in inactive hole responding (HSD, ***P < 0.001). Active hole and inactive hole responses are shown on a log scale. In **(C,E)**, faded lines depict values from individual rats; points and error bars depict mean and SEM, respectively.

**Figure 6:**
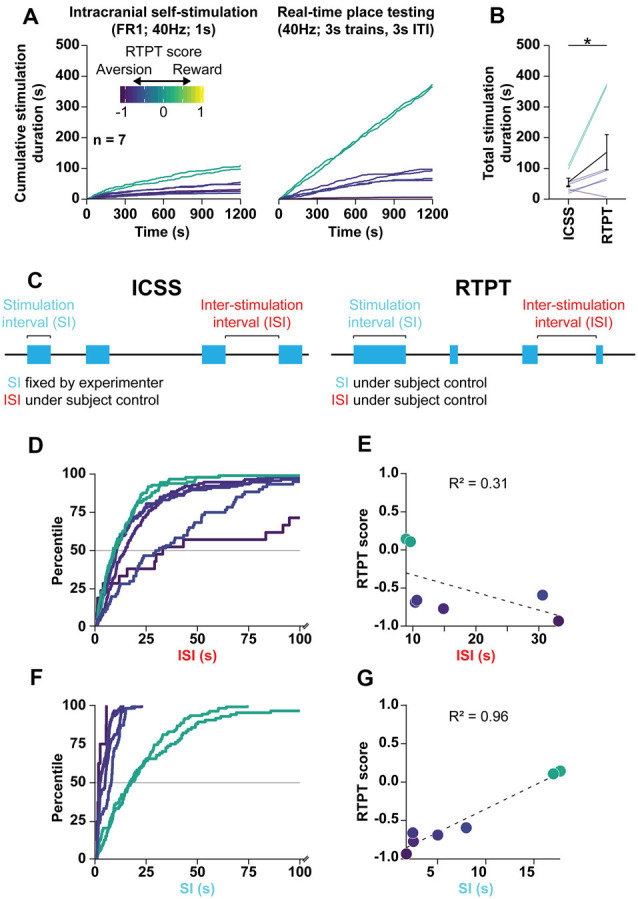
RTPT scores are the product of preferred stimulation-intervals. **(A)** Mean of cumulative stimulation duration over days 2–4 of ICSS (left) and days 2–4 of initial pairing for RTPT (right) following optogenetic stimulation of the LPO cell bodies. ICSS data are truncated to the first 20 min to compare them to RTPT over the same time scale. Rats are color-coded based on their RTPT score (see RTPT behavior in Figure 4). **(B)** The total stimulation duration earned in RTPT was higher than that in ICSS (Wilcoxon, *P < 0.05). **(C)** Diagram depicting the behavioral components that underlie the total stimulation duration within ICSS (left) and RTPT (right). In ICSS, the stimulation-interval (SI) is defined by the experimenter, while the inter-stimulation-interval (ISI) is under the animal’s control. In RTPP, both the SI and ISI are under the animal’s control and can independently contribute to the stimulation duration obtained during RTPT. **(D)** CDF for ISI within RTPT for each subject, color-coded by RTPT score. Note that the x-axis is cut off at 100 s. **(E)** Correlation between the median ISI and RTPT score indicate a poor correlation. **(F)** CDF for SI within RTPT for each subject, color-coded by RTPT score. **(G)** Correlation between the median SI and RTPT score indicates a strong correlation. In **(E,G)**, the dashed line depictsthe regression.

**Figure 7:**
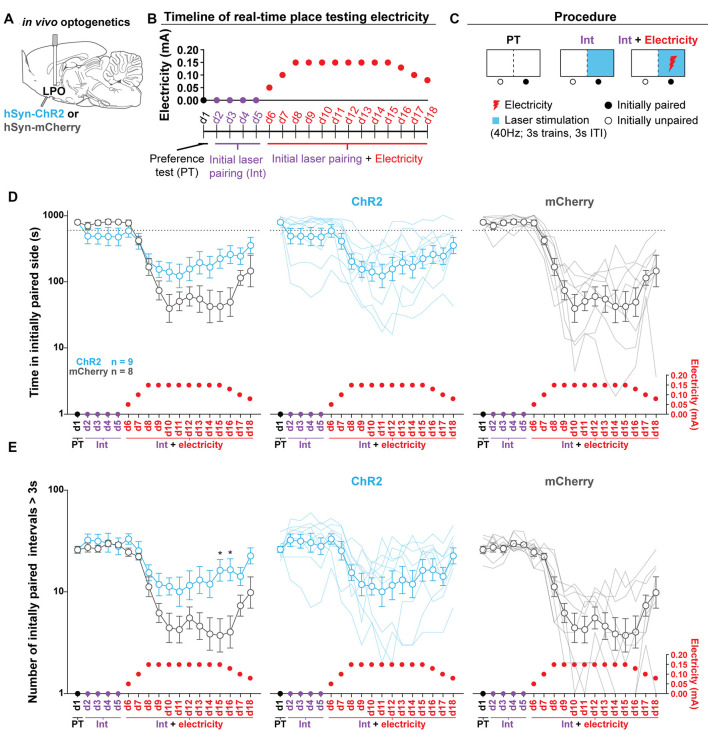
Stimulating the LPO reduces foot-shock avoidance. **(A)** *In vivo* optogenetics setup: we injected either hSyn-ChR2 (ChR2) or hSyn-mCherry (mCherry) in the LPO and implanted a fiber overlaying the injection site. **(B)** Timeline for RTPT. Note that pairing remains consistent across training and the optically paired side becomes dual-paired with electricity. **(C)** RTPT electricity procedure. **(D)** Time spent in the initially paired side across RTPT decreased across days as the initially paired side was paired with electricity (day effect: *F*_(17,255)_ = 30.34, *P* < 0.001). The mCherry group decreased to a greater degree than the ChR2 group (day × group interaction: *F*_(17,255)_ = 5.55, *P* < 0.001). **(E)** The number of initially paired intervals greater than 3 s across RTPT decreased across days as the initially paired side was paired with electricity (day effect: *F*_(17,255)_ = 26.71, *P* < 0.001). The mCherry group decreased to a greater degree than the ChR2 group (day × group interaction: *F*_(17,255)_ = 4.06, *P* < 0.001). In **(D,E)**, faded lines depict values from individual rats; points and error bars depict mean and SEM, respectively (HSD ChR2 vs. mCherry, *P < 0.05).

#### Pavlovian Conditioning for Sucrose

To determine if the LPO has time-locked signals to rewarding events and their predictive cues, rats were tested for Pavlovian conditioning for sucrose in an operant chamber (Med Associates). Before training, rats went through 4 days of acclimation to the food port containing five sucrose pellets (45 mg, Bio-Serv, Flemington, NJ, USA). These food-port acclimation sessions were 20 min long, whereas all subsequent sessions were 60 min long. During the first phase of the procedure, rats underwent concurrent magazine training (i.e., delivery of sucrose pellets into the food port) and presentation of a tone cue over 6 days (Preconditioning). During this phase, 30 sucrose pellets were delivered by the magazine and 30 tone cues (10 s, 3 kHz, 76 dB) were presented on independent 60–120 s pseudorandom inter-trial intervals (Supplementary Figure S2B). The latency from magazine delivery to port entry was tracked and analyzed to determine when rats learned the association between the sound of the magazine delivering the pellet and the presence of the pellet in the food port. After magazine and tone cue preconditioning, rats were tested in one session for the response to pellet delivery in the absence of the tone cues (Magazine test; Supplementary Figure S20C). The following day, rats were tested in one session for the response to the tone cue in the absence of pellet delivery (Tone test; Supplementary Figure S20D). Next, rats were trained over 15 days of Pavlovian reward conditioning, where 30, 10 s tone cues were paired with pellets delivered at the tone offset (trace conditioning), that was presented on 70–110 s pseudorandom inter-trial intervals (Supplementary Figure S20E). Learning was monitored by measuring the number of food port entries during the tone cue and the 10 s period immediately before cue onset. The time course of food port entries per trial was visualized by computing the density of food port entries using a kernel with a standard deviation of 0.25 s and then scaling the density by multiplying by the total number of events divided by the total number of trials (Supplementary Figure S21D). Finally, rats went through 3 days of Pavlovian conditioning where 80% of trials occurred as normal (expected), 10% of trials occurred without tone cue delivery (unexpected), and 10% of trials occurred without pellet delivery (omission; Supplementary Figure S20F). Calcium signals in the LPO were recorded during the following sessions: the first day of preconditioning; tone test; magazine test; first day and last 3 days of Pavlovian conditioning; 3 days of Pavlovian conditioning with expected, unexpected, and omission conditions (Supplementary Figure S20A).

#### Pavlovian Conditioning for Foot-Shock

Following Pavlovian conditioning for sucrose, rats were tested in a novel fear conditioning chamber (26 × 26 × 30 cm, Ugo Basile, Gemonio, Italy), where a 20 s tone (5 kHz, 74 dB) was paired with a 2 s, co-terminating electric foot-shock (0.7 mA, scrambled) in a 20 min session (Supplementary Figure S20G). Rats were tested for one session per day for 3 days. Each session started with a 5 min baseline period to allow rats to acclimate and to allow for baseline photobleaching, followed by 5 tone/foot-shock pairings separated by a 100–140 s random interval. Sessions were recorded using an analog camera and were digitized using Ethovision XT (Noldus Information Technology). On rare occurrences, the shock delivery malfunctioned and there was no indication that the subject received a shock; these trials were removed from the analysis. Calcium signals in the LPO were recorded during all 3 days of fear conditioning.

### Extracellular Recording

#### General Procedures

Extracellular recordings were performed in isoflurane-anesthetized rats, as described previously (Gordon-Fennell et al., 2020). The stereotaxic arm was angled at 18° for targeting the LPO (final coordinate: AP: −0.12 mm, ML: −1.4 mm, DV: −8.6 mm, relative to Bregma), and 0° for targeting the VTA (final coordinate: AP: −5.4 mm, ML: −0.6 mm, DV: −8.3 mm, relative to Bregma). The timing of optogenetic stimulation pulses was recorded *via* a TTL output that was fed from the laser into the digitizer. VTA neurons were classified as putative dopaminergic neurons based on established extracellular recording criteria: (1) firing rate between 1 and 10 Hz; (2) triphasic (+/−/+) waveform; and (3) wide extracellular waveforms (>2.4 ms, measured from start to end of the spike when using a 400–500 Hz band-pass filter (Einhorn et al., 1988) and >1.1 ms, from start to trough when using a 50–800 Hz band-pass filter (Ungless and Grace, 2012; Marinelli and McCutcheon, 2014). Using these criteria, we are ~90% accurate at detecting neurons containing tyrosine hydroxylase (Ungless and Grace, 2012). These neurons will be referred to as dopamine neurons from this point onwards (Figure 2B). VTA neurons were classified as putative GABAergic neurons when they failed to reach dopaminergic criteria. These neurons were often biphasic and exhibited high firing rates (>10 Hz). These neurons will be referred to as GABA neurons from this point onwards (Figure 2A).

To measure baseline firing characteristics, all neurons were recorded for 2–3 min before optogenetic manipulations. For ChR2, illuminations consisted of six 1 s-long trains (40 Hz, 5 ms pulses) with a 9 s ITI. Stimulation power ranged from ~1–20 mW (mean: 14.51; SD: 7.37) when delivered at the recording site, and 10–20 mW (mean: 19.55; SD: 2.10) when delivered at a distant site. For stimulation at the recording site, power was decreased to minimize light artifacts. There was no effect of the stimulation power, so all data were pooled across the power. For NpHR, illumination was a continuous 2 mW pulse with varying durations (50 ms, 1 s, 10 s, or 60 s).

For all electrophysiology experiments with 1 s manipulations, the effect of illumination was determined by binning the data into 2 s before illumination (Pre), 1 s during illumination (Pulse), and 2 s post illumination (Post), and expressing firing rate in Hz. For NpHR illumination of 10 s and 60 s, the Pre and Post bin durations equaled the length of the illumination. Binned firing rates were then expressed relative to baseline (the 10 s preceding the first bin) to determine relative changes. Neurons were classified as showing an effect in response to laser illumination using a paired *t*-test comparing Pre vs. Pulse firing over the six trains. For statistical analysis, the time-periods (Pre, Pulse, and Post) are defined as “time-bins.”

After recording, fast-green was deposited at the final pipette position using 28.6 mA cathodal current (Fintronics Inc., Orange, CT, USA) to back-calculate the position of recorded cells (see “Histology” section below for details).

#### Functional Connectivity Between the LPO and VTA

Rats received an injection of DIO-ChR2-eYFP into the LPO, which was followed by an incubation period of >9 weeks to allow for adequate presynaptic ChR2 expression within the VTA. Neurons were recorded in the VTA during stimulation of LPO cell bodies or LPO→VTA presynaptic terminals. In a separate experiment, the LPO→VTA pathway was isolated by injecting DIO-ChR2-eYFP into the LPO and CAV-2 Cre into the VTA. This was followed by an incubation period of >20 weeks to allow for adequate ChR2 expression. This combinatorial approach produced an expression of ChR2 only in LPO neurons that have presynaptic terminals within the VTA. Neurons were recorded in the VTA during LPO→VTA cell body stimulation.

#### Validation of ChR2 Stimulation and NpHR Inhibition

To validate ChR2-mediated excitation, rats received an injection of ChR2 into the LPO that was followed by an incubation period of >8 weeks. Neurons were recorded in the LPO during LPO cell body illumination. Multiple illumination parameters were used to determine if LPO neurons are excited across parameters. First, 10 ms pulses were delivered at 0.2–0.5 Hz for 20 pulses. Neurons were classified as expressing ChR2 if they had <5 ms average latency to spike, <2 ms latency jitter (latency standard deviation), and >80% fidelity (number of spikes/number of pulses). To determine if trains of illumination drove excitation, neurons were also stimulated at high-frequency trains (20 and 40 Hz, 5 ms pulses, 1 s train, 9 s ITI).

To validate NpHR-mediated inhibition, rats received an injection of NpHR into the LPO that was followed by an incubation period of >9 weeks. Neurons were recorded in the LPO during LPO cell body illumination. To ensure that illumination inhibited neurons across parameters, we delivered 2 mW illumination over multiple durations (50 ms, 1 s, 10 s, or 60 s). To determine if illumination inhibited the firing rate and if the offset of illumination drove rebound stimulation, we analyzed the firing rate both during and after illumination.

### Calcium Recording and Analysis

Calcium signals in the LPO were recorded using fiber photometry as adapted from Gunaydin et al. (2014). Rats were attached to a 1 m metal-sheathed 400 μm 0.48 NA patch chord coupled directly to a filter cube (Doric Lenses Inc.). GCaMP signals were monitored using a blue 465 nm LED (Doric Lenses Inc.) sinusoidally modulated at 208.62 Hz with a mean fiber power of 30–50 μW. Autofluorescent signals were monitored using a violet 405 nm LED (Doric Lenses Inc.) modulated at 530.48 Hz with a mean fiber power of 30–50 μW. Fluorescence of both channels was detected on modified Newport femtowatt detectors (Doric Lenses Inc.) and demodulated using a fiber photometry console (Doric Lenses Inc.). Signals were filtered with a low pass filter (12 Hz) and were digitized at 1,200 ksps.

The calcium and autofluorescent channels were processed *post hoc* using custom MATLAB (MathWorks, Natick, MA, USA) and *R* scripts (RStudio Inc., Boston, MA, USA) by taking a 1 s moving median on both channels, computing *z*-scores [(*x* – μ)/σ], and then down-sampling to 20 Hz. Peri-event histograms were created by subtracting the median baseline *z*-score from each sample and then averaging across trials. We did not perform subtraction of a fitted autofluorescent channel because we found changes in power that were time-locked to behavior both in the calcium and autofluorescent channels, except that the changes in the autofluorescent channel were on a smaller scale, resulting in poor fitting and ineffectual subtraction. Instead of performing a fitted subtraction, we assessed effects in the *z*-score of the calcium channel by analyzing within-subject comparisons with the *z*-score of the autofluorescent channel.

### Fluorescent *In Situ* Hybridization

After 2 months of incubation to allow for adequate viral expression, rats were deeply anesthetized with isoflurane, decapitated, and their brains were removed and frozen in 2-methyl butane (Sigma–Aldrich) on dry ice. After 5–10 s in 2-methyl butane, the brains were blocked into brain molds using optimal cutting temperature (OCT) compound (Thermo Fisher Scientific) wrapped in aluminum foil and placed on dry ice. We performed the *in situ* hybridization assay using the RNAscope Fluorescent Multiplex Detection Reagents (Advanced Cell Diagnostics Inc., Hayward, CA, USA), according to the guidelines provided by the manufacturer with a few adjustments: Simport Scientific EasyDip Slide Staining Jars (Thermo Fisher Scientific) were used in place of Tissue-Tek Staining Dishes; 75 μl of probe mixture (mixed the day of the assay) was applied to each full rat section instead of the suggested 120 μl; for all amplifiers, we used approximately two drops instead of the suggested four drops; and DAPI Fluoromount-G (SouthernBiotech, Birmingham, AL, USA) was used in place of the combination of DAPI and fluorescent mounting medium. We used the following target probes (Advanced Cell Diagnostics Inc.): eGFP for eYFP, Rn-Slc17a6-C2 for VGLUT2, and Rn-Gad1-C3 for GAD1. Amp-4 Alt-A was used to fluorescently label the probes: eGFP (Alexa488), VGLUT2 (Atto550), and GAD1 (Atto647). Slides were imaged with a Nikon A1R confocal microscope (Nikon, Melville, NY, USA). Large images were taken at 20× with 10 *z*-steps of 1 μm and stitched together with a 25% overlap. The maximum intensity projection was produced using NIS-Elements to be used for further analysis. The location of the target region was determined manually by mapping the appropriate image of the Paxinos and Watson rat brain atlas (Paxinos and Watson, 2007) onto the fluorescent image using the Big Warp plugin in ImageJ (National Institute of Health, Bethesda, MD, USA). Neuron counting was performed manually using the Cell Counter plugin in ImageJ. A neuron was determined to be expressing a target gene if it contained five or more fluorescent dots in or surrounding a DAPI-stained nucleus.

### Histology

The location of optic fibers and viral expressions were determined after each experiment. Rats were deeply anesthetized with isoflurane and transcardially perfused with PBS followed by 4% PFA. For experiments with optic fiber implants, rats were decapitated and their skulls were post-fixed for 24 h with fibers intact, after which the fibers were extracted by first using a Dremel (Dremel, Racine, WI, USA) to remove the implant surrounding the fiber and then using a hemostat to remove the optic fiber. Brains were then removed and post-fixed for an additional 24 h. For experiments without optic fiber implants, brains were removed and post-fixed for 24 h. For all experiments, after post-fixing, brains were transferred to 20% sucrose until they sank. Brains were serially sectioned at 40 μm on a cryostat and collected into well plates filled with a cryoprotectant (24% glycerol and 29% ethylene glycol in PBS). The sections were imaged in well plates with a fluorescent stereomicroscope (Carl Zeiss, Oberkochen, Germany) using a consistent exposure for each subject to allow for fluorescent intensity comparisons across sections.

The fast-green spot and/or optic fiber tip locations were located and imaged and then mapped onto a reference atlas. For electrophysiology experiments, the position of other recorded neurons was back-calculated relative to the fast-green location.

### Statistical Analysis and Data Visualization

We analyzed results using repeated-measures ANOVA followed by *post hoc* using Tukey’s honest significant differences test (HSD), student’s *t*-test, and Pearson product-moment correlation. In cases where the data were not normally distributed, as determined by the Shapiro–Wilk normality test, a paired Wilcoxon rank-sum test was used in place of a student *t*-test. Sample sizes were determined based on preliminary experiments or on effect size (partial eta squared = 0.01–0.25 for repeated measures or main effects ANOVA). In all plots, error bars indicate the standard error of the mean (SEM).

All statistical analysis was conducted in R. We used the “afex” package to compute ANOVAs, the “emmeans” package to compute HSD, and base R to compute *t*-tests, correlations, and Wilcox tests.

We created graphs using GraphPad Prism (GraphPad, San Diego, CA, USA) or R using “ggplot2” (RStudio Inc.). Color scales were created using the “Viridis” package. Atlas images were adapted from Paxinos and Watson digital atlas (Paxinos and Watson, 2007) using Adobe Illustrator CC (Adobe Inc., San Jose, CA, USA). All other figure aspects were created in Adobe Illustrator CC.

## Results

### The LPO Sends GABA and Glutamate Projections to the VTA

We determined the relative proportions of GABA to glutamate neurons in the LPO projection to the VTA. To selectively express eYFP in LPO neurons that project to the VTA, we injected CAV-Cre in the VTA and EF1a-DIO-ChR2-eYFP in the LPO (Figure 1A). We then performed fluorescent *in situ* hybridization for GFP (which effectively probes for eYFP) to identify LPO neurons that project to the VTA, GAD1 (GAD-67) to identify GABA neurons, and VGLUT2 to identify glutamate neurons (Figures 1B,C). In LPO→VTA neurons, we found that ~60% expressed GAD1 alone, ~30% expressed VGLUT2 alone, ~3% coexpressed both GAD1 and VGLUT2, and ~7% did not express GAD1 nor VGLUT2 (Figure 1D). Furthermore, we found differences in the proportion of expression across the anterior to the posterior axis for both GABA and glutamate populations: expression of GAD1 was higher rostrally than caudally and expression of VGLUT2 was lower rostrally than caudally (Figure 1E). These results indicate that the LPO sends direct GABAergic and glutamatergic projections to the VTA and that GABA makes up a larger proportion of this projection in rostral portions of the LPO.

### The LPO and LPO→VTA Pathway Modulates VTA Subpopulations

To validate that ChR2 can stimulate LPO neurons, we measured the effect of optogenetic stimulation of LPO neurons in rats that received an intra-LPO injection of a viral vector encoding ChR2. We recorded the activity of LPO neurons under anesthesia while delivering local laser illumination (450 nm, 1–10 mW; Supplementary Figure S1A). Delivering single 10 ms light pulses at 0.2–0.5 Hz produced responses with low latency and low jitter (Supplementary Figures S1C,E); delivering 1 s trains of 5 ms light pulses at 20 and 40 Hz stimulated LPO neurons with high fidelity (Supplementary Figures S1D,F).

We measured the functional connectivity between the LPO and VTA in rats that received an intra-LPO injection of a viral vector encoding ChR2. We recorded the activity of VTA neurons under anesthesia while delivering laser illumination either to the LPO or to the VTA (to stimulate the LPO→VTA pathway).

Stimulation of the LPO had differential effects on VTA_GABA_ (Figure 2A) and VTA_Dopamine_ (Figure 2B) neurons (neuron type × time-bin interaction: *F*_(2,64)_ = 3.68, *P* = 0.031). In the case of VTA_GABA_ neurons, stimulation of the LPO produced a strong decrease in firing rate that returned to baseline after the stimulation ended (time-bin effect: *F*_(2,30)_ = 17.87, *P* < 0.001; HSD pre vs. pulse: *P* < 0.001; HSD pre vs. post: *P* = 0.13; Figure 2C). In the case of VTA_Dopamine_ neurons, stimulation of the LPO produced a mixture of effects that did not lead to a group effect (time-bin effect: *F*_(2,34)_ = 0.84, *P* = 0.44; Figure 2D). However, LPO stimulation led to an increase in firing in 4 out of 18 neurons, a decrease in firing in 9 out of 18 neurons, and no effects in 5 out of 18 neurons. Increases and decreases in VTA_Dopamine_ neuron activity were often observed within the same subject (data not shown). We found that the position of the cell within the VTA and the position of the optic fiber in the LPO did not correlate with the effect of stimulation of the LPO (Supplementary Figures S2A, S3).

Stimulation of the LPO→VTA pathway had differential effects on VTA_GABA_ and VTA_Dopamine_ neurons (cell type × time-bin interaction: *F*_(2,116)_ = 6.57, *P* = 0.0020). In the case of VTA_GABA_ neurons, stimulation of the LPO→VTA pathway produced a strong decrease in firing rate that returned to baseline after the stimulation ended (time-bin effect: *F*_(2,38)_ = 20.40, *P* < 0.001; HSD pre vs. pulse: *P* < 0.001; HSD pre vs. post: *P* = 0.27; Figure 2E). In the case of VTA_Dopamine_ neurons, stimulation of the LPO produced a mixture of effects that did not lead to a group effect (time-bin effect: *F*_(2,78)_ = 0.42, *P* = 0.66; Figure 2F). However, LPO stimulation led to an increase in firing in 8 out of 40 neurons, a decrease in firing in 13 out of 40 neurons, and no effects in 19 out of 40 neurons. Increases and decreases in VTA_Dopamine_ neuron activity were often observed within the same subject (data not shown). We found that the position of the cell within the VTA did not correlate with the effect of stimulation of the LPO→VTA pathway (Supplementary Figure S2B).

To further validate the effect of stimulating the LPO→VTA pathway, we used a combinatorial approach. Rats received an intra-VTA injection of the retrograde vector CAV-2 Cre and an intra-LPO injection of the viral vector encoding EF1a-DIO-ChR2-eYFP. This approach results in the selective expression of ChR2 in LPO neurons that project to the VTA (Junyent and Kremer, 2015). Stimulation of the cell bodies of the LPO→VTA pathway did not have differential effects on VTA_GABA_ and VTA_Dopamine_ neurons but trended in that direction (time-bin effect: *F*_(2,60)_ = 2.57, *P* = 0.085). In the case of VTA_GABA_ neurons, stimulation of the cell bodies of the LPO→VTA pathway produced a strong decrease in firing rate that returned to baseline after the stimulation ended (time-bin effect: *F*_(2,48)_ = 14.69, *P* < 0.001; HSD pre vs. pulse: *P* < 0.001; HSD pre vs. post: *P* = 0.62; Figure 2G). In the case of VTA_Dopamine_ neurons, stimulation of the LPO produced a mixture of effects that did not lead to a group effect (time-bin effect: *F*_(2,12)_ = 0.0027, *P* = 1.00; Figure 2H). However, stimulation of the LPO led to an increase in firing in two out of seven neurons, a decrease in firing in one out of seven neurons, and no effects in four out of seven neurons.

In a subset of VTA neurons, we were able to compare the effect of stimulating the LPO and stimulating the LPO→VTA pathway. We found that stimulating the LPO and LPO→VTA pathway had similar effects (paired *t*-test, GABA: *t*_(9)_ = 0.67, *P* = 0.52; DA: *t*_(14)_ = 1.73, *P* = 0.11; Figures 2I,J, left) and were correlated cell by cell (Figures 2I,J, right).

We also determined if stimulations of long and short duration produce similar effects in a subset of neurons (Supplementary Figure S4). We stimulated the LPO and LPO→VTA pathway for 1 and 60 s and recorded the change in firing in VTA neurons. In the case of VTA_GABA_ neurons, for both the LPO and LPO→VTA pathway, 1 and 60 s stimulation had similar effects (paired *t*-test, LPO: *t*_(5)_ = 0.55, *P* = 0.61; LPO→VTA: *t*_(5)_ = 0.81, *P* = 0.46). In the case of VTA_Dopamine_ neurons, stimulation of the LPO with 1 s stimulation produced smaller effects compared with 60 s stimulation (paired *t*-test, *t*_(10)_ = 3.12, LPO: *P* = 0.011), while stimulation of the LPO→VTA pathway with 1 and 60 s stimulation produced similar effects (paired *t*-test: *t*_(9)_ = 1.87, LPO→VTA, *P* = 0.095). Taken together, these data indicate that stimulating the LPO produces effects even with long stimulation (60 s).

### Optogenetic Stimulation of the LPO Supports Intracranial Self-stimulation Responding

#### Intracranial Self-stimulation (ICSS): Fixed Ratio Schedule

To determine if increases in neuronal activity within the LPO is reinforcing, we allowed rats to optogenetically self-stimulate the LPO during an ICSS procedure. We injected viral vectors encoding either mCherry or ChR2 into the LPO and implanted an optical fiber above the injection site (Figure 3A).

In the first cohort of rats (Cohort 1), rats were tested for ICSS over 6 days (Figures 3B,C). We used a fixed ratio schedule of 1: one nose-poke into the active hole triggered a 1 s train of intra-LPO laser illumination (40 Hz, 5 ms pulses, 15 mW, 450 nm) and a simultaneous light cue in the active hole. Nose pokes during illumination were recorded but did not trigger a subsequent illumination. To reduce skewness, response counts were transformed logarithmically (before transformation all response counts were increased by one to avoid undefined values resulting from the log of zero).

Optogenetic stimulation of the LPO supported ICSS, as indicated by differential discrimination for the active hole vs. the inactive hole between the mCherry and ChR2 groups (group × hole interaction: *F*_(1,14)_ = 51.57, *P* < 0.001). Furthermore, the ChR2 group had higher responding on the active hole compared with the mCherry group (group effect: *F*_(1,14)_ = 68.74, *P* < 0.001; Figure 3D).

To determine if response rates are sensitive to the duration of the stimulation per reward, after 6 days of ICSS with a reward duration of 1 s, we increased the reward duration to 10 s. Increasing the reward duration led to differential effects in the mCherry and ChR2 groups, where the ChR2 group decreased active hole responding and the mCherry group showed no change (group × reward duration, *F*_(1,14)_ = 17.79, *P* < 0.001, ChR2: HSD, *df* = 14, *P* < 0.001; mCherry: HSD, *df* = 14, *P* = 0.99). For both reward durations, the ChR2 group maintained higher active-hole response rates compared with the mCherry group (group effect, *F*_(1,14)_ = 146.56, *P* < 0.001; 1 s: HSD, *df* = 18.76, *P* < 0.001; 10 s: HSD, *df* = 18.76, *P* < 0.001; Figure 3E, left). Increasing the reward duration led to increased total stimulation duration in both groups, but with a greater increase in the mCherry group compared with ChR2 (reward duration effect, *F*_(1,14)_ = 380.89, *P* < 0.001; group × stimulation reward duration interaction, *F*_(1,14)_ = 21.38, *P* < 0.001; ChR2: HSD, *df* = 14, *P* < 0.001; mCherry: HSD, *df* = 14, *P* < 0.001; Figure 3E, right). This result stems from the fact that the mCherry group maintained response rates under both stimulation durations, which led to a linear increase in the total stimulation duration when the duration of the reward was increased. Even with the differential change in total stimulation duration, the ChR2 group earned a greater total stimulation duration for both reward durations than the mCherry group (group effect, *F*_(1,14)_ = 133.05, *P* < 0.001; 1 s: HSD, *df* = 17.60, *P* < 0.001; 10 s: HSD, *df* = 17.61, *P* < 0.001). These results indicate that stimulating the LPO is reinforcing and that rats flexibly adjust response rates in correspondence to reward duration; the ChR2 group reduced the number of responses to compensate for the increase in reward duration. ICSS was also conducted in Cohort 2 using 40 Hz and in Cohort 3 using 20 Hz stimulation. In all cohorts, stimulation of the LPO supported ICSS (Supplementary Figures S5B,C).

Fiber locations were similar across cohorts (Supplementary Figure S6) and we observed no correlation between the position of the optic fiber and the mean number of responses over the last 3 days of ICSS (Supplementary Figure S7). This indicates that any variability in behavior cannot be explained by variability in fiber placement.

#### ICSS: Progressive-Ratio Schedule

To determine the extent of the reinforcing properties of LPO stimulation and to determine the relative value of different stimulation durations, we tested the second cohort of rats (Cohort 2) with a progressive-ratio schedule. The ChR2 group was first trained with fixed-ratio 1 ICSS for 1 s stimulation over 5 days, as outlined above, and then tested over 12 days of progressive-ratio (Figure 3F). For all progressive-ratio days, the cost of the reward increased semi-logarithmically during the session (e.g., 1, 2, 4, 6, 9, etc.; Figure 3G). Every other day, the stimulation duration per reward was increased such that the rats were tested on two consecutive days for each stimulation duration (e.g., 1 s, 1 s, 3 s, 3 s, etc.; Figure 3F). A mean was calculated across days with the same stimulation duration. The purpose of progressively increasing stimulation duration was to determine if there was a duration at which LPO stimulation was no longer reinforcing which would manifest as a decrease in response rates.

The group of ChR2 rats acquired ICSS, as indicated by discrimination between the active hole and inactive hole (hole effect: *F*_(1,6)_ = 164.07, *P* < 0.001; Figure 3H), and rats continued to discriminate throughout the progressive-ratio test (hole effect: *F*_(1,6)_ = 165.93, *P* < 0.001). Increasing the stimulation duration per reward led to an increase in active hole responding (reward duration effect: *F*_(5,30)_ = 4.93, *P* = 0.0021; HSD, 1 s vs. 10 s *P* = 0.0019, 1 s vs. 60 s *P* = 0.0074; Figure 3I, left), and breakpoint (reward duration effect: *F*_(5,30)_ = 2.83, *P* = 0.031; Figure 3I, middle). Additionally, increasing the stimulation duration per reward led to a dramatic increase in the total stimulation duration received (reward duration effect: *F*_(5,30)_ = 975.20, *P* < 0.001; HSD, 1 s vs. all other durations *P* < 0.001; Figure 3I, right). For stimulation durations of 300 s, rats earned a mean total of 48.21 min of stimulation (SEM: 5.94 min) during the 6 h of testing. Critically, no stimulation duration led to a suppression in responding that was lower than responding for 1 s stimulation. These results were replicated in Cohort 1, even though Cohort 1 went through multiple pilot experiments in between ICSS testing at FR1 and using the progressive-ratio schedule (Supplementary Figure S5E). Altogether, the progressive-ratio experiment reveals that LPO stimulation is reinforcing under high ratio requirements even up to very long stimulation durations and there is no point at which stimulation transitions to being no longer reinforcing.

### Optogenetic Stimulation of the LPO Promotes Real-Time Place Aversion in the Majority of Rats

To determine the valence of stimulating the LPO with optogenetics, we measured the online valence of optogenetic stimulation with RTPT (Figure 4). We injected viral vectors encoding either mCherry or ChR2 into the LPO and implanted optical fibers above the injection site (Figure 4A). Rats were then subjected to RTPT (Figures 4B,C). In Cohort 2, laser illumination with 40 Hz, 3 s trains, 3 s ITI had different effects on the mCherry and ChR2 groups (group × day interaction: *P* < 0.001, *F*_(8,96)_ = 5.56, *P* < 0.001; Figures 4D,E). Importantly, real-time place aversion behavior was observed in the same subjects that exhibited ICSS responding (Supplementary Figures S8A–D for a representative rat). To determine if groups exhibited a difference in the number of times they crossed into the laser paired side, we examined the number of crossings and found that the mCherry and ChR2 groups crossed at similar rates (Supplementary Figure S9B; *F*_(1,12)_ = 3.08, *P* = 0.10).

This experiment was also conducted in Cohort 1 using 40 Hz continuous train illumination. Illumination did not have differential overall effects on the mCherry and ChR2 groups (group × day interaction: *F*_(8,104)_ = 1.59, *P* = 0.14; Figure 4F). However, there was a bidirectional effect on the RTPT score within the ChR2 group (Figure 4G). In this cohort, the ChR2 group made more crossings compared with the mCherry group (*F*_(1,13)_ = 20.02, *P* < 0.001; Supplementary Figure S9A). Altogether, the results in Cohort 1 largely replicated the effects in Cohort 2 except rats crossed more frequently than Cohort 2.

Finally, we conducted RTPT in the third cohort of rats (Cohort 3) using 20 Hz continuous train illumination and biased assignment where laser pairing was assigned to the side least preferred during the preference test, to enhance the likelihood of detecting place preference. Despite biased assignment, rats still did not prefer the laser paired side. Illumination had a trend towards producing differential effects on the GFP and ChR2 groups (group × day interaction: *F*_(6,60)_ = 2.05, *P* = 0.072; Supplementary Figures S10A,B). Finally, the GFP and ChR2 groups made a similar number of crossings (*F*_(1,10)_ = 0.18, *P* = 0.67; Supplementary Figure S9C). Results from this cohort indicate that optogenetic stimulation of the LPO with low frequencies produces a trend towards aversion.

Fiber locations were similar across cohorts (Supplementary Figure S6) and we observed no correlation between the position of the optic fiber and the RTPT score (Supplementary Figure S11). This indicates that any variability in behavior cannot be explained by variability in fiber placement.

### Optogenetic Stimulation of the LPO→VTA Pathway Replicates the Effects of Stimulation of the LPO

To determine if the effects of optogenetic stimulation of the LPO is mediated in part by the LPO→VTA pathway, we injected viral vectors encoding either mCherry or ChR2 into the LPO and implanted optical fibers above the injection site in the LPO and above the VTA (Figure 5A, fiber placements shown in Supplementary Figure S12). Rats were then subjected to RTPT (Figure 5B), during which laser illumination of the LPO→VTA pathway had differential effects on the mCherry and ChR2 groups (group × day interaction: *F*_(8,96)_ = 4.34, *P* < 0.001; Figures 5C,D). The mCherry and ChR2 groups made similar numbers of crossings (*F*_(1,12)_ = 0.0014, *P* = 0.97; Supplementary Figure S9E). These results largely replicated RTPT results obtained with stimulation of LPO cell bodies as outlined above.

In addition to measuring valence with RTPT, we measured the reinforcing qualities of optogenetic stimulation of the LPO and the LPO→VTA pathway with a modified version of the ICSS described above, where rats received access to the illumination of the LPO→VTA pathway for 9 days, followed by illumination of LPO cell bodies for 3 days, and finally illumination of the LPO→VTA pathway for 3 additional days. Throughout ICSS, the ChR2 group acquired ICSS responding to a greater degree compared with the mCherry group, as indicated by differential discrimination between the active hole and inactive hole for the mCherry and ChR2 groups (group × hole interaction: *F*_(1,12)_ = 15.21, *P* = 0.0021; Figure 5E, left). To determine if there is a difference in reinforcement between stimulation of LPO cell bodies and stimulation of the LPO→VTA pathway, we took the mean responding over the last 3 days of initial illumination of the LPO→VTA pathway, the 3 days of illumination of LPO cell bodies, and the 3 days of illumination of the LPO→VTA pathway following illumination of LPO cell bodies. Across illumination regions, the mCherry and ChR2 groups differentially responded on the active hole and inactive hole (group × hole interaction: *F*_(1,12)_ = 22.42, *P* < 0.001). Over all three epochs, the ChR2 group responded more often on the active hole than the mCherry group but showed no difference in responding on the inactive hole (HSD, active hole: *P* < 0.001, inactive hole: *P* > 0.05 for all epochs; Figure 5E, right). Relative to the LPO→VTA pathway, stimulating LPO cell bodies led to higher rates of responding (group × hole × fiber interaction: *F*_(2,24)_ = 6.30, *P* = 0.0063; HSD, active hole: VTA-LPO vs. LPO, *P* < 0.01 for both comparisons; data not shown), and this effect was reduced after log transformation of the data (group × hole × fiber interaction: *F*_(2,24)_ = 2.74, *P* = 0.085; HSD, active hole: VTA-LPO vs. LPO, *P* > 0.05 for both comparisons; Figure 5E, right). Together, the RTPT and ICSS data indicate that stimulation of the LPO→VTA pathway is both reinforcing and aversive, and this mirrors the effect of stimulating LPO cell bodies.

### ICSS and RTPT Measures of Reward Are Correlated

To understand the apparent contradiction stemming from the ICSS (reinforcement) and RTPT (aversion) results outlined above, we analyzed the relationship between ICSS and RTPT behavior in rats that were tested with both procedures.

We first chose to analyze ChR2 rats of Cohort 2, which displayed the strongest real-time place aversion with 3 s trains, 3 s ITI stimulation but also displayed both fixed-ratio and progressive-ratio responding. We analyzed the relationship between RTPT and fixed-ratio 1 ICSS because both tasks enable rats to administer as much stimulation as desired. To compare these two assays, we compared days 2–4 of both assays (ICSS: days 2–4 of FR1; RTPT: days 2–4 of initial pairing) over the first 20 min of both procedures (Figure 6A). There was a strong positive correlation between the total stimulation duration received in RTPT and fixed-ratio 1 ICSS (*r* = 0.91, *P* < 0.001, data not shown), with rats receiving more stimulation during RTPT compared with ICSS (Wilcoxon, *W* = 26, *P* = 0.031; Figure 6B).

The relationship between ICSS and RTPT was similar across experiments. Combining data across Cohorts 1, 2, and 3, and the LPO→VTA pathway stimulation group, rats received more stimulation in RTPT compared with ICSS over the same 20-min time period (Wilcoxon, *W* = 350, *P* < 0.001; Supplementary Figure S13A). Furthermore, the total stimulation duration received within these two assays was positively correlated (*r* = 0.43 *P* = 0.029; Supplementary Figure S13B), even when normalizing within each experiment (*r* = 0.45, *P* = 0.020; Supplementary Figure S13C); these results indicate that despite the large difference in total stimulation duration, the assays are measuring a related effect.

### Stimulation-Intervals Contribute More to RTPT Results Than Inter-Stimulation-Intervals

After finding that rats received more optogenetic stimulation in the RTPT procedure than the ICSS procedure despite real-time place aversion, we next analyzed specific components of the behavior to determine what underlies the ultimate RTPT score. We broke down the task into two independent components: (1) the stimulation-interval (SI; duration of each stimulation period); and (2) the inter-stimulation-interval (ISI; duration of periods in between each stimulation period). In the case of ICSS, the rats can only control the ISI, as the SI is fixed at 1 s by the experimenter (Figure 6C, left). In the case of RTPT, the rats can control both the ISI and SI, both of which could independently underlie the final RTPT score (Figure 6C, right). For example, the variance in RTPT scores could be the result of consistent ISI with variable SI, where high stimulation-intervals would lead to high RTPT scores or could be the result of consistent SI with variable ISI, where low ISI would lead to higher RTPT scores.

During RTPT, there was a limited relationship between the ISI and RTPT score, suggested by the cumulative distribution functions of ISI for each rat (Figure 6D). This was further demonstrated by a poor correlation between the median ISI and RTPT scores (Figure 6E). These results indicate that there is a limited relationship between the ISI (the rates at which rats re-enter the stimulation paired side), and the RTPT score. On the other hand, there was a strong relationship between the SI and RTPT score, suggested by the cumulative distribution functions of SI for each rat (Figure 6F) and a strong positive correlation between the median SI and RTPT score (Figure 6G).

The relationships between the RTPT score and the ISI and SI was also seen when combining rats across experiments (Supplementary Figure S14). We combined rats from Cohort 1, 2, and 3, along with the LPO→VTA pathway experiment, and observed similar relationships to the analysis of Cohort 2 alone. Compared with the ISI, the SI correlated more strongly with the RTPT score (Supplementary Figure S14).

Altogether these results indicate that optogenetic stimulation of the LPO produces the ultimate RTPT scores primarily through differences in preferred SI and not through preferred ISI. These results raise the possibility that stimulating the LPO with optogenetics is reinforcing despite real-time place aversion, which would manifest as continued entry into the paired compartment despite the lack of preference for the environment after entering.

### Optogenetic Stimulation of the LPO Reduces Foot-Shock Avoidance

To determine if optogenetic stimulation of the LPO is reinforcing in the RTPT procedure, we injected viral vectors encoding either mCherry or ChR2 into the LPO and implanted optical fibers above the injection site (Figure 7A, fiber placements shown in Supplementary Figure S15). Rats were then subjected to RTPT with and without electricity delivered *via* the floor of the laser-paired side. In this procedure, rats were run through RTPT in an apparatus where one side was covered in Plexiglas (safe side) and the other consisted of metal bars (electrified side). Note that there was a strong baseline preference for the metal bars over Plexiglass in both groups that was not seen in the standard RTPT assay, likely because there was a salient difference between the flooring. Throughout the entire procedure, the side of the apparatus with metal bars was paired with illumination (40 Hz, 3 s trains, 3 s ITI). Following 4 days of pairing, the metal bars of the floor on the illumination paired side were electrified according to the schedule outlined in Figures 7B,C. This side of the apparatus will be referred to as the dual-paired side. Electrifying the floor of the dual-paired side led to a decreased amount of time spent in the dual-paired side (day effect: *F*_(17,255)_ = 30.34, *P* < 0.001; Figure 7D). However, this occurred to a greater extent in the mCherry group than the ChR2 group (day × group interaction: *F*_(17,255)_ = 5.55, *P* < 0.001; Figure 7D) and was present throughout the testing session (Supplementary Figures S16A–C). To determine if the ChR2 group was willing to endure the electricity when entering the dual-paired side to receive stimulation, we analyzed the number of crossings that led to a minimum dual-paired interval of 3 s; this ensures that the rat will receive stimulation during each visit due to an illumination pattern consisting of 3 s trains and 3 s ITI. Electrifying the floor led to fewer dual-paired intervals greater than 3 s (day effect: *F*_(17,255)_ = 26.71, *P* < 0.001; Figure 7E). However, this occurred to a greater extent in the mCherry group compared with the ChR2 group (day × group interaction: *F*_(17,255)_ = 4.06, *P* < 0.001; Figure 7E). The greater amount of time spent in the dual-paired compartment and greater number of dual-paired intervals greater than 3 s relative to control animals indicate that stimulation of the LPO is reinforcing, even if it does not drive increased time spent in the paired compartment during standard RTPT procedures.

### Optogenetic Inhibition of the LPO Does Not Promote ICSS Responding Nor Real-Time Place Testing Behavior

To validate that NpHR can inhibit LPO neurons, we measured the effect of optogenetic inhibition of LPO neurons in rats that received an intra-LPO injection of a viral vector encoding NpHR. We recorded the activity of LPO neurons under anesthesia while delivering laser illumination (520 nm, 2 mW) into the LPO (Supplementary Figures S17A,B). Delivering 50 ms light pulses at 0.2 Hz produced low, rapid inhibition of activity that quickly recovered; delivering 1 s, 10 s, and 60 s light pulses produced total and sustained inhibition of activity without producing rebound excitation (Supplementary Figures S17C–E).

**Figure 8:**
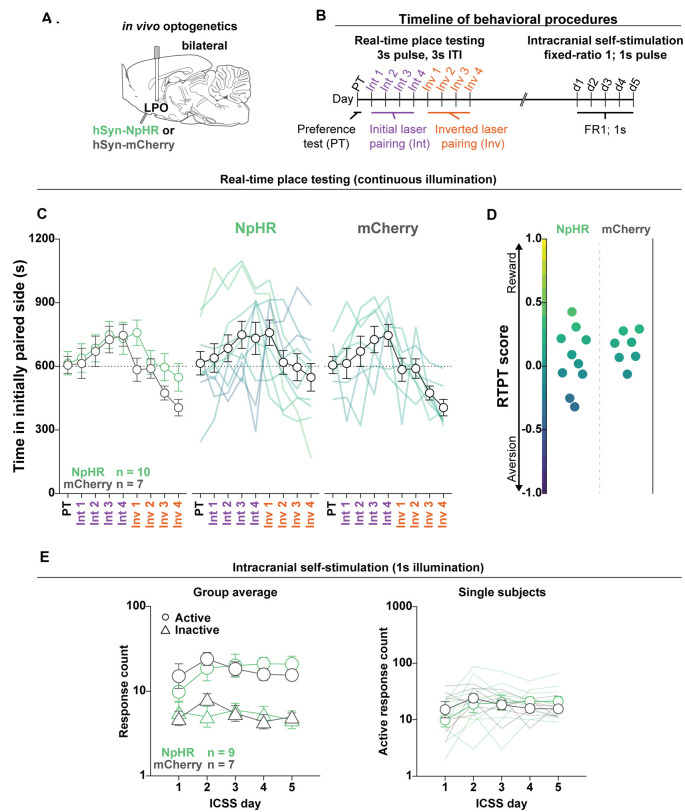
Optogenetic inhibition of the LPO does not support ICSS or drive real-time place preference. **(A)** *In vivo* optogenetics setup: we injected either hSyn-HR (NpHR) or hSyn-mCherry (mCherry) bilaterally in the LPO and implanted optic fibers overlying the injection sites. **(B)** Timeline for RTPT and ICSS (procedure details can be found in legends for Figures 3, 4). **(C)** The mean time in the initially paired side across days of RTPT in NpHR (green) and mCherry (gray) groups; single rats are color-coded based on their RTPT score. The NpHR and the mCherry groups showed preference but did not show different behavior across days of RTPT (day effect: *F*_(8,120)_ = 4.80, *P* < 0.001, group × day interaction: *F*_(8,120)_ = 0.74, *P* = 0.65). **(D)** RTPT scores for rats in the NpHR and mCherry groups. **(E)** Self-administration behavior during ICSS at a fixed-ratio 1 for 1 s illumination. Left: the NpHR and the mCherry groups did not show different discrimination between the active hole (Active, circles) and inactive hole (Inactive, triangles; group × hole interaction: *F*_(1,14)_ = 0.011, *P* = 0.92); right: the NpHR group did not make more or less active hole responses than the mCherry group throughout the ICSS procedure (group effect: *F*_(1,14)_ < 0.001, *P* = 1.00). Active hole and inactive hole responses are shown on a log scale. In **(C,E)**, faded lines depict values from individual rats; points and error bars depict mean and SEM, respectively.

To determine if the LPO provides a tonic regulation of valence, we injected viral vectors encoding either mCherry or NpHR and implanted optical fibers above the injection site (Figure 8A, fiber placements shown in Supplementary Figure S18). Rats were then subjected to RTPT and ICSS, sequentially (Figure 8B). Interestingly, laser illumination in the LPO in both mCherry and NpHR groups drove a slight preference for laser illumination (day effect: *F*_(8,120)_ = 4.80, *P* < 0.001; Figure 8C). However, laser illumination did not drive differential RTPT behavior across groups (group × day interaction: *F*_(8,120)_ = 0.74, *P* = 0.65; Figures 8C,D). Laser illumination also did not drive differences in crossings between the mCherry and NpHR groups (Supplementary Figure S9D; *F*_(1,15)_ = 0.20, *P* = 0.66). In the ICSS procedure, the mCherry and NpHR groups did not exhibit differential discrimination between the active and inactive holes (group × hole interaction: *F*_(1,14)_ = 0.011, *P* = 0.92; Figure 8E, left), or differences in active hole responses (group effect: *F*_(1,14)_ < 0.001, *P* = 1.00; Figure 8E, right), indicating that inhibition of the LPO was neither reinforcing nor aversive. Together, the RTPT and ICSS results indicate that the LPO does not provide tonic regulation of valence or reinforcement.

### The LPO Signals to Aversive Conditioning, but Not Rewarding Conditioning

**Figure 9:**
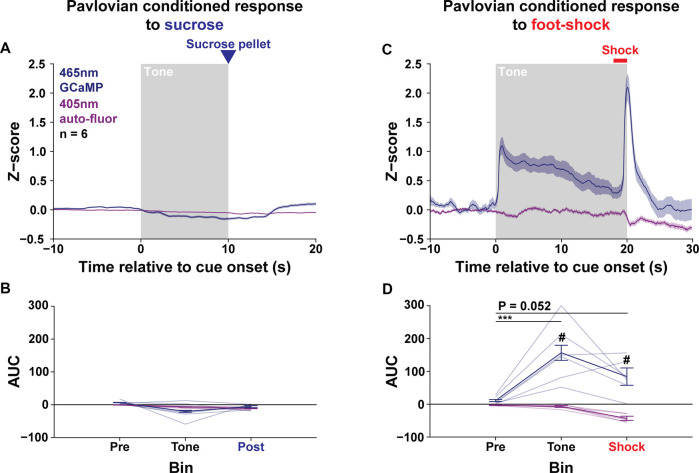
The LPO signals to aversive conditioning, but not rewarding conditioning. **(A)** Mean fiber photometry signals in the LPO across days 25–27 of Pavlovian conditioning for sucrose. The predictive tone and sucrose delivery did not change signals in either channel (6 subjects × 90 trials). **(B)** The area under the curve (AUC) for data shown in **(A)**. The LPO does not show changes in activity during the tone and post sucrose periods. **(C)** Mean fiber photometry signals on day 3 of Pavlovian conditioning for foot-shock. The predictive tone and electric foot-shock (EFS) led to an increase in *z*-score within the GCaMP channel (blue) but not the autofluorescent channel (purple; 6 subjects × 5 trials). **(D)** The AUC for *z*-scores shown in **(C)**. The LPO shows enhanced activity during the tone and shock periods (HSD, vs. Pre bin, ***P < 0.01; vs. 405 auto-fluorescent channel, ^#^*P* < 0.05). In **(A,C)**, the thick link depicts group mean and shaded ribbon depicts SEM; colors indicate the recording channel (465 nm GCaMP channel: blue; 405 nm auto-fluorescent channel: purple). In **(B,D)**, faded lines depict subject mean AUC and dark lines depict group mean and SEM; the bin size for all AUC data was 10 s in duration, the pre bin started 10 s before tone onset (−10 s), the tone bin began at tone onset (0 s), the shock bin started at shock onset (18 s), and the post sucrose bin began at sucrose delivery (10 s).

Given the complex effects of optogenetic stimulation of the LPO outlined above, we measured the natural activity of the LPO during rewarding and aversive events using fiber photometry. To this end, we recorded the LPO during Pavlovian conditioning for sucrose and electric foot-shock. To record calcium signals with fiber photometry, we injected a viral vector encoding a calcium indicator, GCaMP6f, into the LPO and implanted an optical fiber above the LPO. To measure selective increases in calcium activity, we recorded a 465 nm GCaMP channel and a 405 nm autofluorescent channel, and then looked for differential changes in these channels during behavior (Supplementary Figures S19A–E); selective increases in the 465 nm GCaMP channel will be referred to as calcium signals from this point forward. Before Pavlovian conditioning for sucrose, rats received magazine training and were pre-exposed to a 10 s tone cue (76 dB, 3 kHz) that would later be paired with sucrose delivery (Supplementary Figure S20B). Throughout magazine training, rats acquired an association between the sound of the pellet delivery and the presence of sucrose pellets in the food port, as indicated by a progressively decreased latency from pellet delivery to port entry over training (day effect: *F*_(5,25)_ = 17.73, *P* < 0.001; Supplementary Figure S21A). For Pavlovian conditioning, rats were placed in a chamber where they received 30 pairings of a 10 s tone and sucrose pellet delivery (Supplementary Figure S20E). Throughout Pavlovian conditioning, rats acquired an association between the sound of the tone and the presence of sucrose pellets in the food port, as indicated by a progressively increased number of port entries during the tone compared with an equal length of time before the tone (supplemental; period × day interaction: *F*_(14,70)_ = 2.68, *P* = 0.0034; Supplementary Figures S21B,C). After 12 days of Pavlovian conditioning, we recorded the activity of the LPO for 3 additional days of conditioning. In trained animals, the LPO did not exhibit calcium signals in response to the tone or sucrose delivery (channel × time interaction: *F*_(2,10)_ = 2.79, *P* = 0.11; Figures 9A,B). This lack of time-locked LPO calcium signals was observed despite the presence of non-time-locked spontaneous transients (data not shown). Small reductions in both the GCaMP and autofluorescent channels were observed during the tone period of late Pavlovian conditioning (Supplementary Figure S22A) which mirrored the time course of head entries into the food port throughout training (Supplementary Figure S21D). However, there were no changes in time-locked LPO calcium signals throughout training (training stage × channel × time interaction: *F*_(4,20)_ = 2.15, *P* = 0.11; Supplementary Figures S22A,B). To test if the LPO may signal a reward-prediction error, we ran rats through 3 more days of Pavlovian conditioning for sucrose where 10% of trials occurred without pellet delivery (omission) and 10% of trials occurred without the tone (unexpected). During this procedure, LPO calcium signals did not signal differentially based on expectancy during the post sucrose delivery bin but did show a trend towards that effect (channel × expectancy interaction: *F*_(2,10)_ = 3.89, *P* = 0.056; Supplementary Figures S22C,D). Importantly, there were small decreases in both channels that were time-locked to head entries into the food port which leads us to believe this trend is the consequence of a movement artifact and not LPO calcium activity. Together, these results indicate that the LPO does not signal to sucrose or sucrose predicting cues.

Following Pavlovian conditioning for sucrose, rats were trained in Pavlovian conditioning for foot-shock over 3 days in a different apparatus from the one used for Pavlovian conditioning for sucrose. Fear conditioning consisted of five pairings of a 20 s tone (74 dB, 5 kHz) with a co-terminating foot-shock (2 s, 0.7 mA; Supplementary Figure S20G). We recorded the activity of the LPO for all 3 days of conditioning. In trained animals (day 3), LPO calcium signals increased in response to foot-shock predictive tones and trended towards an increase in calcium signals to foot-shock (channel × time interaction: *F*_(2,10)_ = 11.06, *P* = 0.0029; HSD GCaMP channel pre vs. tone: *P* < 0.001; HSD GCaMP channel pre vs. shock: *P* = 0.052; Figures 9C,D). When all 3 days of training were averaged, LPO calcium signals increased in response to both foot-shock predictive tones and foot-shock (channel × time interaction: *F*_(2,10)_ = 11.06, *P* = 0.0012; HSD GCaMP channel pre vs. tone: *P* < 0.001; HSD GCaMP channel pre vs. shock: *P* < 0.001; data not shown). The LPO calcium signal response to the tone developed throughout training, indicated by an increase in calcium response to the tone over days (channel × time × day interaction: *F*_(4,20)_ = 4.13, *P* = 0.013, HSD GCaMP channel tone day 1 vs. day 3: *P* < 0.001; Supplementary Figure S23C). These results indicate that the LPO signals in response to aversive events and to cues that predict the aversive events.

## Discussion

We found that the stimulation of the LPO produces primarily inhibition on VTA_GABA_ neurons and mixed effects on VTA_Dopamine_ neurons. We also found that stimulation of LPO cell bodies, regardless of where they project, had similar effects as stimulation of the LPO→VTA pathway, suggesting that the LPO has the same downstream modulation of VTA neurons through both a direct projection and intermediary structures. Our results also demonstrate that stimulation of LPO cell bodies, as well as the LPO→VTA pathway, supports ICSS responding but also produces aversion in the majority of rats within the RTPT assay. Importantly, we observed both ICSS responding and aversion during RTPT within the same rats. Despite the apparent contradiction, the behavior across these two assays was correlated, which suggests that the two tests are measuring related behaviors. We analyzed the underpinnings of RTPT scores by assessing the correlation between the RTPT scores and the stimulation-intervals or ISIs and found that the stimulation-intervals correlated more strongly with the ultimate RTPT score than ISIs did. We hypothesized that stimulation of the LPO is reinforcing despite the lack of preference within RTPT and tested this by pairing the stimulation-paired side with an aversive stimulus (electricity). We found that compared with controls, optogenetic stimulation of the LPO led to a greater amount of time spent in this dual-paired side and a greater number of crossings into this dual-paired side. Finally, we recorded the activity of the LPO during rewarding and aversive Pavlovian conditioning and found that the LPO increases activity in response to foot-shock and related predictive cues, but not sucrose and sucrose predicting cues. Altogether, our results indicate that the LPO has a complex functional regulation of VTA neurons, generates a complex reward phenotype, and signals in response to aversive events.

We found that the LPO projection to the VTA is made of a higher proportion of GABA neurons compared with that of glutamate. This result contrasts with previous findings that the LPO projection to the VTA contains similar levels of GABA and glutamate neurons (Kalló et al., 2015). Relative to Kalló et al. (2015), we found similar proportions of LPO→VTA glutamate neurons but substantially more LPO→VTA GABA neurons. There are several possible explanations for this discrepancy. First, it could be due to the *in situ* probe for GABA. We used a probe for GAD-67, whereas Kalló et al. (2015) used a probe for GAD-65, and these mRNAs have differential expression (Esclapez et al., 1993, 1994). Another explanation could be the anterior-posterior definition of the LPO. We found that there is a difference in the proportion of LPO→VTA neurons that are GABAergic and glutamatergic across the anterior-posterior extent of the LPO. Anterior to posterior there is a decrease in the proportion of GABA neurons and an increase in the proportion of glutamate neurons. Therefore, the difference between our work and Kalló et al. (2015) could be the anterior-posterior position of slices used. A final explanation could be the method used to label LPO→VTA neurons. We used a combinatorial viral approach while Kalló et al. (2015) used the Cholera toxin B subunit. These methods could potentially lead to differences in labeling for glutamate and GABA neurons as CAV-2 has been shown to infect subsets of neurons in other brain systems (Li et al., 2018). Regardless of the source of the discrepancy in proportions, our results support a mixed GABAergic and glutamatergic projection from the LPO to the VTA.

We determined that this mixed GABAergic and glutamatergic LPO→VTA projection primarily inhibits VTA_GABA_ neurons and produces mixed excitation and inhibition of VTA_Dopamine_ neurons. This is in contrast with our previous finding (Gordon-Fennell et al., 2020) that stimulation of the LPO with bicuculline leads to uniform inhibition of VTA_GABA_ neurons and uniform excitation of VTA_Dopamine_ neurons. One possible explanation is that short term optogenetic stimulation is producing differential downstream effects when compared with longer-term stimulation, produced pharmacologically. We tested this by recording VTA neurons during short term (1 s) and long term (60 s) stimulation. We found that long term stimulation led to sustained effects on VTA_GABA_ neurons but led to reduced, but not opposite, effects in VTA_Dopamine_ neurons. This leaves open the possibility that bicuculline stimulation and optogenetic stimulation are stimulating different populations of neurons. Bicuculline produces stimulation by disinhibiting neurons by antagonizing the GABA-A receptor, therefore it may be biased towards stimulating neurons that are under tonic inhibition, which could be a different population of neurons than ones that are excited by ChR2. No matter what the underlying difference in the effect on VTA_Dopamine_ neurons is, the results presented here and in our previous publication indicate that the LPO is functionally connected to VTA neurons.

The LPO’s ability to differentially modulate VTA_Dopamine_ and VTA_GABA_ populations mirror the functional connectivity of the lateral hypothalamus (Nieh et al., 2016). The lateral hypothalamus can regulate the activity of dopamine neurons through GABAergic interneurons of the VTA. The GABA projection from the lateral hypothalamus to the VTA inhibits VTA_GABA_ neurons and enhances dopamine release in the nucleus accumbens while the glutamate projection excites VTA_GABA_ neurons and reduces dopamine release in the nucleus accumbens (Nieh et al., 2016). Our results could be the product of a similar form of connectivity. However, the mixed effects on the activity of VTA_Dopamine_ neurons and the consistent effect on VTA_GABA_ neurons suggest that the LPO must make direct functional connections with VTA_Dopamine_ neurons; if the LPO exclusively regulated VTA_Dopamine_ through VTA_GABA_, then we should have observed the only excitation of VTA_Dopamine_, as we only observed inhibition of VTA_GABA_. Further monosynaptic electrophysiology experiments and slice electrophysiology will be necessary to determine the precise circuit underlying the functional connectivity between the LPO and VTA.

In a subset of neurons, we were able to record the response to both stimulations of the LPO and the LPO→VTA pathway. We found that both manipulations led to similar results, indicating that stimulation of the LPO with all projections intact and stimulation of the LPO→VTA pathway had the same downstream effect on the VTA. We further verified the effects of LPO→VTA pathway stimulation using a combinatorial viral approach which selectively manipulates LPO neurons that project to the VTA. Overall, we observed similar results across LPO, LPO→VTA pathway, and LPO cell body stimulation of the LPO→VTA pathway (combinatorial viral approach). These findings suggest that the LPO sends redundant projections to intermediary structures, possibly the lateral habenula and rostromedial tegmental nucleus (Yetnikoff et al., 2015), that produce the same net effect in the VTA. Alternatively, when stimulating the LPO, the LPO→VTA pathway may short-circuit differential effects carried out by intermediary structures. The similarity in effects observed after stimulating the LPO and LPO→VTA pathway was also replicated in our behavioral assays. Together, these results indicate that the LPO and LPO→VTA pathway modulate the activity of VTA subpopulations.

We observed that optogenetic stimulation of the LPO was reinforcing with both short and long stimulation durations. Previous research demonstrated that stimulating the LPO with electricity supports ICSS (Fouriezos et al., 1987). However, it was not clear if LPO cell bodies or fibers of passage supported ICSS behavior because electrical stimulation of the LPO may be contaminated by stimulation of the medial forebrain bundle that passes through the LPO; thus, we are the first to demonstrate that neurons of the LPO themselves support ICSS responding. Furthermore, we found that animals dynamically shifted their behavior with changes in reward duration: increases in reward duration lead to a decreased responding under a fixed-ratio 1 schedule but led to increased responding under a progressive-ratio schedule. These results mirror what is seen with drugs of abuse, where increasing the reward size can simultaneously lead to decreases in fixed-ratio responding and increases in progressive-ratio responding (Arnold and Roberts, 1997). Interestingly, we did not observe a drop in progressive-ratio responding with stimulations up to 300 s in duration. This indicates that such long stimulations were not aversive to the rats. One possible caveat with the progressive-ratio result is that we tested subjects through ascending stimulation durations rather than using a Latin squared design. This was done by design, to determine if there was a transition from reward to aversion as the duration was increased, which was not observed. However, the increase in responding for longer stimulation durations could stem from changes throughout operant conditioning independent of changes in reward value. Indeed, rats received significantly longer total stimulation durations during progressive-ratio for 1 s at the end of training then they received during 1 s at the start of training. Even so, our results with fixed-ratio and progressive-ratio indicate that stimulation of the LPO is reinforcing across stimulation durations up to 300 s long.

We also found that stimulating the LPO is more reinforcing than stimulating the LPO→VTA pathway demonstrated by the same rats increasing responding when stimulation was switched from the LPO→VTA pathway to LPO cell bodies. A possible reason for lower stimulation rates in the LPO→VTA pathway compared with the LPO is that we are not simulating as many LPO→VTA terminals with optic fibers in the VTA as we do with optic fibers in the LPO. Alternatively, the LPO could produce ICSS behavior through different pathways that don’t rely on VTA_Dopamine_ activity (Britt et al., 2012). Our results are also the first to demonstrate that stimulation of the LPO→VTA pathway supports ICSS, which contradicts previous findings (Gigante et al., 2016). This could stem from the viral vector used for stimulation. Gigante et al. (2016) used a CaMKII promotor, while we used an hSyn promoter. These may lead to differential targeting that could produce differential downstream regulation of VTA_Dopamine_ neurons. Another more likely possibility could be the stimulation frequency used: Gigante et al. (2016) used 20 Hz stimulation while we used 40 Hz stimulation. With the stimulation of LPO cell bodies, we found that 20 Hz stimulation produced lower ICSS responses than 40 Hz stimulation (Supplementary Figure S5). Given that the LPO→VTA pathway stimulation with 40 Hz was lower than LPO cell body stimulation at 40 Hz, it stands to reason that LPO→VTA pathway stimulation with 20 Hz could be below the necessary stimulation for ICSS reinforcement.

We found that stimulating the LPO and LPO→VTA pathway produced bidirectional effects in RTPT but primarily produced aversion. Overall, stimulating the LPO and LPO→VTA pathway, on average, produced a reduction in time spent in the optically paired side without consistently changing the number of crossings. However, at the single-subject level, stimulation of the LPO and LPO→VTA pathway produced mixed effects, where the majority of rats showed clear aversion and a minority showed a clear preference. The mixture of valence produced by stimulating the LPO and LPO→VTA pathway in the RTPT assay could be due to the differential functional connectivity of the LPO across rats. Previous research has demonstrated that stimulation of the LPO→lateral habenula glutamate pathway produces real-time place aversion, while stimulation of the GABA pathway produces real-time place preference (Barker et al., 2017). The capacity of the LPO to mediate opposite effects on valence depending on whether glutamate or GABA neurons are stimulated grants the possibility that differences in the balance of connectivity of glutamate and GABA neurons across rats can explain differences in valence. The difference in valence we observed is unlikely to stem from regional differences in stimulation, as the location of the probe did not correlate with RTPT effects. While the underlying mechanism explaining the bidirectional effects within the RTPT assay is unclear, we have demonstrated that stimulation of the LPO is aversive in the majority of rats.

How can stimulation be reinforcing within ICSS but aversive in RTPT? One possibility is that the behavior underlying RTPT is more nuanced than previously thought. The standard metric used to determine if a neuronal manipulation is rewarding or aversive in RTPT is the amount of time spent on the optically paired side. However, our results indicate that time spent in the paired side in the RTPT task may not be sufficient to determine if a neuronal manipulation is rewarding or aversive. Many researchers have found that different neuronal manipulations contribute to RTPT effects but not ICSS effects, or vice versa. To our knowledge, only two published articles found aversion in RTPT and mild reinforcement with ICSS with the same brain system (Yoo et al., 2016; Zell et al., 2020). In these articles, the authors found that stimulating glutamate neurons in the VTA supports mild ICSS behavior but leads to a reduction in time spent in the optically paired side in the RTPT task. However, they found large increases in the number of crossings made into the optically paired side and a decrease in stimulation durations, which was interpreted as mice displaying a self-stimulation behavior for preferred short stimulations. In our study, we also observed ICSS and aversion in RTPT, but we did not observe consistent increases in crossings into the stimulation-paired side, as we only observed increased crossings in one group of rats (Cohort 1). An explanation for observing both aversions in RTPT and responding in ICSS is that stimulation is reinforcing despite not producing positive valence. This hypothesis is supported by our finding that the duration of stimulation intervals, but not ISIs correlates with the RTPT behavior (RTPT score): rats showed similar re-entry latencies (poor correlation of ISI with the RTPT score) but lingered in the stimulation side for different durations (strong positive correlation of stimulation-interval with the RTPT score). Furthermore, in the RTPT experiment where we used dual-pairing of both laser illumination and electricity, rats continued to enter the dual-paired side despite the adversity produced by the electricity. If stimulating the LPO is reinforcing despite not being rewarding, then an aversive RTPT score could be the result of rats being continually reinforced to enter the paired side but exiting once stimulation transitioned to being aversive. One remaining complexity is the lack of aversion with long-duration stimulation within the progressive-ratio experiment. However, in this assay, the stimulation duration is set by the experimenter, not the subject, which could produce different behavior.

We found that the LPO does not provide tonic regulation of valence or reinforcement, as bilateral inhibition of the LPO with NpHR produced no effects in both RTPT and ICSS tests. This may indicate that the LPO’s regulation of valence and reinforcement is evoked under specific contexts instead of being a consistent baseline regulator. Interestingly, in this experiment we observed a slight preference in RTPT for both the NpHR and control groups; this preference was not consistently observed in the ChR2 experiments. This could be due to effects stemming from continuous light illumination that was employed in NpHR experiments. Recent research indicates that continuous light pulses can modulate the activity of striatal neurons (Owen et al., 2019). Regardless of the baseline effects stemming from the general experimental procedure, inhibition of the LPO had no further effect.

Our photometry recording experiments revealed that the LPO had time-locked population signals to aversive events but not rewarding ones. We found that the LPO increases activity in response to foot-shock and that the LPO increases in activity in response to a tone which predicts foot-shock. On the other hand, the LPO showed no meaningful time-locked effects in response to sucrose or sucrose predictive cues. These results largely replicate results obtained from recording the calcium activity of the LPO to the lateral habenula pathway (Barker et al., 2017) which found that the LPO glutamate and GABA projection to the lateral habenula was excited by aversive events and related predictive cues but not rewarding events and related predictive cues. The lack of response to rewarding events or their predictive cues was not due to a lack of conditioning because rats showed behavioral conditioning. We observed small changes in fluorescence during the Pavlovian conditioning task for sucrose, however, these reductions were observed in both the 465 nm GCaMP channel and autofluorescent channel and largely mirrored head entry behavior. This leads us to believe that the reductions were most likely due to movement artifacts rather than being real differences in population activity within the LPO. Together, the Pavlovian conditioning results indicate that the LPO may be involved in responses to aversive events rather than rewarding events. Given the effects on reinforcement, it could be the case that activity in the LPO could reinforce behaviors necessary to avoid or escape aversive events. Our previous findings (Gordon-Fennell et al., 2020) demonstrated that both inhibition and stimulation of the LPO using pharmacology during punishment blocks the ability of punishment to drive lasting reductions in responding. Our current results provide evidence that the LPO signals during aversive events, which suggests that it could naturally influence the response to these events.

One important consideration in our experiments is the possible ambiguity of which neurons were stimulated. For all the experiments we targeted the LPO using viral injections paired with fibers overlaying the LPO or VTA. Even with the small viral injections (165–180 nl), there was almost always some expression in neighboring brain regions. We used fiber placement as the primary factor for including or excluding animals from our experiments because optogenetic ion channels are only activated when illuminated. Even though the majority of neuronal manipulations should occur under the fiber tip (Cole et al., 2018), we cannot exclude the possibility that we are also manipulating fibers of the passage of other, unintended brain regions that express ChR2. In the future, more precise targeting could be used to determine the exact neuronal populations that underlie our effects.

In conclusion, our results demonstrate that the LPO and the LPO→VTA pathway are functionally connected to the activity of VTA_Dopamine_ and VTA_GABA_ neurons. Stimulating the LPO and LPO→VTA pathway inhibits VTA_GABA_ neurons and both stimulates and inhibits VTA_Dopamine_ neurons. We also demonstrated that stimulation of the LPO and LPO→VTA pathway supports ICSS but also drives aversion, indicating that the LPO can regulate reinforcement and affective valence. Together these data also challenge the standard interpretation of the RTPT assay which is used throughout the field of affective neuroscience. Finally, we demonstrated that the LPO signals in response to aversive events but not rewarding events. Altogether these results indicate that the LPO is a player within the reward circuit and that further research is warranted to determine how these structures interact functionally to mediate reinforcement learning under both rewarding and punishing conditions.

## Data Availability Statement

The raw data supporting the conclusions of this article will be made available by the authors, without undue reservation.

## Ethics Statement

The animal study was reviewed and approved by the Institutional Animal Care and Use Committee of the University of Texas at Austin.

## Author Contributions

AG-F designed, conducted, and analyzed experiments, created figures, and wrote original draft. LG-F designed, conducted and analyzed experiments, edited figures and manuscript. SD conducted experiments, edited figures and manuscript. MM designed and analyzed experiments, and wrote original draft.

## Conflict of Interest

The authors declare that the research was conducted in the absence of any commercial or financial relationships that could be construed as a potential conflict of interest.
