# Supplementary material for: The Lateral Preoptic Area and Its Projection to the VTA Regulate VTA Activity and Drive Complex Reward Behaviors
Source: Front Syst Neurosci. 2020 Nov 3;14:581830. doi: 10.3389/fnsys.2020.581830 (PMC7669548; doi:10.3389/fnsys.2020.581830)
Supplement: Supplementary file 2 [file Data_Sheet_1.docx]

## Supplementary Figure 1: Validation of ChR2-mediated stimulation of LPO neurons


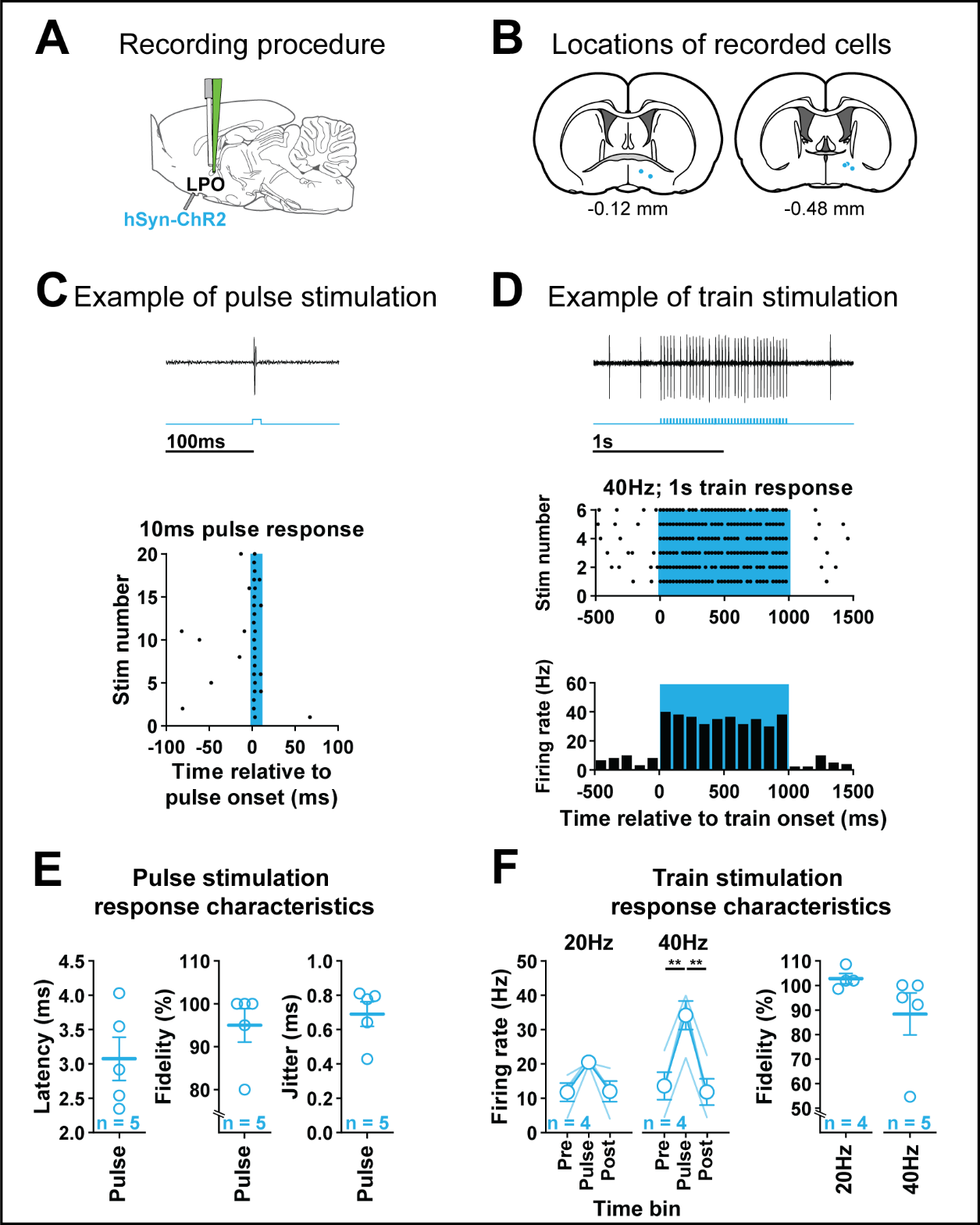


**(A)** Recording procedure: We injected hSyn-ChR2 in the LPO and recorded the activity of neurons in the LPO using an optrode. **(B)** Location of recorded neurons within the LPO. **(C)** Example of single neuron responding to 10ms 0.2Hz stimulation. Top: extracellular trace of the response to a single stimulation pulse; bottom: raster plot of spikes in response to 20 pulses, each dot represents a single action potential. **(D)** Single neuron example of response to 1s, 40Hz stimulation, 5ms pulses. Top: extracellular trace of the response to a single stimulation train; middle: raster plot of spikes in response to 6 trains; bottom: peristimulus time histogram showing firing rate in each 100ms time bin for raster plot shown above. **(E)** Response characteristics for pulse stimulation including the latency from pulse onset to action potential, fidelity (percent of pulses that resulted in an action potential), and the action potential jitter (sd of action potential latency); points depict values from individual rats; lines and error bars depict mean and sem, respectively. **(F)** Response characteristics for train stimulation including the firing rate in response to 20Hz and 40Hz trains (Pre: 2s prior to train onset, Pulse: 1s train, Post: 2s following train offset) and fidelity in response to 20Hz and 40Hz trains. Stimulation trains led to increased firing rate (time effect: F*_2,4_* = 498.16, P < 0.001; pulse train frequency effect: F*_1,4_* = 3.93, P = 0.18; interaction: F*_2,4_* = 4.22, P = 0.10). (HSD, ***: P < 0.001; **: P < 0.01); lines depict values from individual rats; points and error bars depict mean and sem, respectively.

## Supplementary Figure 2: Location of neurons within the VTA does not correlate with effect of LPO and LPO→VTA pathway stimulation


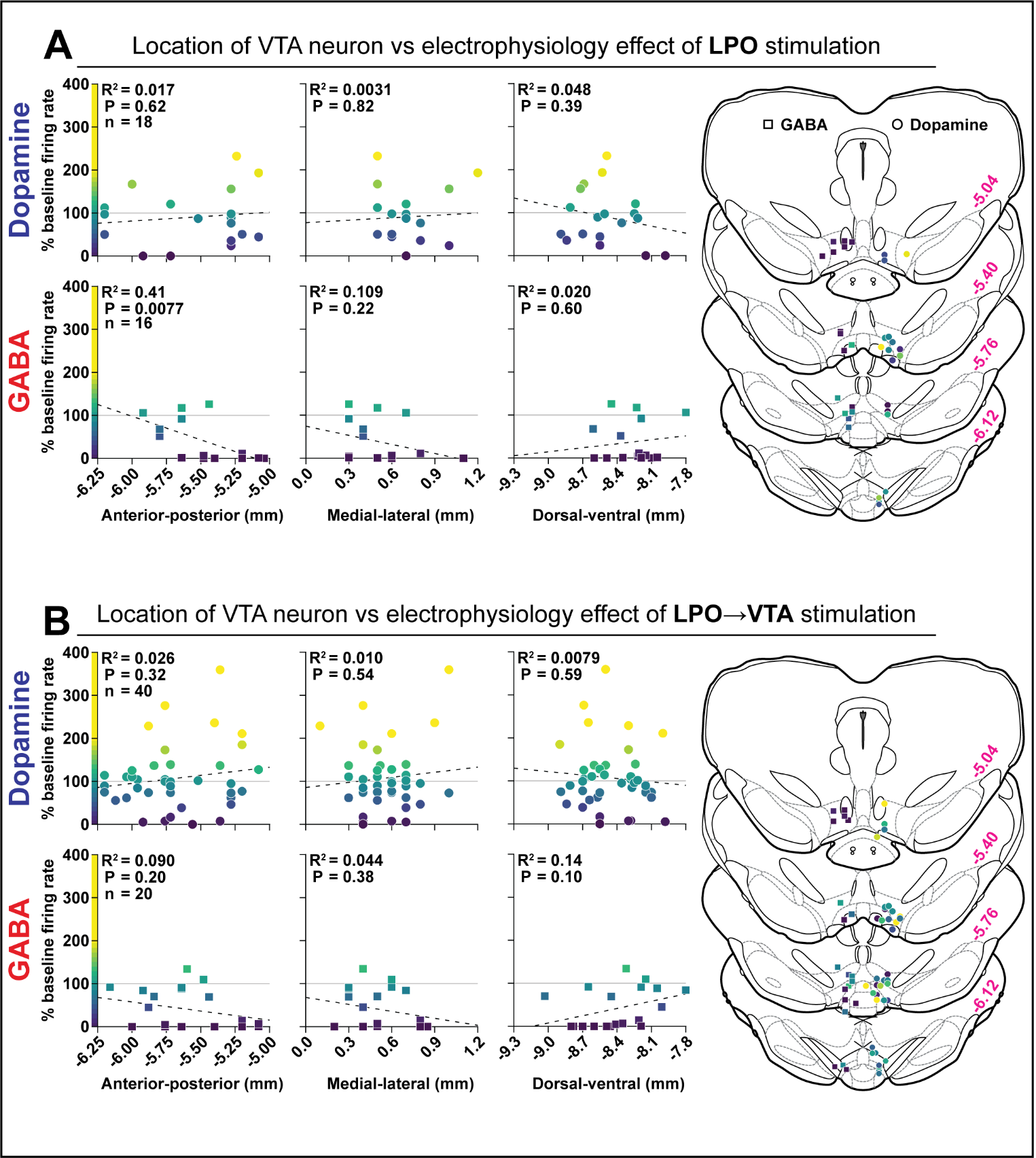


**(A)** Correlation between location of recorded neurons within the VTA and effects on firing rate of VTA_Dopamine_ and VTA_GABA_ neurons produced by stimulating the LPO. The anterior-posterior (left), medial-lateral (middle), and dorsal-ventral (right) location of VTA_Dopamine_ neurons (top row) and VTA_GABA_ neurons (bottom row) did not correlate with the change in firing rate produced by stimulating the LPO. The one exception was the anterior-posterior location of GABA neurons, which showed a greater inhibition in the rostral portions of the VTA. Inset shows the back-calculated location of neurons within the VTA. For all plots in (A), the color of points indicates the effect of LPO stimulation (scale depicted on the left side of the scatter plots; yellow: excited; purple: inhibited). **(B)** Correlation between location of recorded neurons in the VTA and effects on firing rate of VTA_Dopamine_ and VTA_GABA_ neurons produced by stimulating the LPO-VTA pathway. The anterior-posterior (left), medial-lateral (middle), and dorsal-ventral (right) location of VTA_Dopamine_ neurons (top) and VTA_GABA_ neurons (bottom) did not correlate with the change in firing rate produced by stimulating the LPO→VTA pathway. Inset shows the back-calculated location of neurons within the VTA. For all plots in (B), the color of points indicates the effect of LPO→VTA pathway stimulation (color scale is depicted on the left side of the scatter plots; yellow: excited; purple: inhibited). There may be overlapped data points at 0% baseline firing for scatter plots.

## Supplementary Figure 3: Placement of optic fibers in the LPO does not correlate with effect of LPO stimulation


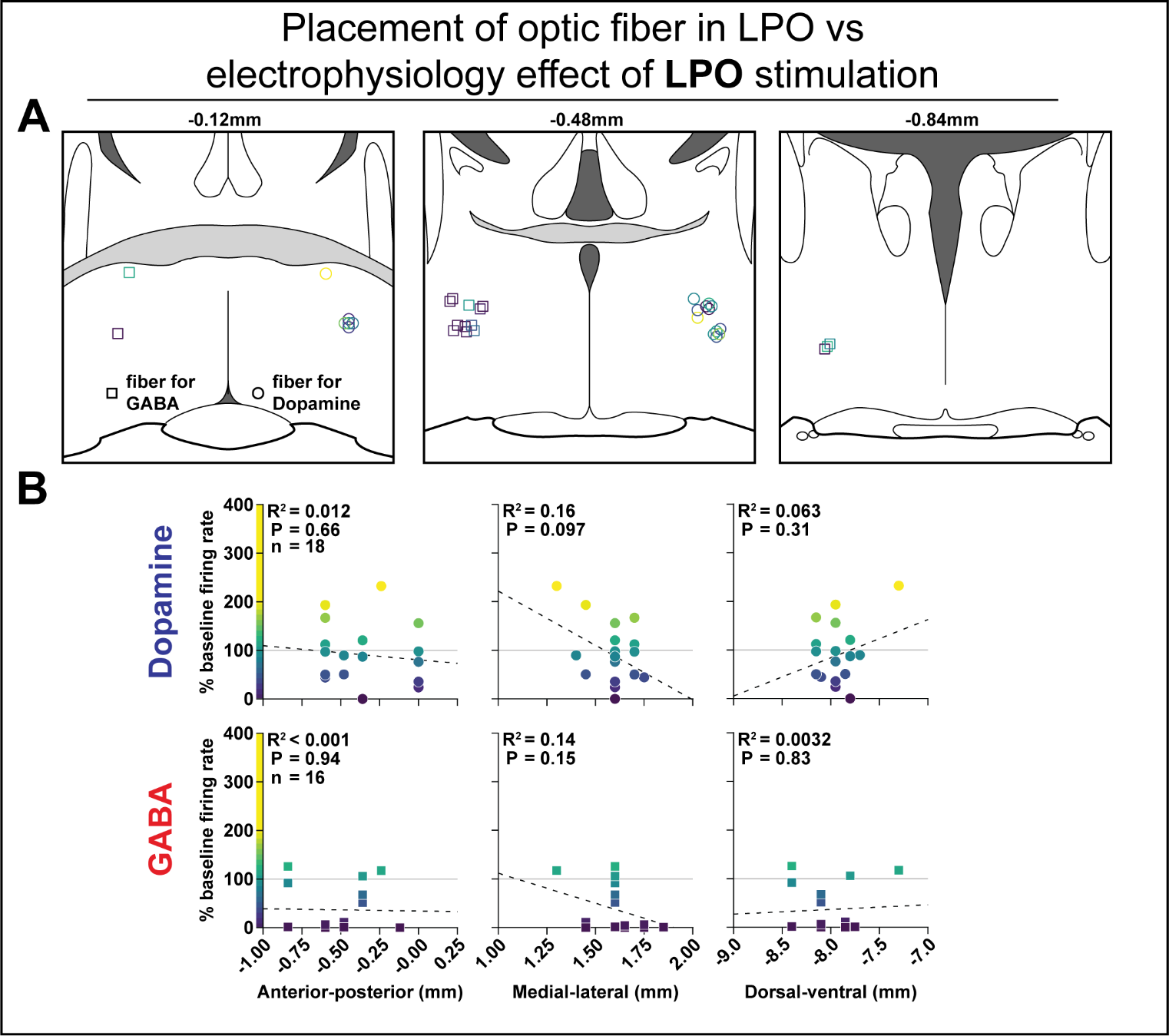


**(A)** Placement of the optic fibers within the LPO. Jitter was manually added to prevent overlap of optic fiber position when multiple cells were recorded for a single fiber position. **(B)** Correlation between placement of the optic fibers in the LPO and effects on firing rate. The anterior-posterior (left), medial-lateral (middle), and dorsal-ventral (right) placement of the optic fiber within the LPO did not correlate with effects on VTA_Dopamine_ neurons (top row) or VTA_GABA_ neurons (bottom row). For all plots, the color of points indicates the effect of LPO stimulation (color scale is depicted on the left side of the scatter plots; yellow: excited; purple: inhibited). There may be overlapped data points at 0% baseline firing for scatter plots.

## Supplementary Figure 4: Comparison of long and short trains for optogenetic stimulation of the LPO and LPO→VTA pathway


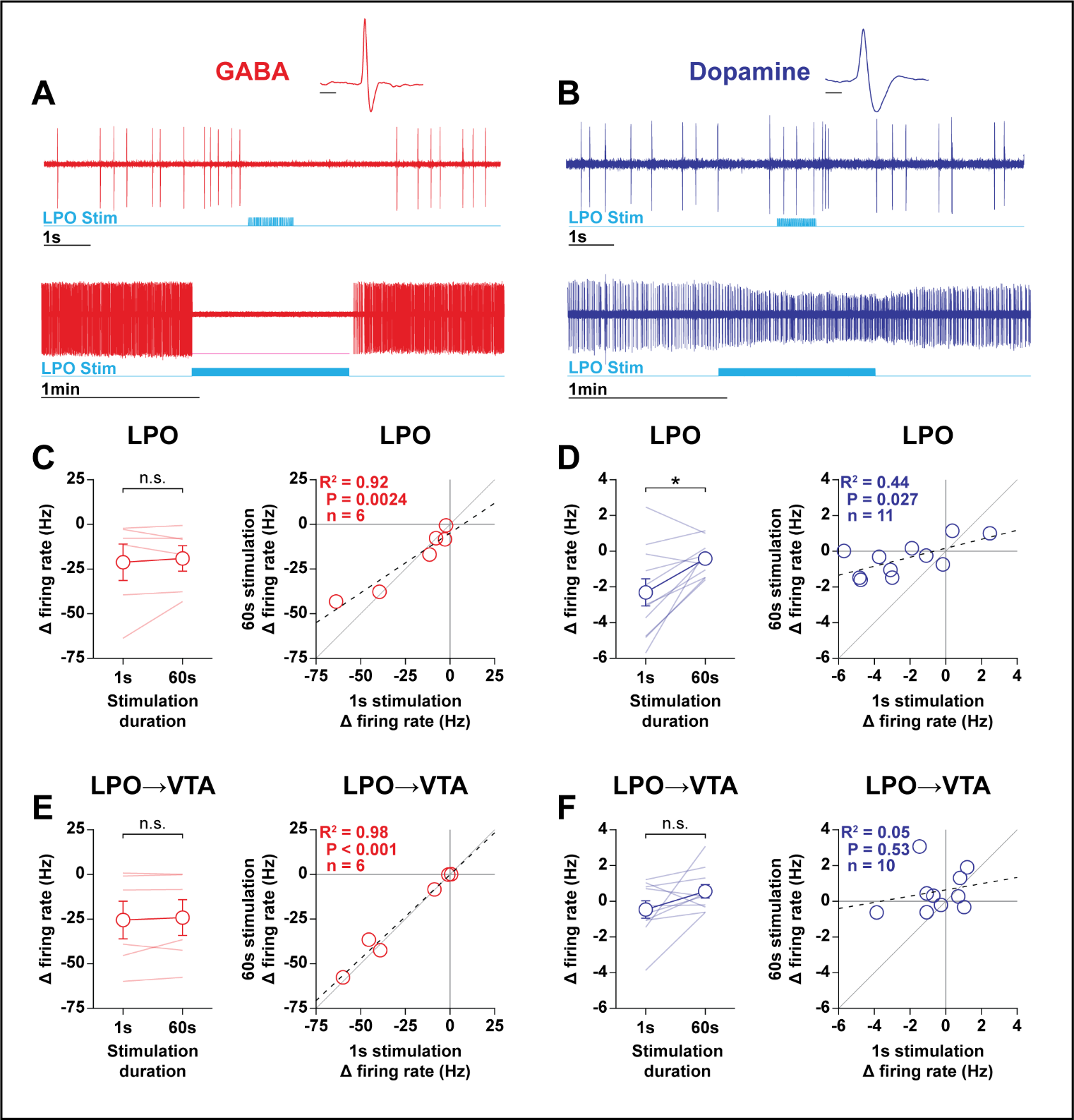


**(A)** Representative VTA_GABA_ (red) neuron during stimulation of the LPO showing the extracellular waveform and inhibitory response to LPO laser stimulation (40Hz, 5ms pulses, 20mW) during a 1s train (top), and 60s train (bottom). **(B)** Representative VTA_Dopamine_ (blue) neuron during stimulation of the LPO showing the extracellular waveform and excitatory response to LPO laser stimulation during a 1s train (top), and 60s train (bottom). General format for **(C-F)**: left plot: line plot depicting change in firing rate during the 1s train relative to the 10s baseline prior to the first stimulation train for 1s and 60s stimulation durations (*: *t-*test, P < 0.05); right plot: scatter plot depicting the relationship and correlation between the change in firing for 1s and 60s stimulation durations. **(C)** 1s and 60s stimulation of the LPO had similar effects on VTA_GABA_ neurons. **(D)** Compared with 1s stimulation, 60s stimulation of the LPO produces higher firing rates for VTA_Dopamine_ neurons (left). **(E)** 1s and 60s stimulation of the LPO→VTA pathway had similar effects on VTA_GABA_ neurons. **(F)** 1s and 60s stimulation of the LPO→VTA pathway had similar effects on VTA_Dopamine_ neurons. In line plots, faded lines depict values of individual rats; points and error bars depict mean and sem, respectively. In correlation plots, the hashed line depicts the regression line and the solid line depicts a slope of 1.

## Supplementary Figure 5: The LPO supports intracranial self-stimulation responding across cohorts and stimulation parameters


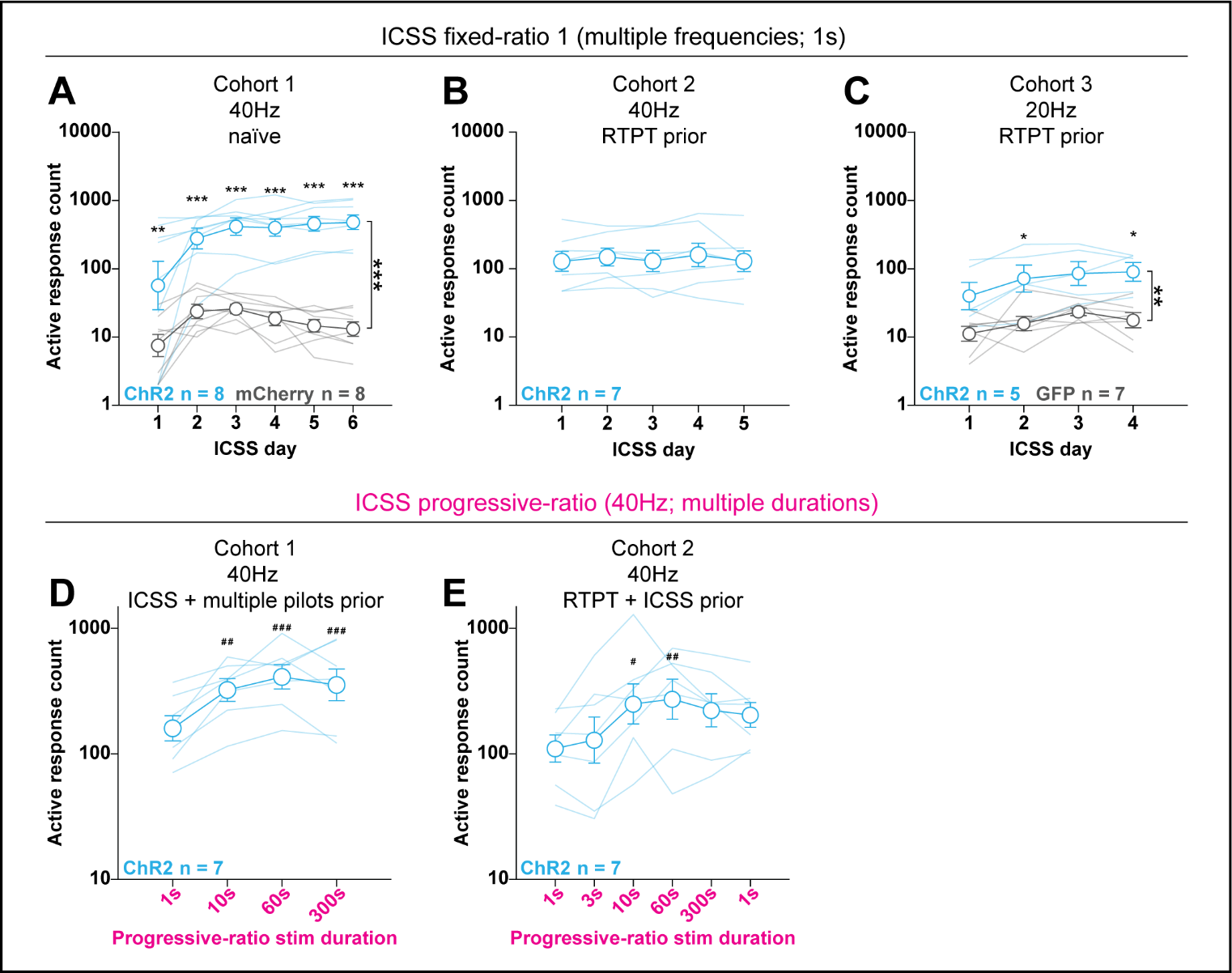


**(A-C)** Active hole responses during intracranial self-stimulation (ICSS) at a fixed-ratio 1 for 1s stimulation in 3 separate cohorts of rats. **(A)** Prior to ICSS, Cohort 1 did not undergo any behavioral procedures. The ChR2 group made more active hole responses than the mCherry group throughout the ICSS procedure (group x hole interaction: F*_1,14_* = 50.28, P < 0.001). **(B)** Prior to ICSS, Cohort 2 underwent RTPT with 40Hz 3s trains and 3s inter-train intervals. Data are shown for comparison to other cohorts. **(C)** Prior to ICSS, Cohort 3 underwent RTPT with 20Hz continuous stimulation. The ChR2 group made more active hole responses than the GFP group throughout the ICSS procedure (group x hole interaction: F*_1,10_* = 15.71, P = 0.0027). **(D-E)** Active hole responses during ICSS in a progressive ratio schedule in 2 separate cohorts. **(D)** Prior to PR, Cohort 1 underwent RTPT and multiple pilot experiments. The ChR2 group made more responses for longer stimulation durations compared with the shorter (1s) stimulation duration. **(E)** Prior to PR, Cohort 2 underwent RTPT and ICSS training. The ChR2 group made more responses for longer stimulation durations compared with the shorter (1s) stimulation duration. (HSD ChR2 vs mCherry, **: P < 0.01, ***, P < 0.001; HSD vs. 1s, #: P < 0.05, ## P < 0.01, ### P < 0.001); in (A-E), faded lines depict values of individual rats; points and error bars depict mean and sem, respectively.

## Supplementary Figure 6: Placement of optic fiber placements for optogenetic stimulation of LPO cell bodies


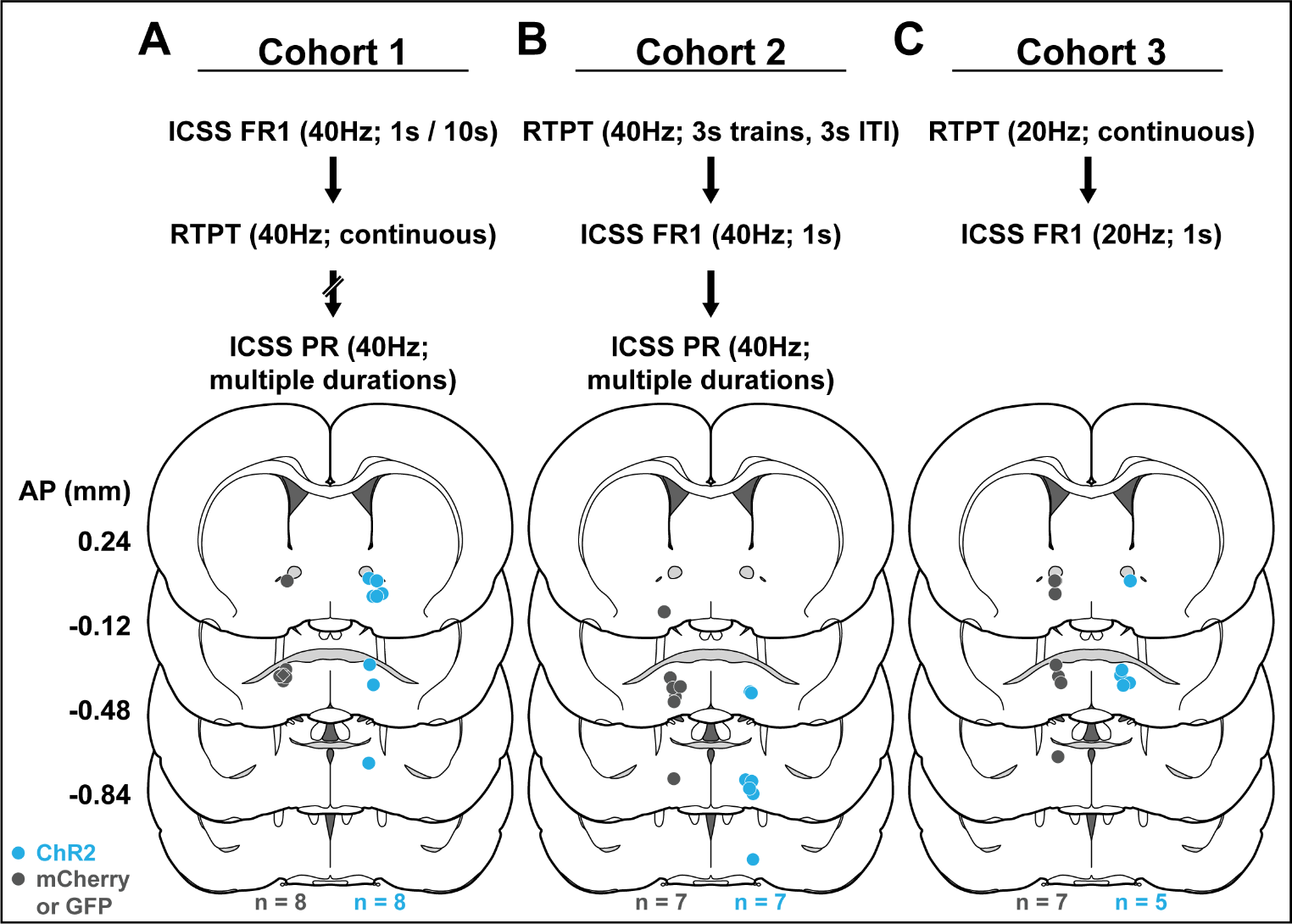


**(A-C)** Order of experimental procedures (top) and placement of optic fibers within the LPO (bottom) for ICSS and RTPT experiments stimulating LPO cell bodies in the mCherry or GFP group (grey) or the ChR2 group (blue). Cohort descriptions can be found the main body of the text (results and figure legends). All placements were on the right side of the brain. Single diamond point in Cohort 1 depicts an mCherry subject that was ran in ICSS but not RTPT; all other subjects were ran in both ICSS and RTPT.

## Supplementary Figure 7: Placement of optic fibers in the LPO does not correlate with ICSS responding


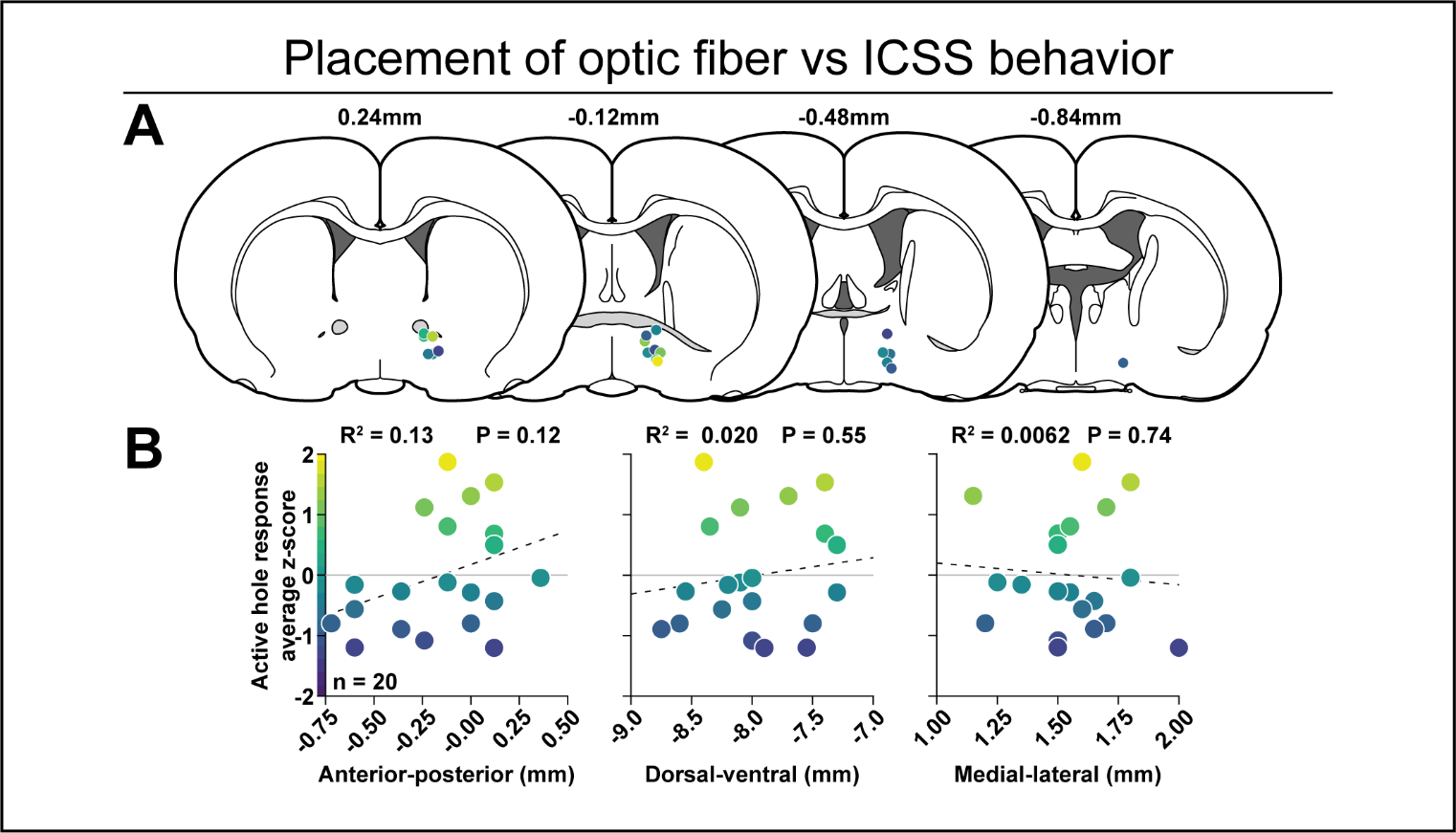


**(A)** Placement of optic fibers within the LPO. **(B)** Correlation between placement of the optic fibers in the LPO and response rates in ICSS. The anterior-posterior (left), medial-lateral (middle), and dorsal-ventral (right) placement of the optic fiber within the LPO did not correlate with ICSS responding (z-score of the mean response count during the last three days of ICSS). Data points are combined from cohorts 1-3 and z-scores were calculated within each experiment in order to normalize across stimulation parameters. For all plots, colors depict the magnitude of ICSS (color scale is depicted on left side of scatter plots; yellow: high responding; purple: low responding).

## Supplementary Figure 8: Single rat example of RTPT aversion and ICSS reinforcement


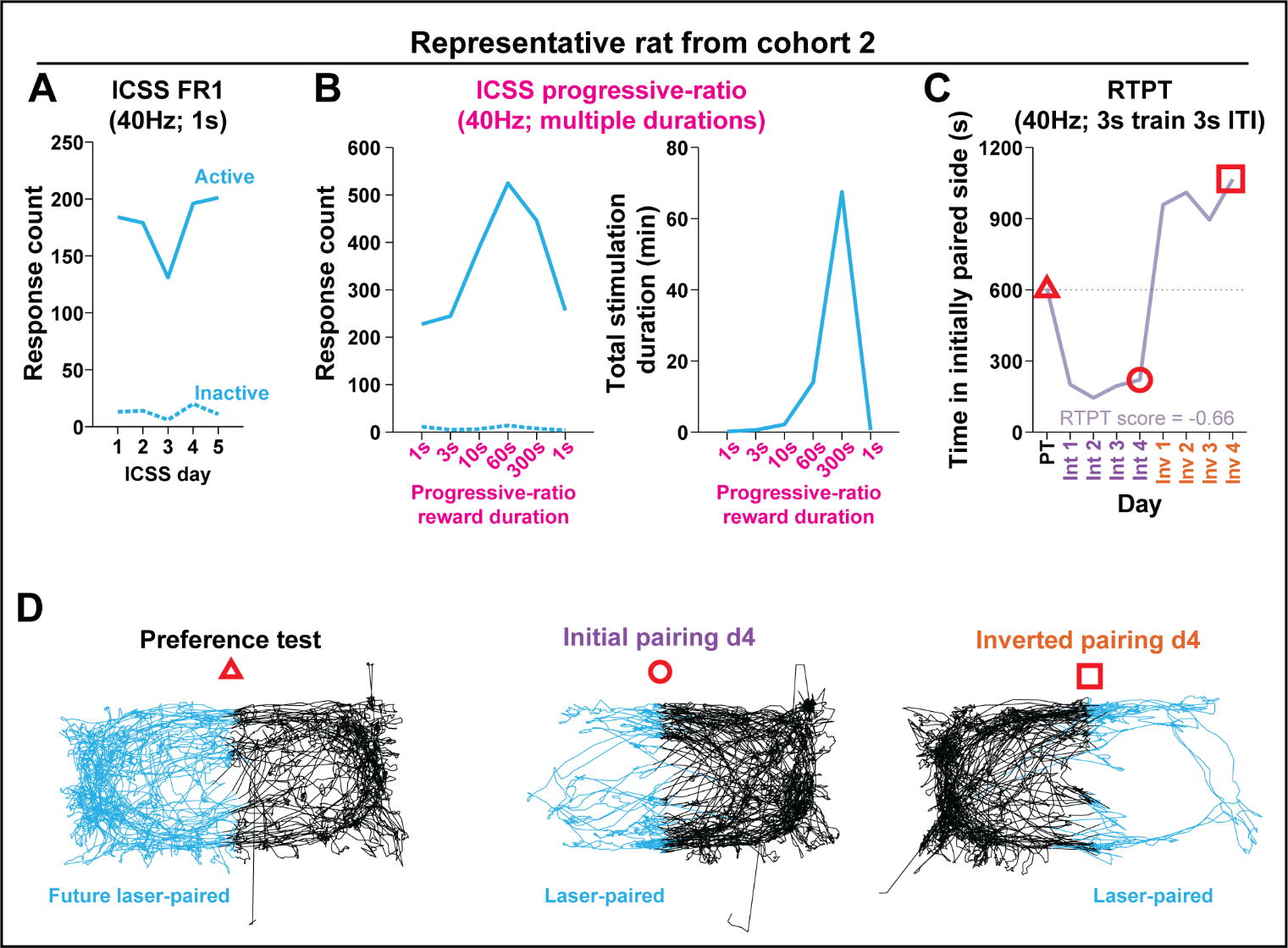


**(A)** Response rates in the intracranial self-stimulation (ICSS) test at fixed-ratio (FR) 1 indicate the reinforcing properties of LPO stimulation; the solid line depicts active hole responding and the dashed line depicts inactive hole responding. **(B)** Response rates (left) in the progressive-ratio task indicate the motivating property of LPO stimulation; line types match those of (A). Total stimulation duration earned within the progressive-ratio task (right) shows that the rat earns more stimulation during progressive-ratio test than the entire 20-minute duration of real-time place testing (RTPT). **(C)** RTPT behavior shows clear aversion, as indicated by avoidance of the side paired with LPO stimulation. **(D)** traces depicting the rats center point in RTPT during the preference test, last day of initial pairing, and last day of inverted pairing; blue color indicates future laser pairing side (preference test) or the laser paired side (initial pairing / inverted pairing).

## Supplementary Figure 9: Crossing events across days of RTPT


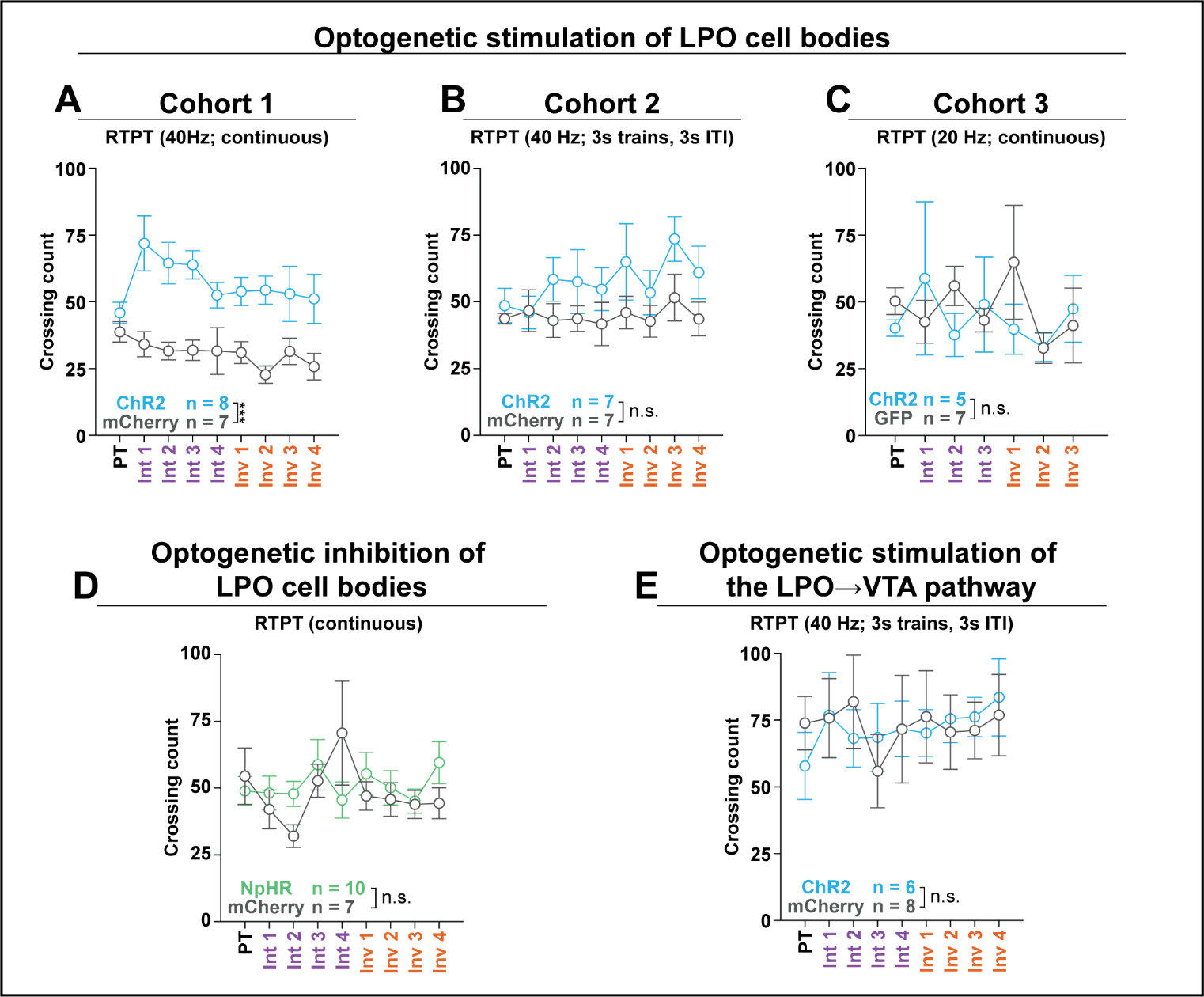


**(A-E)** Number of crossings across real-time place testing (RTPT). **(A)** In Cohort 1 (stimulation of LPO cell bodies, 40Hz, continuous) the ChR2 group showed an enhanced crossing between sides of the RTPT apparatus relative to the mCherry group (group effect: F*_1,13_* = 20.02, P < 0.001). **(B)** In Cohort 2 (stimulation of LPO cell bodies, 40Hz, 3s train, 3s ITI) the ChR2 group did not show differences in crossings relative to the mCherry group (group effect: F*_1,12_* = 3.08, P = 0.10). **(C)** In Cohort 3 (stimulation of LPO cell bodies, 20Hz, continuous) the ChR2 group did not show differences in crossings relative to the GFP group (group effect: F*_1,10_* = 0.18, P = 0.67). **(D)** In the experiment testing inhibition of LPO cell bodies, the NpHR group did not show differences in crossings relative to the mCherry group (group effect: F*_1,15_* = 0.20, P = 0.66). **(E)** In the experiment testing stimulation of the LPO→VTA pathway, the ChR2 group did not show differences in crossings relative to the mCherry group (group effect: F*_1,12_* = 0.0014, P = 0.97). In (A-E), points and error bars depict mean and sem, respectively.

## Supplementary Figure 10: Stimulation of the LPO with low frequency does not promote real-time place aversion or perference


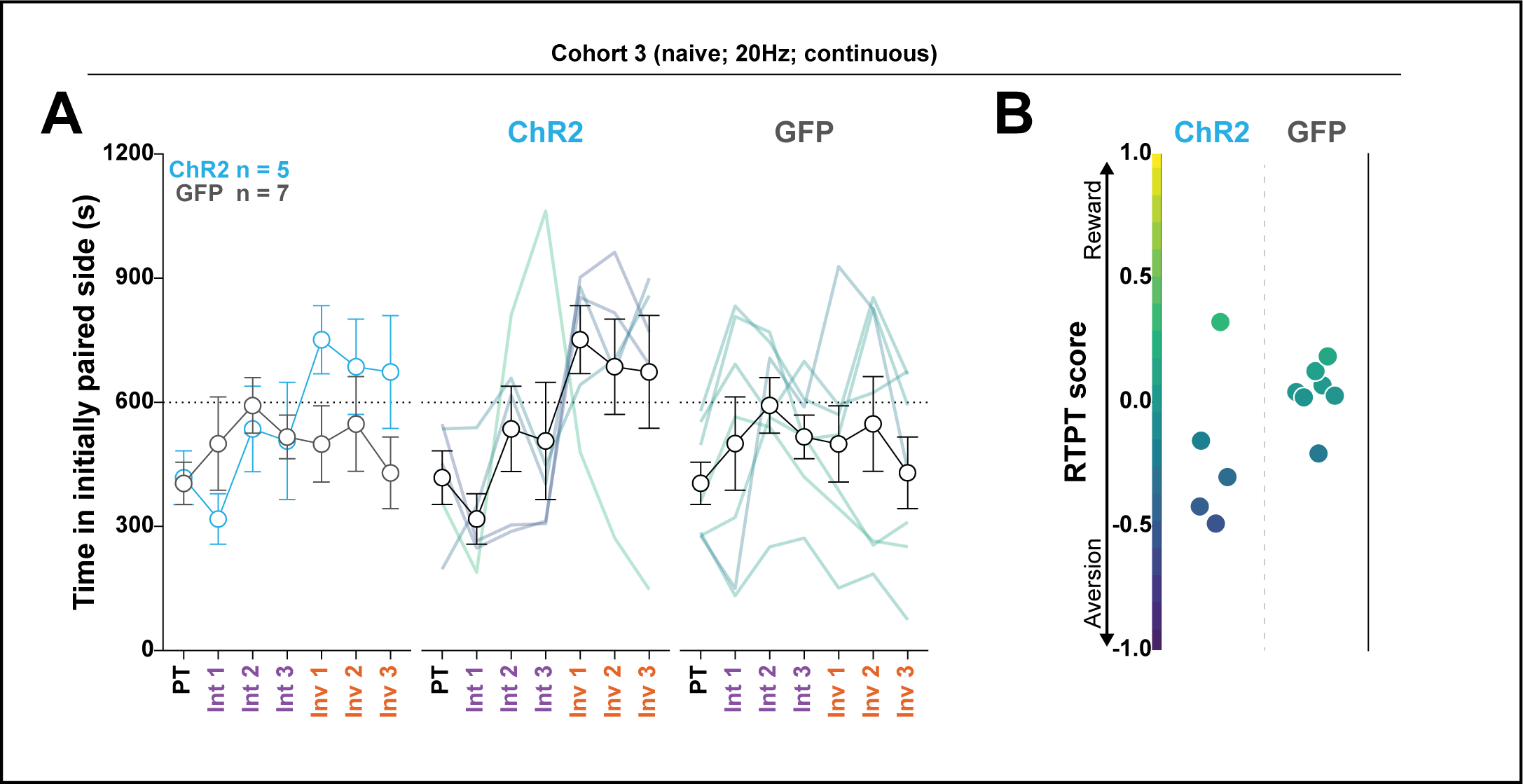


**(A)** Mean time in the initially paired side over RTPT, in ChR2 (blue) and GFP (grey) groups. Lines depict single rats, which are color-coded based on RTPT score. Unlike in Cohort 1 and Cohort 2, the least preferred side during the preference test was assigned as the initially paired side, to enhance the ability to detect preference. ChR2 and the GFP groups showed similar mean effects across days of RTPT (group x day interaction: F*_6,60_* = 2.05, P = 0.072. **(B)** RTPT scores for rats in the ChR2 and the GFP groups. In (A), points and error bars depict mean and sem, respectively.

## Supplementary Figure 11: Placement of optic fibers in the LPO does not correlate with RTPT behavior


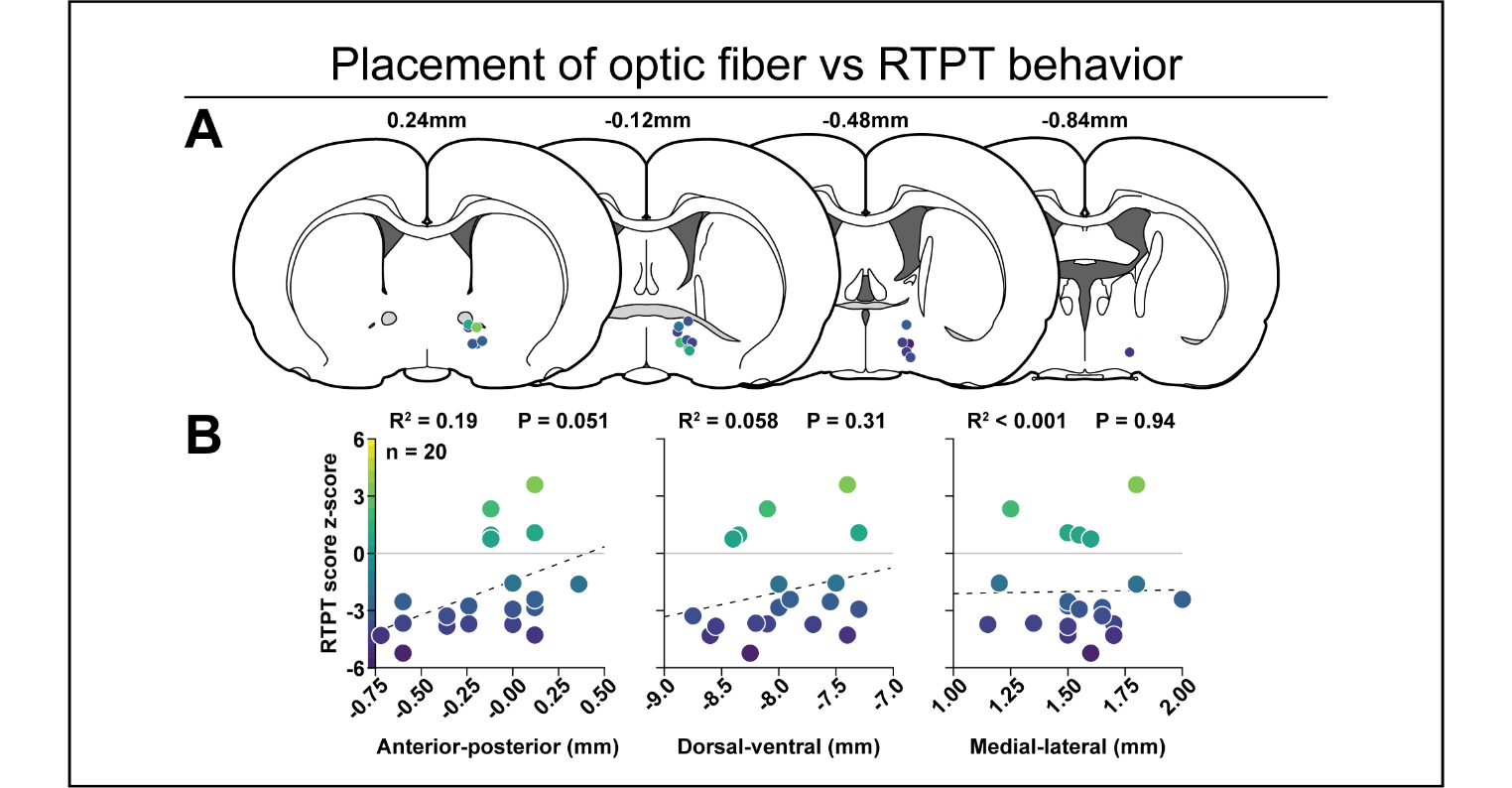


**(A)** Placement of optic fibers within the LPO. **(B)** Correlation between placement of the optic fibers in the LPO and real-time place testing (RTPT) behavior. The anterior-posterior (left), medial-lateral (middle), and dorsal-ventral (right) placement of the optic fiber within the LPO did not correlate with the normalized RTPT score (z-score of the RTPT score using the standard deviation of the control group). Data points are combined from cohorts 1-3. For all plots, the color of points indicates the RTPT (color scale is depicted on left side of scatter plots; yellow: High normalized responding; purple: Low normalized responding).

## Supplementary Figure 12: Placement of optic fiber placements for optogenetic stimulation of LPO cell bodies and the LPO→VTA pathway


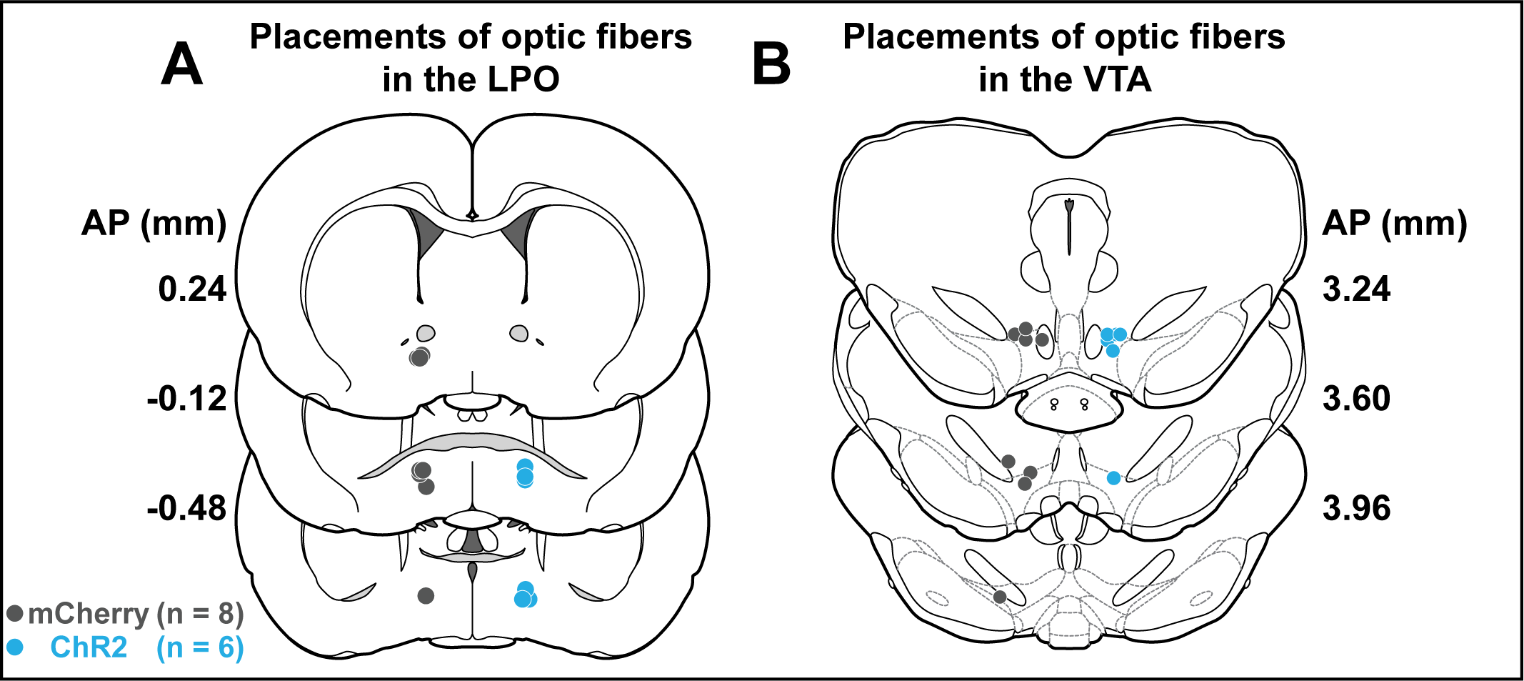


**(A-B)** Placement of the optic fibers placed unilaterally within the LPO (A) and LPO→VTA pathway (B) in the ChR2 (blue) and mCherry (grey) groups. All placements were on the right side of the brain.

## Supplementary Figure 13: Across stimulation parameters, RTPT and ICSS behavior are correlated but rats obtain more stimulation during RTPT.


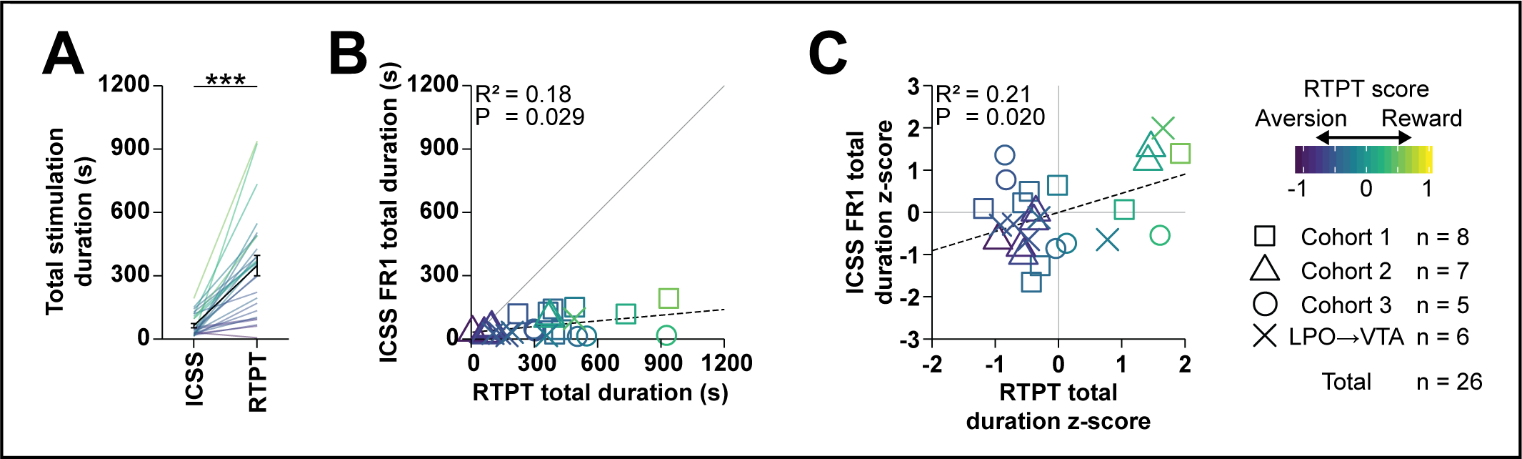


**(A)** Across experimental parameters, the total stimulation duration earned in RTPT was higher than in ICSS (Wilcoxon, ***: P < 0.001). **(B)** Across experimental parameters, the stimulation duration earned within RTPT and ICSS is correlated but substantially lower in the ICSS compared with RTPT; shapes indicate the experimental group of each data point. **(C)** Normalizing the amount of stimulation obtained by taking the z-score of the ChR2 rats within each experimental group reveals a correlation between the amount of stimulation obtained in ICSS and RTPT; shapes indicate the experimental group of each data point.

## Supplementary Figure 14: Across stimulation parameters, the stimulation-interval underlies RTPT behavior to a greater degree than inter-stimulation-interval


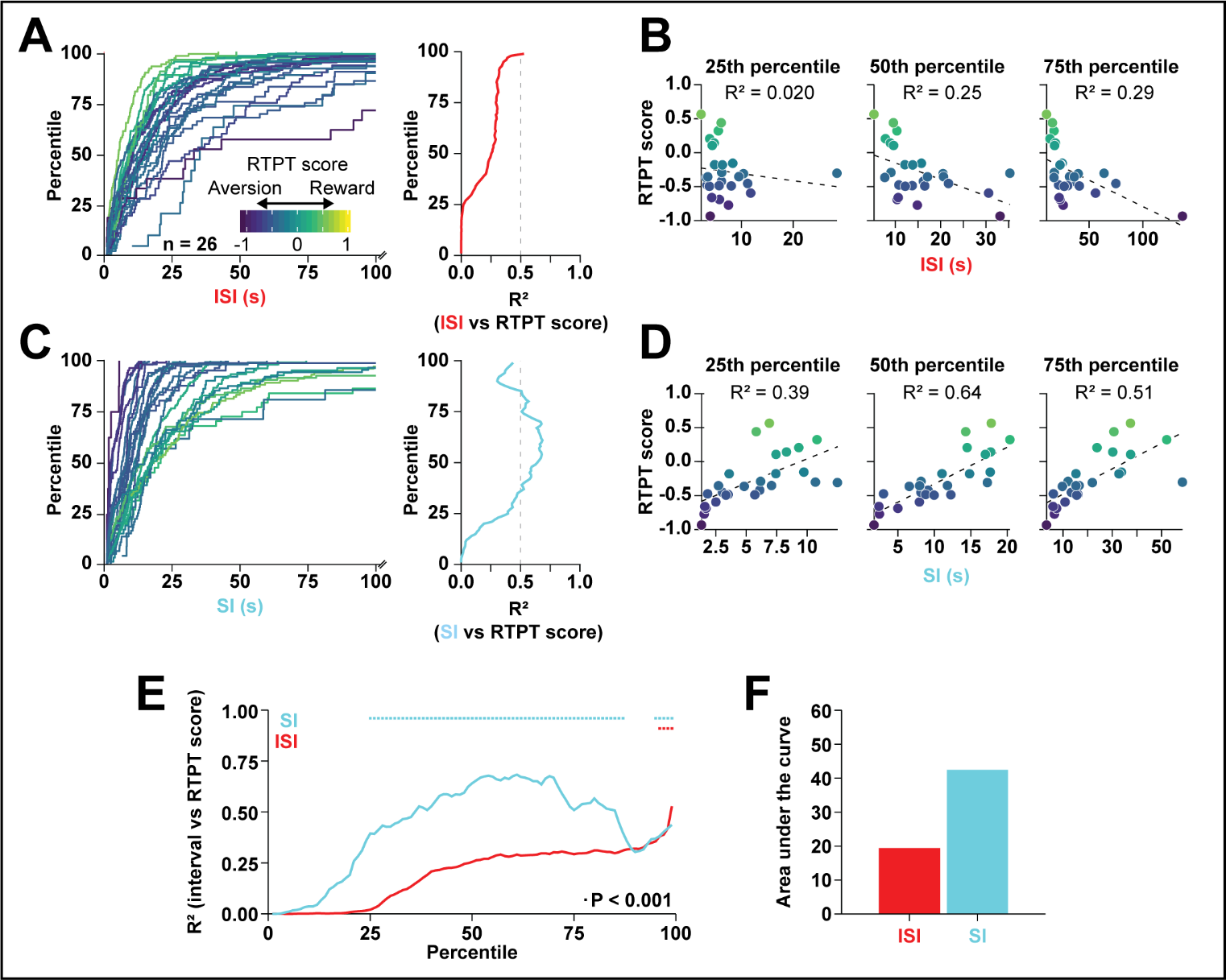


**(A)** Data combined across experiments with different stimulation parameters including both LPO and LPO-VTA ChR2 experiments included throughout the paper. Left: Cumulative distribution function (CDF) for inter-stimulation-interval (ISI) within real-time place testing (RTPT) for each rat, color coded by RTPT score (inset indicates scale). Right: Correlation of ISI vs. total ICSS duration earned from percentiles 1 to 99 indicate a relatively weak relationship between variables across percentiles. **(B)** Correlations at each percentile indicate poor correlations between the ISI and RTPT score. **(C)** CDF for SI within RTPT for each subject, color coded by RTPT score. Right: Correlation of ISI vs. total ICSS duration earned from percentiles 1 to 99 indicate a strong correlation between variables across percentiles. **(D)** Correlations at each percentile indicate strong correlations between the stimulation-interval and RTPT score. **(E)** Overlay of the curves shown in the left side of (A) and (C) demonstrate substantially greater correlation of the stimulation-interval compared withISI; dots indicate interval percentiles that show a correlation with P < 0.001. **(F)** Area under the curve for (E) further indicates that the SI correlates more strongly with the RTPT score compared with the ISI.

## Supplementary Figure 15: Placement of optic fibers for optogenetic stimulation of LPO cell bodies in the RTPT electricity procedure


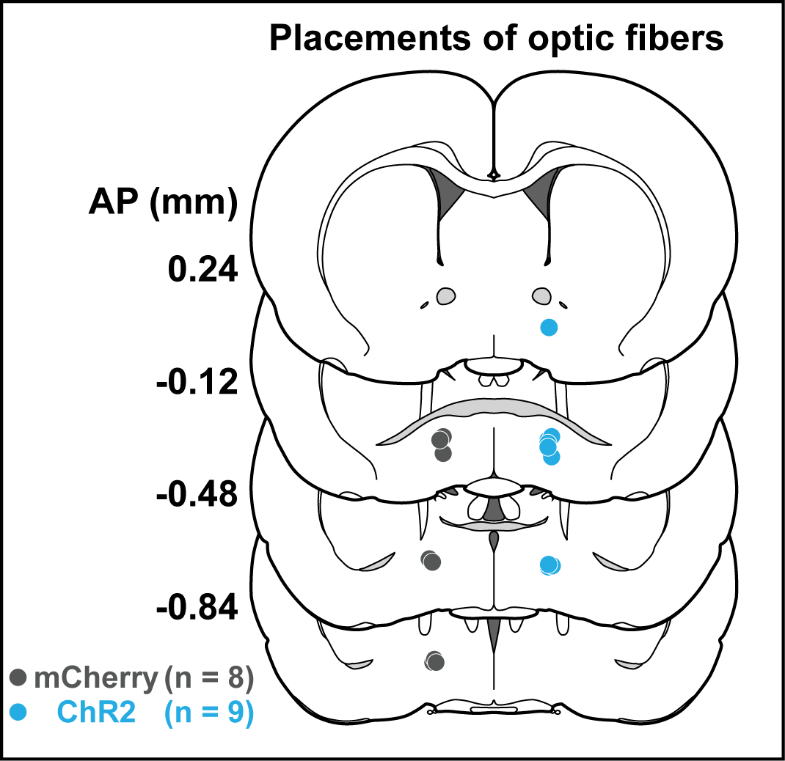


Placement of the optic fibers placed unilaterally within the LPO illumination in the mCherry (grey) and ChR2 (blue) groups. All placements were on the right side of the brain.

## Supplementary Figure 16: Binned preference during the RTPT electricity procedure


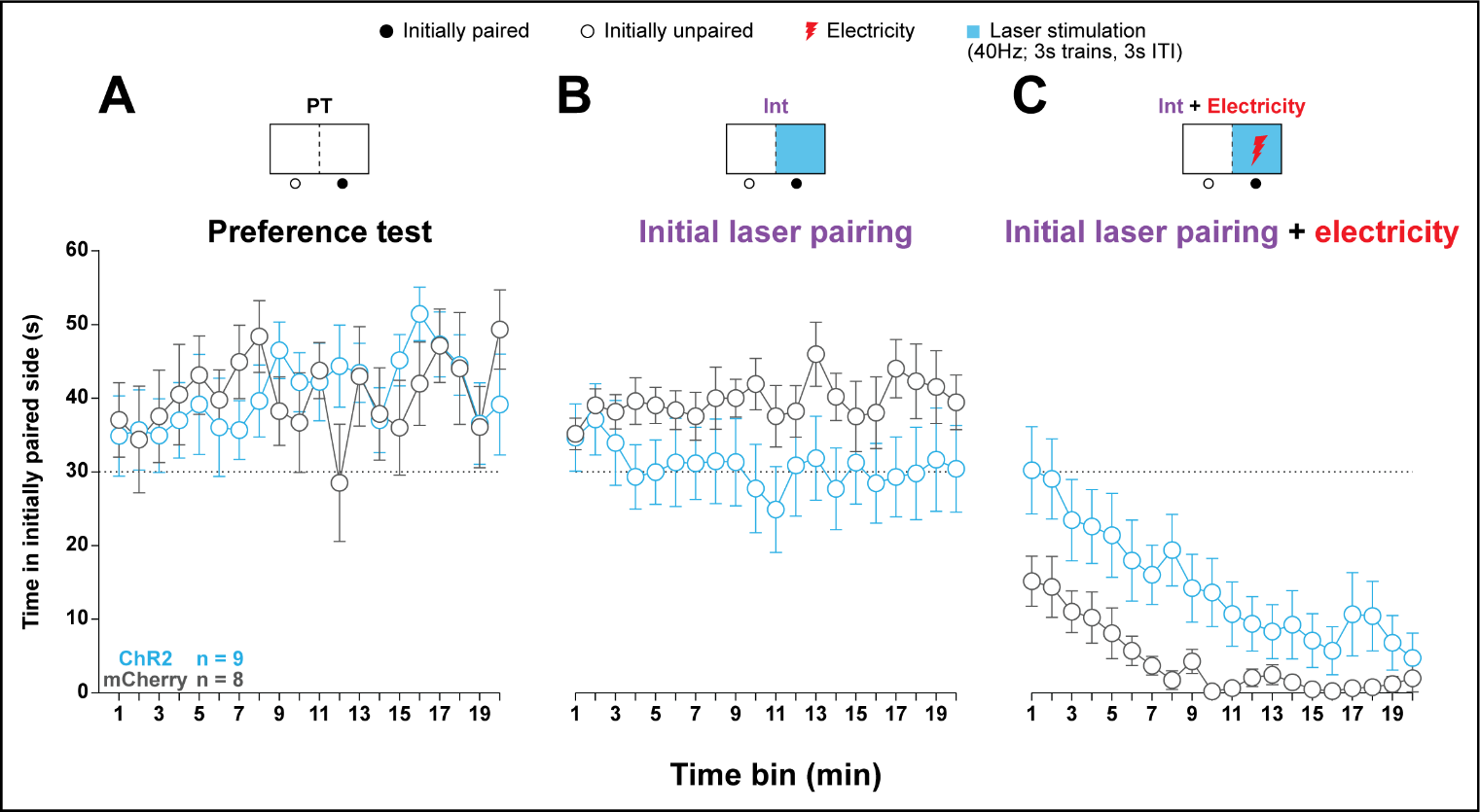


**(A-C)** Time spent in the initially paired side across training stages. **(A)** Mean behavior during the preference test. **(B)** Mean behavior across all 4 days of initial pairing (average across days). **(C)** Mean behavior during the last 4 days of initial pairing + 0.15mA electricity (average across days). Results indicate differential behavior between the mCherry and ChR2 groups depending on training stage (group x stage interaction: F*_2,30_* = 4.09, P = 0.027). During the dual laser + electricity pairing, the ChR2 group spent a greater amount of time in the dual-paired side compared with the mCherry group and this occurred similarly throughout the session (group effect: F*_1,15_* = 5.17, P = 0.038; group x time interaction: F*_19,285_* = 1.77, P = 0.026). In (A-C), points and error bars depict mean and sem, respectively.

## Supplementary Figure 17: Validation of NpHR mediated inhibition of LPO neurons


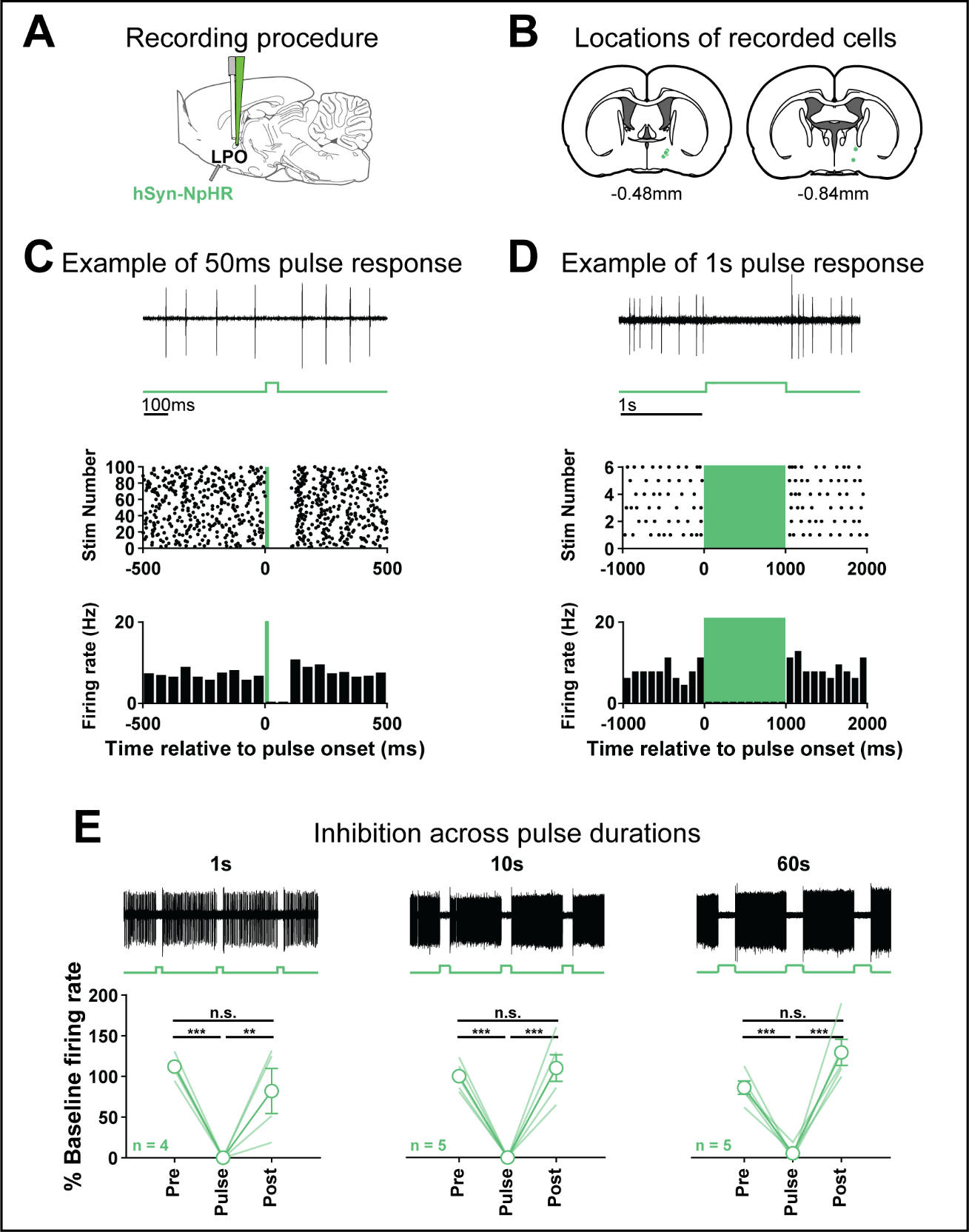


**(A)** Recording procedure: We injected hSyn-NpHR in the LPO and recorded LPO neurons with an optrode. **(B)** Location of recorded neurons. **(C)** Example of a single neuron responding to 50ms, 0.2Hz illumination pulses. Top: Extracellular trace of the response to a single illumination pulse; middle: Raster plot of spikes in response to 100 pulses, each dot represents a single action potential; bottom: Peristimulus time histogram showing firing rate in each 100ms bin for t raster plot shown above. **(D)** single neuron example of response to 1s, 0.1Hz illumination. Top: Extracellular trace of the response to a single pulse; middle: Raster plot of spikes in response to 6 pulses; bottom: Peristimulus time histogram showing firing rate in each 100ms bin for the raster plot shown above. **(E)** Responses to pulses of 1s, 10s, and 60s duration. Top: Single neuron inhibitory responses to each pulse duration; bottom: Binned firing rate divided by 10s baseline firing rate in response to each pulse duration (Pre: 2s bin prior to train onset; Pulse: 1s train; Post: 2s bin following train offset). As shown, every neuron recorded showed complete inhibition across pulse durations (time effect: F*_2,22_* = 81.79, P < 0.001; pulse duration effect: F*_2,11_* = 2.75, P = 0.76; interaction: F*_4,22_* = 4.22, P = 0.062; ***: HSD, P < 0.001, **: HSD, P < 0.01). In (E), faded lines depict singular rat values; points and error bars depict mean and sem, respectively.

## Supplementary Figure 18: Placement of optic fibers for optogenetic inhibition of LPO cell bodies


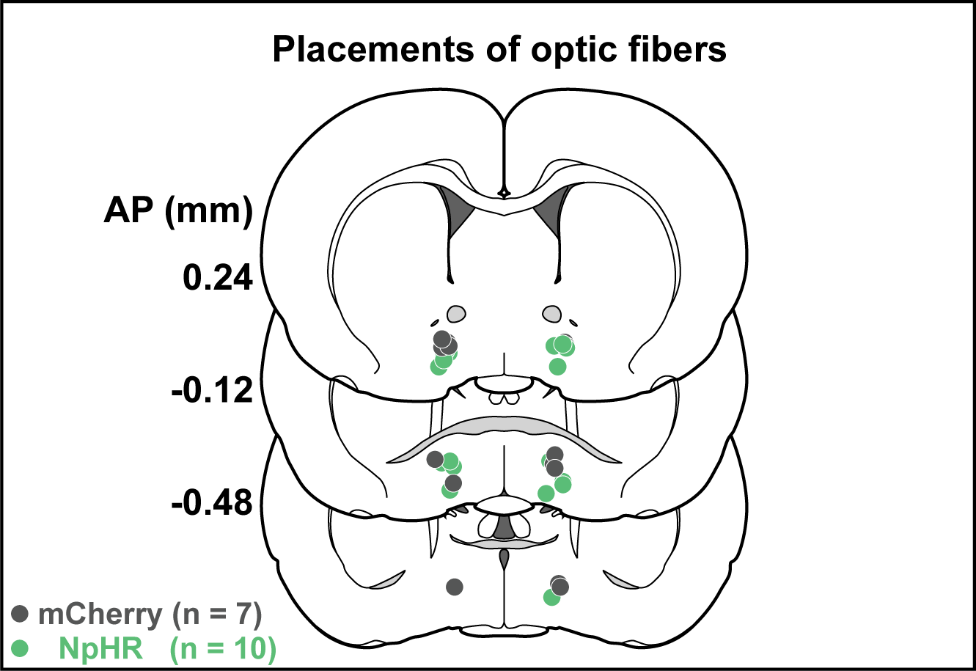


Placement of optic fibers placed bilaterally within the LPO for illumination in the halorhodopsin (green) and mCherry (grey) groups.

## Supplementary Figure 19: Fiber photometry analysis and placements of optic fibers


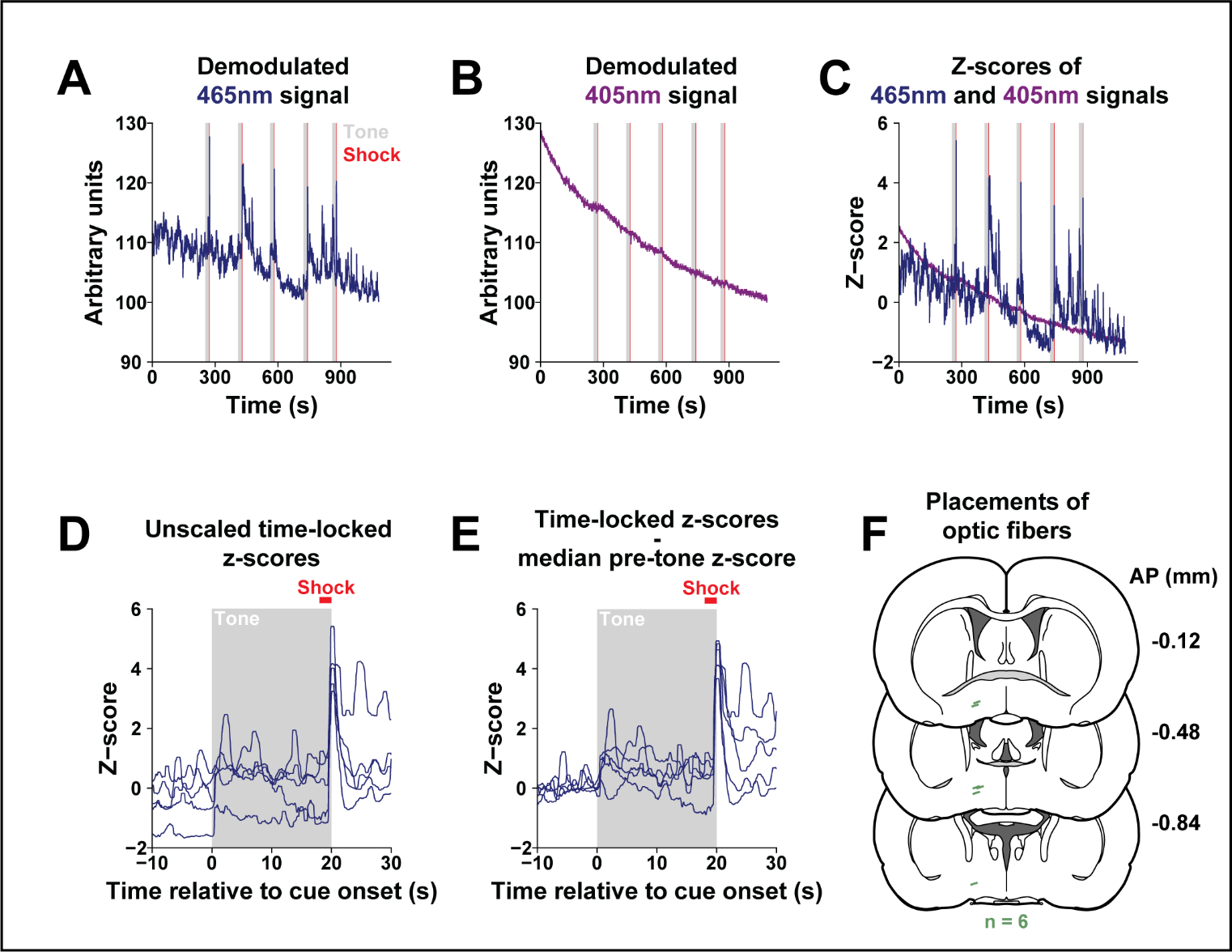


**(A)** LPO fiber photometry raw 465nm GCaMP and **(B)** raw 405nm autofluorescent channel recording during Pavlovian conditioning for foot-shock. **(C)** The z-score was taken for both channels for the entire session duration. **(D)** Single trial perievent signals of the z-score of the 465nm GCaMP channel. **(E)** To reduce the effect of downward baseline drift, perievent signals of both channels were subtracted by the median baseline. **(B)** Raw 405nm autofluorescent (10s prior to event). Group mean and sem of perievent signals were created by calculating the mean of all recorded signals (n = number of subject x trials). Mean area under the curve was calculated for each subject independently, and then these means were used to calculate group mean and sem. **(F)** Fiber photometry placements of fibers in the LPO. All placements were on the right side of the brain.

## Supplementary Figure 20: Conditioning timeline and procedures


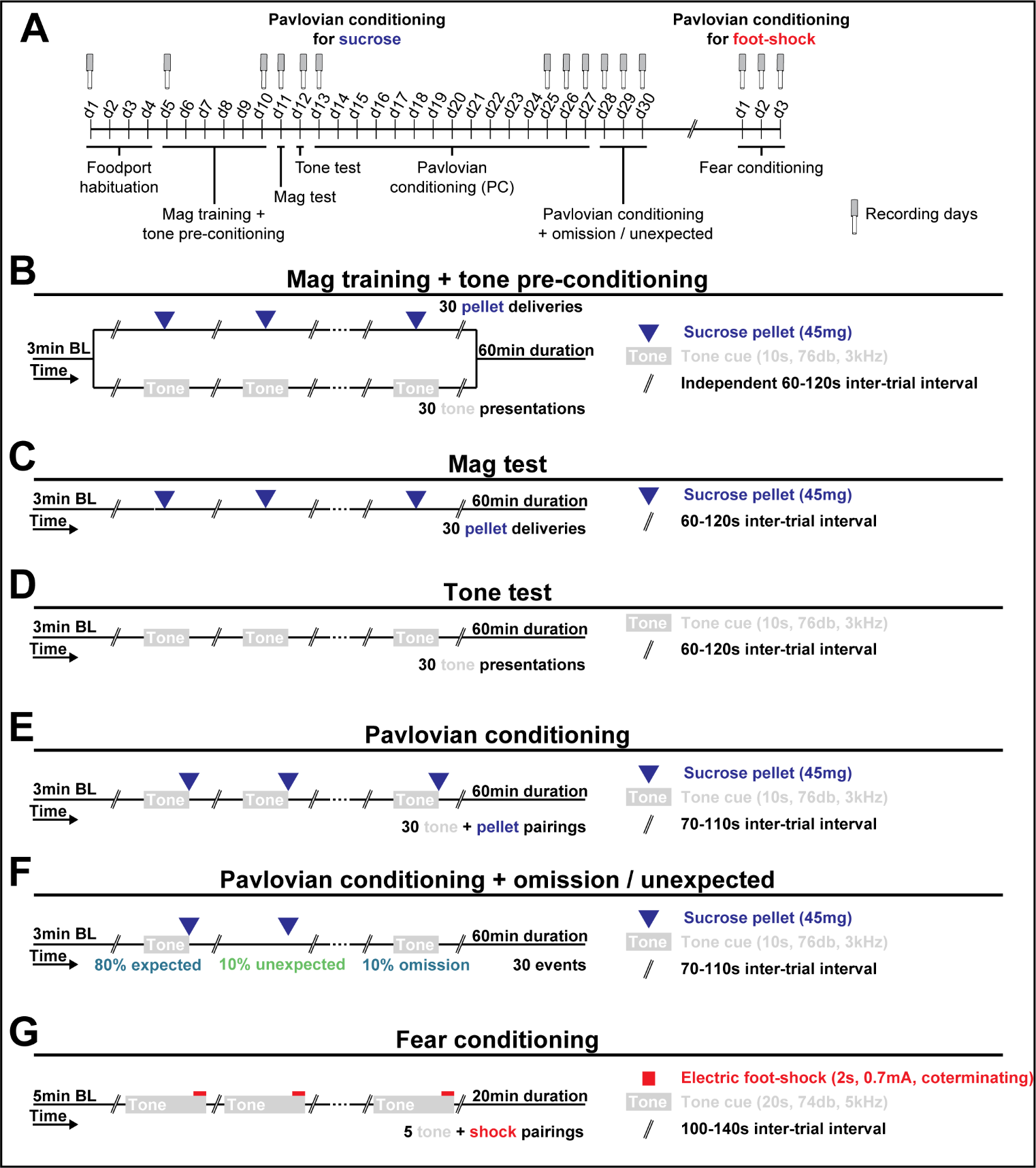


**(A)** Full behavioral timeline for fiber photometry experiments. Fiber icon indicates recording days. **(B-G)** individual behavioral procedures for each procedure within the experiment.

## Supplementary Figure 21: Behavior during Pavlovian conditioning


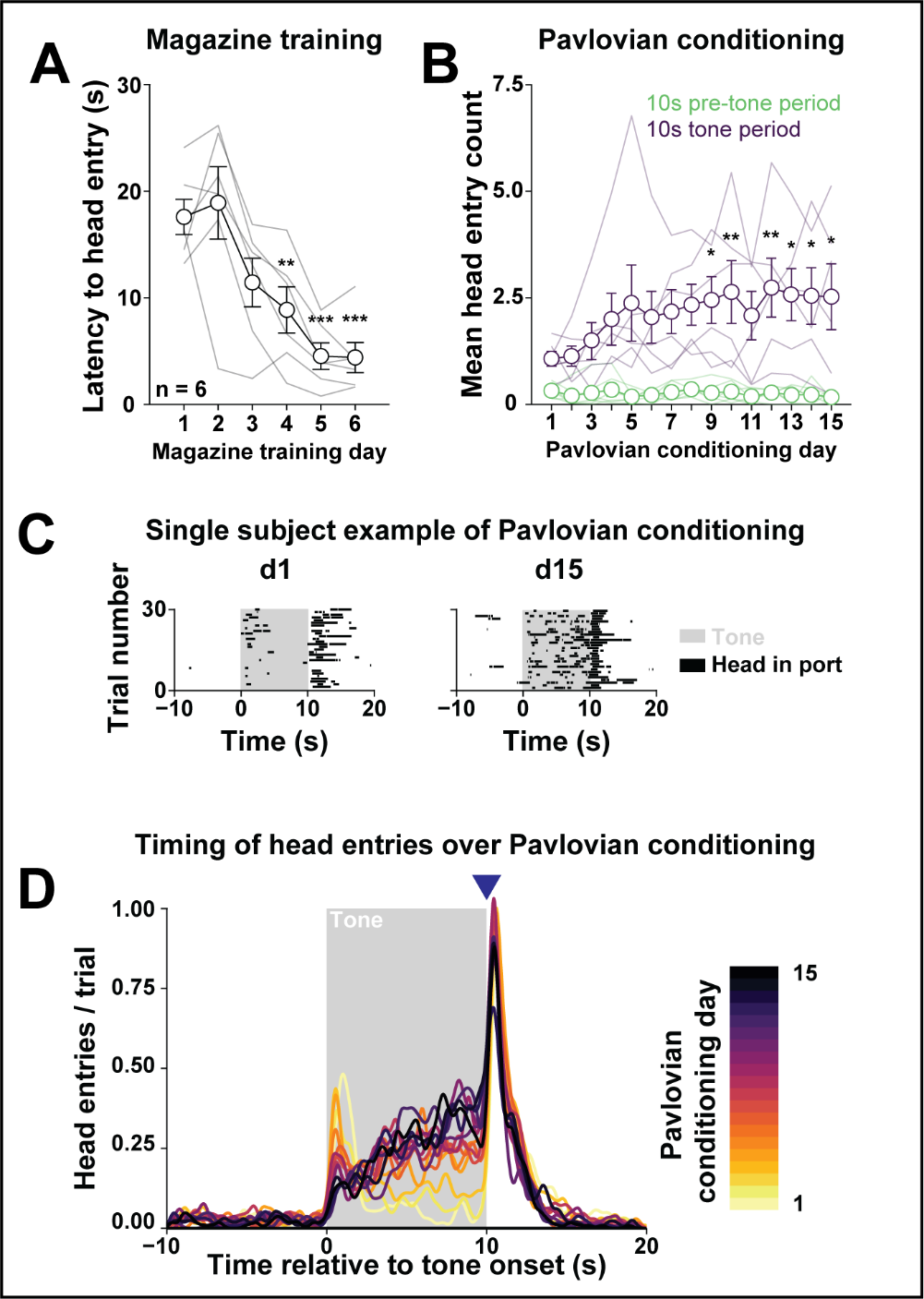


**(A)** Rats showed a decrease in latency from pellet delivery to head entry over the course of magazine training, indicating that rats learned the association between magazine delivery and the presence of sucrose pellets within the food port (indicated by a day effect: F*_5,25_* = 17.73, P < 0.001; HSD, vs. day 1, *: P < 0.05, **: P < 0.01, ***: P < 0.001). **(B)** Rats increase the number of head entries during the tone period (10s) but not the pre-tone period, indicating rats developed a conditioned response to the predictive tone (time period x day interaction: F*_14,70_* = 2.68, P = 0.0034; HSD, vs. day 1, *: P < 0.05, **: P < 0.01, ***: P < 0.001). **(C)** Example of a conditioned response for a single subject. The rat increased the number of head entries during the predictive done from the first day (d1, left) to the day prior to the first fiber photometry recording (d15, right). **(D)** Timing of head entries per trial across training reveals a progressive shift in head entry time relative to the tone. Early in training rats tended to near the start of the tone, but as training progressed, they began to enter during the middle of the tone more often. In (A) and (B), faded lines depict rats’ values; points and error bars depict group mean and sem, respectively.

## Supplementary Figure 22: LPO calcium signaling across Pavlovian conditioning for sucrose and reward expectation


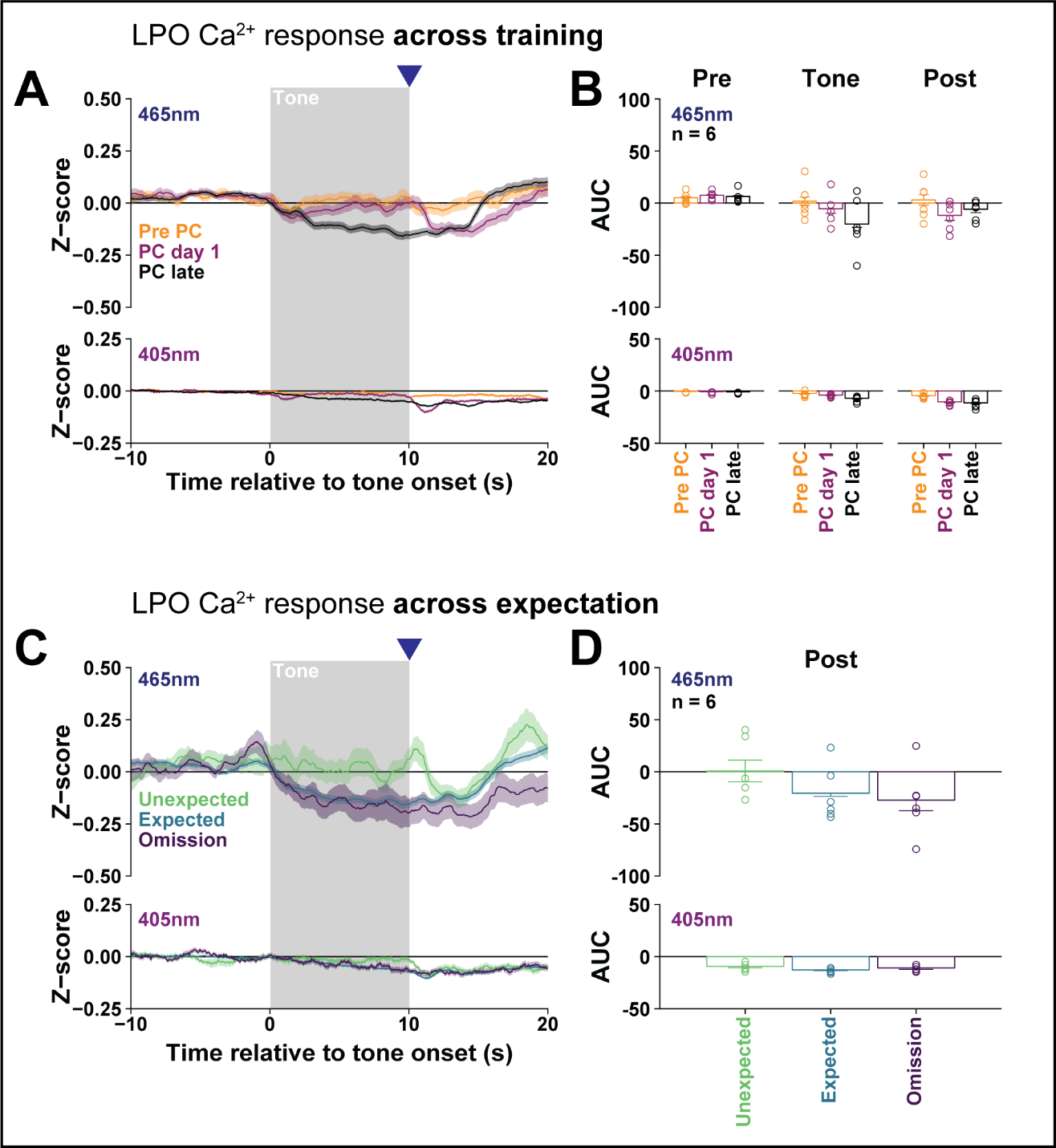


**(A)** LPO 465nm GCaMP (top) and 405nm auto-fluorescence (bottom) signals over the course of training indicates minimal responses in both channels to the tone. On the last day of magazine training + cue preconditioning (“Pre PC”) there were no time-locked changes in either channel. In the case of Pavlovian conditioning for sucrose on the first day (PC day 1) and the last 3 days (PC late), there were minor time-locked decreases in both channels. The simultaneous decrease in both channels may be the result of movement artifacts because they were time-locked with port entry (sup. Figure 21d). **(B)** Area under the curve (AUC) for data shown in (A) indicates no selective changes in the 465nm GCaMP channel in any epoch over training. **(C)** LPO 465nm GCaMP (top) and 405nm auto-fluorescence (bottom) signals over the across expectation indicates minimal reward prediction error encoding. **(D)** AUC during the 10s post pellet period for data shown in (C) indicates no selective changes in the 465nm GCaMP channel across expectation. In (A) and (B), the thick line depicts group mean and shaded ribbon depicts sem; colors indicate the training stage (A) or expectancy (C) as outlined in the inset. In (B) and (D), points depict subject mean AUC and bars with error bars depict group mean and sem, respectively.

## Supplementary Figure 23: LPO calcium signaling across Pavlovian conditioning for foot-shock


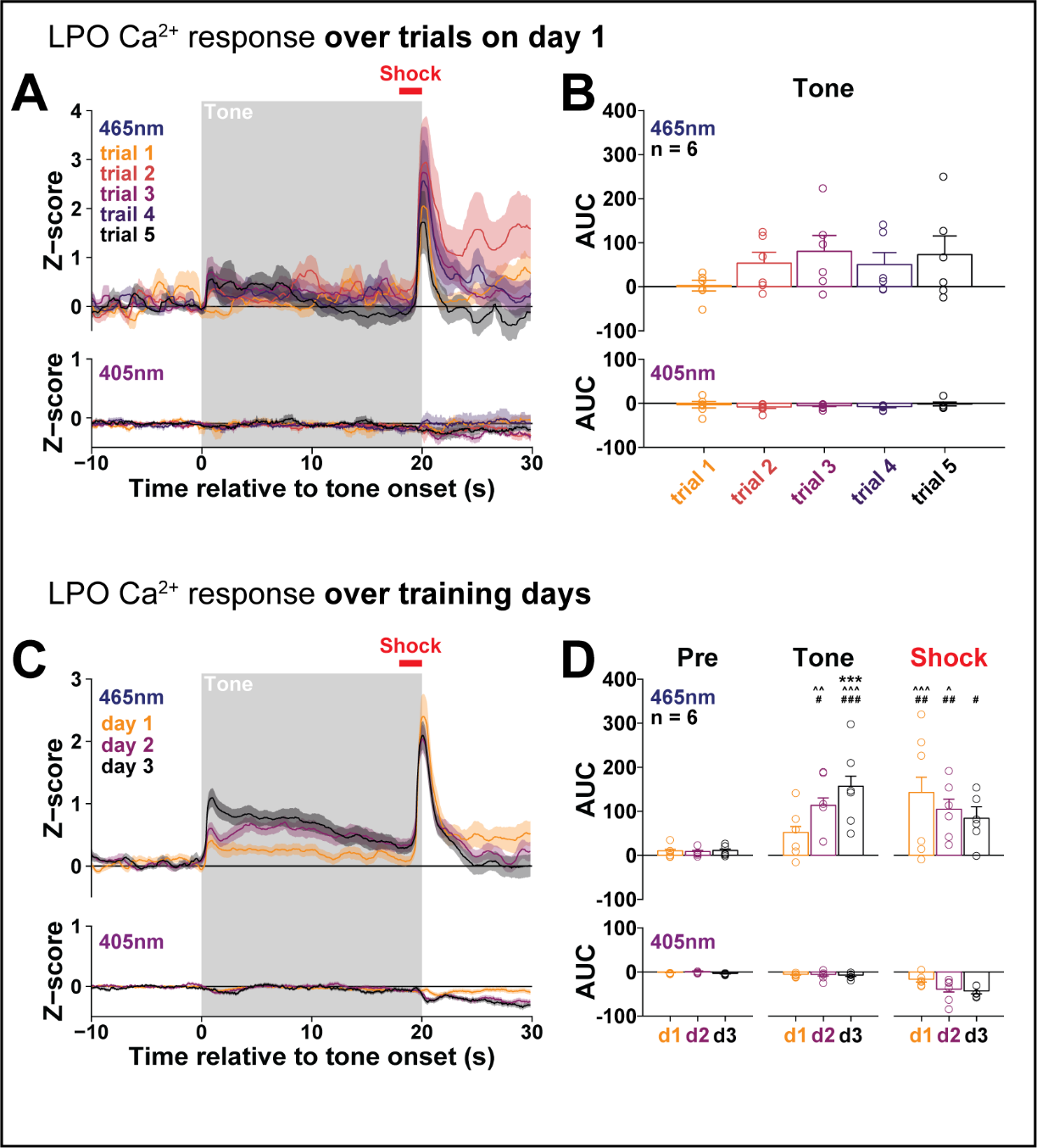


**(A)** LPO 465nm GCaMP (top) and 405nm auto-fluorescence (bottom) over the course of foot-shock conditioning trails on day 1 indicates a trend towards increase in calcium signaling to the tone and an obvious increase in calcium during foot-shock. **(B)** Area under the curve (AUC) for data shown in (A) over the first 10s of the tone indicates no selective changes in the 465nm GCaMP channel in any epoch over training. However, note the lack of tone response on trial 1. **(C)** Over the course of foot-shock conditioning training indicates a clear increase in calcium signaling to the tone. **(D)** AUC for data shown in (C) indicates an increase in calcium in response to the tone and foot-shock on multiple days. Furthermore, there was an increase in the tone response over training days (HSD, vs. d1 ***: P < 0.001; vs. Pre: ^: P < 0.05, ^^: P < 0.01, ^^^: P < 0.001; vs. 405nm: #: P < 0.05, ##: P < 0.01, ###: P < 0.001). In (A) and (B), the thick link depicts group mean and shaded ribbon depicts sem; colors indicate the trial (A) or day (C) as outlined in the inset. In (B) and (D), points depict subject mean AUC and bars with error bars depict group mean and sem, respectively.
